# Design and Synthesis of Novel 6-(Substituted phenyl)-[1,3]dioxolo[4′,5′:4,5]benzo[1,2-*d*]thiazole Compounds as Tyrosinase Inhibitors: In Vitro and In Vivo Insights

**DOI:** 10.3390/molecules30071535

**Published:** 2025-03-30

**Authors:** Hyeon Seo Park, Hee Jin Jung, Hye Soo Park, Hye Jin Kim, Sang Gyun Noh, Yujin Park, Pusoon Chun, Hae Young Chung, Hyung Ryong Moon

**Affiliations:** 1 Department of Manufacturing Pharmacy, College of Pharmacy, Research Institute for Drug Development, Pusan National University, Busan 46241, Republic of Korea; gustj6956@pusan.ac.kr (H.S.P.); hjjung2046@pusan.ac.kr (H.J.J.); hyesoo0713@pusan.ac.kr (H.S.P.); khj3358@pusan.ac.kr (H.J.K.); 2 Department of Pharmacy, College of Pharmacy, Research Institute for Drug Development, Pusan National University, Busan 46241, Republic of Korea; rskrsk92@pusan.ac.kr (S.G.N.);; 3 Department of Medicinal Chemistry, New Drug Development Center, Daegu-Gyeongbuk Medical Innovation Foundation, Daegu 41061, Republic of Korea; pyj1016@kmedihub.re.kr; 4 College of Pharmacy, Inje Institute of Pharmaceutical Sciences and Research, Inje University, Gimhae 50834, Republic of Korea; pusoon@inje.ac.kr

**Keywords:** melanin, tyrosinase, 2,4-dihydroxyphenyl, zebrafish embryos, depigmentation

## Abstract

The 2,4-dihydroxyphenyl group is commonly present in the chemical structures of potent tyrosinase inhibitors. Based on this observation, a series of 6-(substituted phenyl)-[1,3]dioxolo[4′,5′:4,5]benzo[1,2-*d*]thiazole compounds **1**–**13** were designed and synthesized as potential tyrosinase inhibitors. Among these, compounds **5** and **9** strongly inhibited mushroom tyrosinase activity. Particularly, compound **9** exhibited nanomolar IC_50_ values regardless of the substrate used, whereas kojic acid yielded IC_50_ values of 15.99–26.18 μM. Kinetic studies on mushroom tyrosinase revealed that compounds **5** and **9** competitively inhibited tyrosinase activity, findings further corroborated by in silico docking analysis. In B16F10 cell-based experiments, both compounds effectively inhibited the cellular tyrosinase activity and melanin formation. These inhibitory effects were confirmed through in situ cellular tyrosinase activity assays. Compound **9** exhibited strong antioxidant activity by scavenging radicals, suggesting that its ability to reduce melanin production may be attributed to a combination of its antioxidant and tyrosinase inhibitory properties. Additionally, five compounds, including compound **5**, demonstrated effective depigmentation activity in vivo in zebrafish embryos, and their depigmentation efficacy was similar to that of kojic acid, even at concentrations hundreds of times lower. These findings suggest that 6-(substituted phenyl)-[1,3]dioxolo[4′,5′:4,5]benzo[1,2-*d*]thiazole compounds may be promising anti-melanogenic agents.

## 1. Introduction

Melanin is present in nearly all living organisms, including bacteria, fungi, plants, animals, and humans [1]. This widespread presence underscores melanin’s crucial role in various biological processes. In humans, melanin is the primary factor that determines skin, eye, and hair color [2]. Melanin has a chemical structure that absorbs light effectively; therefore, it plays an important role in protecting animals, including humans, by blocking or absorbing harmful ultraviolet rays from sunlight. Although melanin plays a positive role in blocking ultraviolet rays, excessive melanin accumulation or abnormal melanin production in certain areas of the skin can lead to cosmetic concerns like melasma, freckles, age spots, or hyperpigmentation diseases [3,4,5]. Therefore, the demand for skin-whitening agents to address pigmentation-related cosmetic issues and hyperpigmentation diseases is growing.

Melanin is synthesized through reactions involving various enzymes and non-enzymatic chemical reactions [6]. Tyrosinase is a ubiquitous enzyme existing in diverse kingdoms, including bacteria, fungi, insects, plants, and animals, and it plays pivotal roles in mollusk shell formation, insect cuticle sclerotization, bacterial and fungal pigmentation, and bacterial virulence and biofilm formation [7]. Furthermore, as tyrosinase is also involved in melanogenesis and acts as a rate-limiting enzyme in melanin biosynthesis, developing compounds that inhibit tyrosinase to control melanin biosynthesis is an active research topic [8,9]. Melanin is synthesized in melanosomes within melanocytes present in the epidermis and transferred to keratinocytes. Skin color is primarily determined by the ratio of pheomelanin and eumelanin. Tyrosinase is an oxidizing enzyme with monophenolase and diphenolase activities [10]. Monophenolase oxidizes l-tyrosine to l-dopa, and diphenolase oxidizes l-dopa to dopaquinone. Therefore, in experiments evaluating the tyrosinase inhibitory activity of compounds, l-tyrosine or l-dopa is used as the substrate [11]. To date, many tyrosinase inhibitors have been identified. Some of them, classified by category, are as follows: flavonoids [12,13], coumarins [14,15,16], thioureas [17,18], kojic acids [19,20], chalcones [21,22], stilbenes [23,24], β-phenyl-α,β-unsaturated carbonyls [25,26,27,28,29], curcuminoids [30,31], thiosemicarbazones [32,33,34], thiosemicarbazides [35,36], azoles [37], and thiols [38]. Although numerous tyrosinase inhibitors have been identified, only a few tyrosinase inhibitors are currently used clinically as skin-whitening agents due to side effects such as carcinogenicity and insufficient efficacy in clinical trials. Therefore, the development of melanin production inhibitors with novel structures that do not fall into the category of existing tyrosinase inhibitors is required.

**Figure 1:**
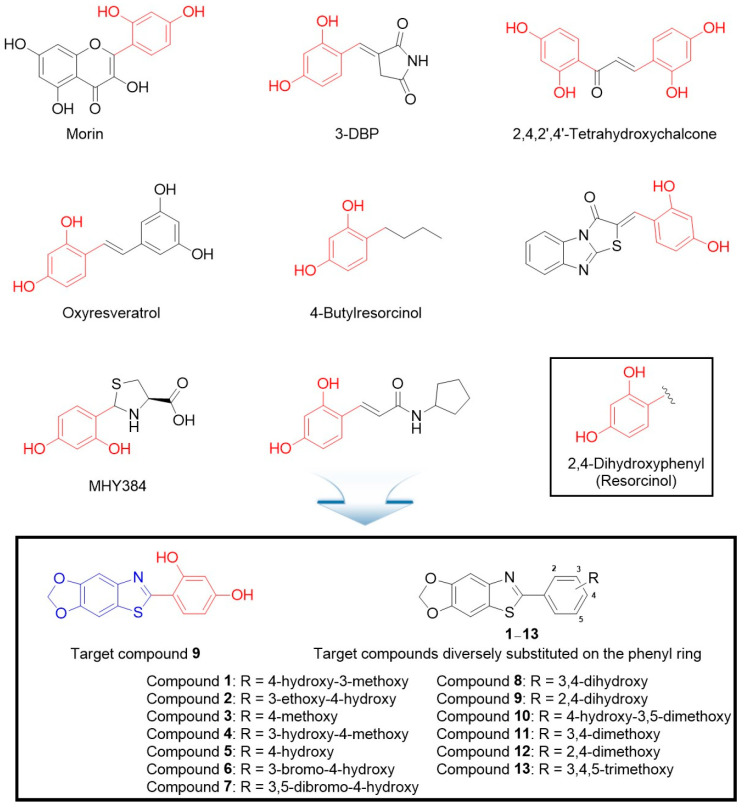
Chemical structures of a 2,4-dihydroxyphenyl moiety, tyrosinase inhibitors bearing a 2,4-dihydroxyphenyl moiety, and target compounds **1**–**13**. The 2,4-dihydroxyphenyl moiety is presented in red.

The 2,4-dihydroxyphenyl (resorcinol) structure commonly exists in the chemical structures of compounds known as excellent tyrosinase inhibitors (Figure 1) [39,40,41,42,43,44,45,46]. Therefore, compound **9**, with a 2,4-dihydroxyphenyl structure, was designed as a potential tyrosinase inhibitor. Additionally, the structure–activity relationship was studied by modifying the substituents on the phenyl ring of compound **9**. Compounds **1**–**13** were synthesized, most of which were unknown. The inhibitory activity of the synthesized compounds on mushroom tyrosinase was screened, and the inhibitory mechanism was elucidated through kinetic experiments. Moreover, the cellular tyrosinase and melanin production inhibitory activities of the compounds were investigated in B16F10 cells, a mammalian cell line. The strong mushroom and B16F10 cellular tyrosinase inhibitory activities of the compounds prompted in vivo depigmentation experiments in zebrafish embryos.

## 2. Results and Discussion

### 2.1. Preparation of Target Compounds, 6-(substituted phenyl)-[1,3]dioxolo[4′,5′:4,5]benzo[1,2-d]thiazole ***1***–***13***

**Scheme 1:**
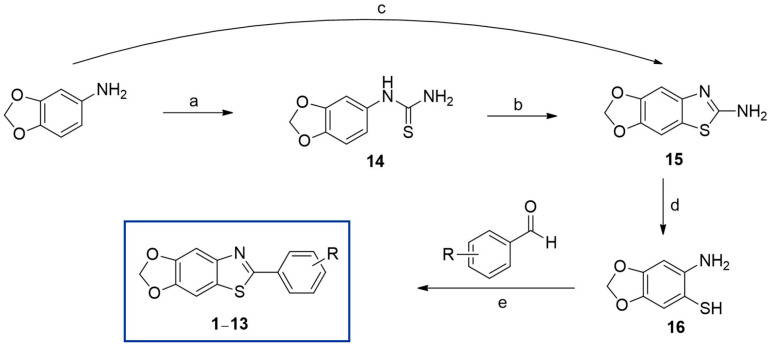
Synthetic route of target compounds **1**–**13**. Reagents and conditions: (a) (i) NH_4_SCN, benzoyl chloride, acetone, 50 °C, 2 h; (ii) 2N–NaOH solution, 100 °C, 1 h, 48% (two-step yield). (b) (i) Br_2_, AcOH, room temperature (RT), 40 min; (ii) 25% NH_4_OH, RT, 5 min, 86% (two-step yield). (c) (i) KSCN, AcOH, RT, 30 min; (ii) Br_2_, AcOH, RT, 22 h; (iii) 25% NH_4_OH, RT, 5 min, 81% (three-step yield); (d) KOH, H_2_O:2-methoxyethanol (1:1), reflux, 2 h, 55%; and (e) Na_2_S_2_O_5_, DMF, 80 °C, 1.5–4.5 h, 60–84%.

As depicted in Scheme 1, 6-aminobenzo[*d*][1,3]dioxole-5-thiol (**16**) can be used as a key intermediate for the preparation of the final compounds **1**–**13**. For the synthesis of compound **16** [47], 3,4-(methylenedioxy)aniline was mercaptized at position 6 via a two- or three-step reaction. 3,4-(Methylenedioxy)aniline was first converted into thiourea compound **14** by reacting with benzoyl isothiocyanate, which was obtained by reacting ammonium isothiocyanate with benzoyl chloride in acetone and hydrolyzing the benzoyl group. Treating compound **14** with bromine in acetic acid produced the HBr salt of compound **15**, which was subsequently converted to HBr-free **15** using 25% ammonium hydroxide. The yield of **15** from 3,4-(methylenedioxy)aniline was relatively low, at 41%. Another method for synthesizing compound **15** [48,49] was attempted. 3,4-(Methylenedioxy)aniline was reacted with potassium isothiocyanate in acetic acid to produce compound **14**. Compound **14** was then reacted with bromine in acetic acid to yield the HBr salt of compound **15**, which was subsequently treated with 25% ammonium hydroxide to obtain HBr-free **15**. The total yield of **15** was improved to 81%. To obtain key intermediate **16**, **15** was hydrolyzed by refluxing **15** with excess KOH (20 equiv.) in a cosolvent of water and 2-methoxyethanol (1:1), providing a 55% yield. The oxidative cyclization of **16** with various substituted benzaldehydes in Na_2_S_2_O_5_ produced the target compounds **1**–**13** in yields of 60–84%. The structures of the synthesized compounds were confirmed using ^1^H- and ^13^C-NMR and high-resolution mass spectroscopy.

### 2.2. Mushroom Tyrosinase-Inhibitory Activity of Target Compounds ***1***–***13***

The tyrosinase inhibitory activities of target compounds **1**–**13** were evaluated using mushroom tyrosinase. In the inhibition experiments, l-dopa and l-tyrosine were used as enzyme substrates. For determining their IC_50_ values, the inhibitory activity for each compound was measured at three different concentrations (0.08, 0.4, and 2 μM or 2, 10, and 50 μM with l-dopa; 0.02, 0.08, and 0.4 μM or 2, 10, and 50 μM with l-tyrosine: Supplementary Materials: S46–S50). Kojic acid was used as a positive substance to compare IC_50_ values.

When l-dopa was utilized as an enzyme substrate, kojic acid revealed potent tyrosinase inhibitory activity, with an IC_50_ value of 26.18 μM. Compound **1** with 4-hydroxy-3-methoxyphenyl exhibited only 1.7% inhibition at 50 μM, although the change in the 3-methoxy group to a 3-ethoxy group greatly increased the tyrosinase inhibitory activity (IC_50_ value of **2**: 49.73 μM). The exchange of the hydroxyl and methoxyl group in compound **1** also greatly enhanced the tyrosinase inhibitory activity (IC_50_ value of **4**: 40.89 μM). However, removal of the 3-hydroxyl group of **4** again decreased the tyrosinase inhibitory activity (percent inhibition of **3** at 50 μM: 17.2%). Removal of the 3-methoxyl group of **1** greatly increased the tyrosinase inhibitory activity (IC_50_ value of **5**: 36.12 μM), which was comparable to that of kojic acid. Meanwhile, insertion of bromine at position 3 or positions 3 and 5 of compound **5** reduced the tyrosinase inhibitory activity (percent inhibition of **6** and **7** at 50 μM: 27.9 and 29.7%, respectively). Remarkably, the introduction of an additional hydroxyl group on the phenyl ring of **5** resulted in varying inhibitory activities depending on its position. The addition of a hydroxyl group at position **3** greatly decreased the inhibitory activity (percent inhibition of **8** at 50 μM: 18.6%), whereas the addition of hydroxyl group at position 2 greatly increased the tyrosinase inhibitory activity (IC_50_ value of **9**: 0.74 μM). The IC_50_ value of **9** containing 2,4-dihydroxyphenyl was 35 times lower than that of kojic acid. Although compound **1** bearing a 4-hydroxy-3-methoxyphenyl group showed little tyrosinase inhibitory activity, the addition of methoxyl group at position 5 on the phenyl ring of **1** produced **10**, which exhibited improved tyrosinase inhibitory activity, with an IC_50_ value of 50.13 μM. Compound **9** bearing 2,4-dihydroxyphenyl exhibited highly potent tyrosinase inhibitory activity, whereas compound **12** bearing 2,4-dimethoxyphenyl exhibited only moderate tyrosinase inhibitory activity, with an inhibition of 32.8% at 50 μM. Similarly, **11** bearing 3,4-dimethoxyphenyl and **13** bearing 3,4,5-trimethoxyphenyl, which do not have a hydroxyl group, revealed only very weak tyrosinase inhibitory activity (percent inhibition of **11** and **13** at 50 μM: 19.5 and 5.0%, respectively).

When l-tyrosine was utilized as an enzyme substrate, kojic acid revealed potent tyrosinase inhibitory activity, with an IC_50_ value of 15.99 μM. Most of the synthesized compounds showed less than 30% inhibition at 50 μM, except compounds **5** and **9**. Compound **5** with 4-hydroxyphenyl exhibited strong tyrosinase inhibitory activity, with an IC_50_ value of 34.21 μM, similar to that of compound **5** with l-dopa. Compound **9** containing 2,4-dihydroxyphenyl exhibited the strongest tyrosinase inhibitory activity with l-tyrosine. Compound **9** revealed highly potent inhibitory activity, with an IC_50_ value of 0.24 μM, which was 67 times lower than that of kojic acid. When the IC_50_ value of compound **9** was compared with the IC_50_ values of recently reported kojic acid-fused 2-amino-3-cyano-4*H*-pyran derivatives, compound **9** exhibited an IC_50_ value that was at least 10-fold lower than that of the kojic acid-fused derivatives [50]. Furthermore, compound **9** demonstrated fungal diphenolase inhibitory activity that was comparable to, if not more potent than, that of resorcinol alkylglucosides [51].

**Figure 2:**
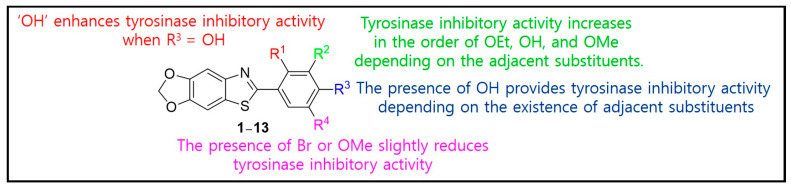
Illustration of the structure–tyrosinase inhibitory activity relationship of compounds **1**–**13** obtained from Table 1.

As shown in Figure 2, analysis of the structure–tyrosinase inhibitory activity relationship of compounds **1**–**13** obtained from Table 1 revealed that the hydroxyl substituent at R^1^ greatly enhanced the tyrosinase inhibitory activity when OH was present at R^3^. The hydroxyl substituent at R^3^ conferred tyrosinase inhibitory activity, although this depended on adjacent substituents, and substituents (Br and OMe) on R^4^ weakly decreased tyrosinase inhibitory activity. The substituents at R^2^ generally enhanced the tyrosinase inhibitory activity in the order of OEt, OH, and OMe, depending on the adjacent substituents.

### 2.3. Kinetic Study on Mushroom Tyrosinase Through Analyzing Lineweaver–Burk Plots

Compounds **5** and **9** strongly inhibited the mushroom tyrosinase activity. Therefore, the mechanism of inhibition was investigated using mushroom tyrosinase. At various concentrations of l-dopa, the initial dopachrome production rate was investigated at four different concentrations of **5** (0, 20, 40, and 80 μM) and **9** (0, 0.5, 1.0, and 2.0 μM).

**Figure 3:**
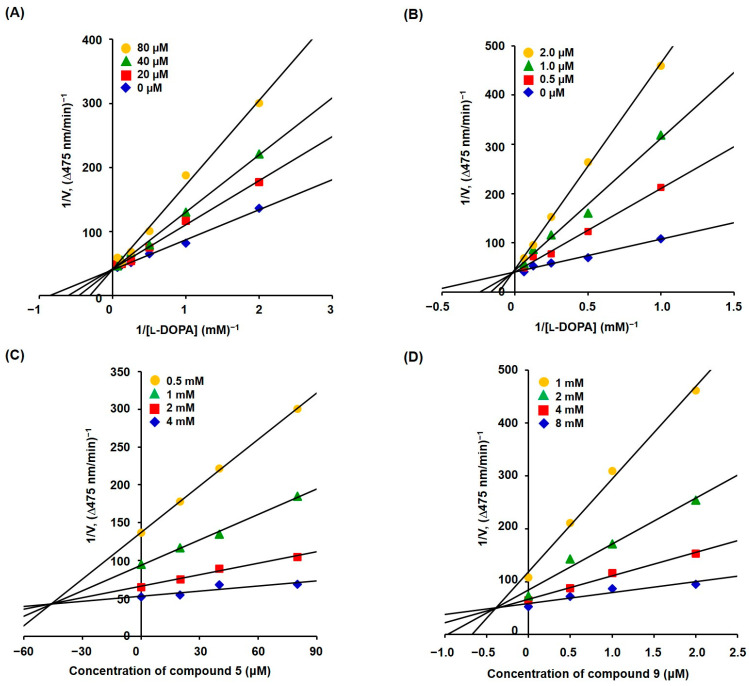
Lineweaver*–*Burk plots ((**A**) for **5** and (**B**) for **9**) and Dixon plots ((**C**) for **5** and (**D**) for **9**) obtained by measuring the initial dopachrome formation rate at four different concentrations of **5** and **9** (0, 20, 40, and 80 μM for **5** and 0, 0.5, 1.0, 2.0 μM for **9**) at various l-dopa concentrations.

The kinetic study results for compounds **5** and **9** are presented in Figure 3A and Figure 3B, respectively. The Lineweaver–Burk plot for each compound provided four straight lines with different slopes that merged at one point on the y-axis, indicating that both compounds were competitive inhibitors that bound competitively to the tyrosinase active site, along with the substrates l-dopa and l-tyrosine. The Lineweaver–Burk plot for each compound was re-plotted by plotting the reciprocal of the initial dopachrome production rate vs. the reciprocal of the compound concentration to create a Dixon plot for each compound (Figure 3C,D for compounds **5** and **9**, respectively). Each Dixon plot produces four straight lines that merge at a single point in the second quadrant. The absolute value of the x-coordinate of the merged point represents the inhibition constant (K_i_), which indicates the potency of the inhibitor. The K_i_ values of **5** and **9** were 4.6 × 10^−5^ and 3.8 × 10^−7^ M, respectively. Thus, compound **9** could effectively inhibit mushroom tyrosinase activity at considerably lower concentrations than compound **5**.

### 2.4. Docking Simulation of Mushroom Tyrosinase and Compounds ***5*** and ***9***

As compounds **5** and **9** exhibited strong mushroom tyrosinase inhibitory activity, they were docked into mushroom tyrosinase using the AutoDock Vina software (The Scripps Research Institute, La Jolla, CA, USA) to investigate their interaction modes. The 3D structure of tyrosinase was acquired from the RCSB Protein Data Bank (PDB). The 3D structure of *Agaricus bisporus* (PDB: 2Y9X) was used as the protein structure for the in silico docking simulation [52]. Kojic acid was used as a positive reference compound. The 3D structures of the test ligands (**5**, **9**, and kojic acid) were created using Chem3D Pro 12.0 (https://cs-chemdraw-pro.software.informer.com; accessed on 10 November 2024).

We had performed a validation procedure prior to docking simulations between target compounds **1**–**13** and mushroom tyrosinase. The co-crystallized ligand of the code 2Y9X protein is tropolone. Therefore, for validation of the docking procedure, we performed a redocking of the co-crystallized tropolone to tyrosinase. The redocking results showed that the redocked tropolone reproduced the binding pose with a binding affinity of −5.5 kcal/mol (Supplementary Materials: S44). The RMSD of the co-crystallized and experimental poses was analyzed, and the RMSD value was 0.61 Å. These results suggest that the docking simulation could properly accommodate the crystallized ligand in the tyrosinase active site.

**Figure 4:**
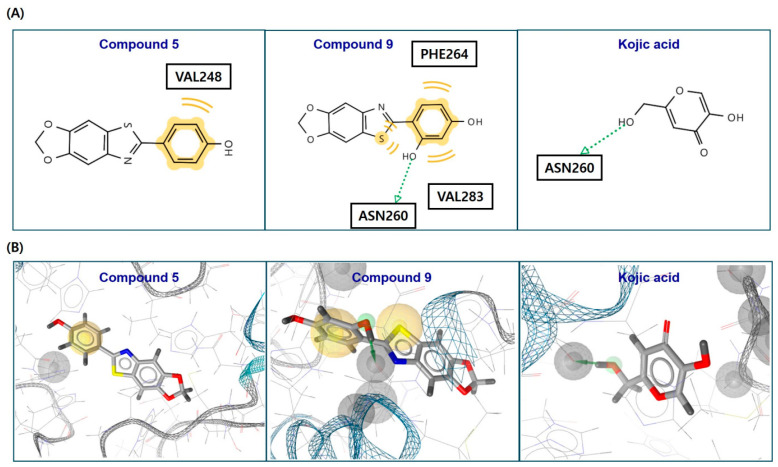
2D (**A**) and 3D (**B**) images illustrate the chemical interactions between the ligands (**5**, **9**, and kojic acid) and mushroom tyrosinase, obtained from in silico docking simulations using the AutoDock Vina and LigandScout software. (**A**) Yellow wave and green arrows represent hydrophobic interactions and hydrogen bonds, respectively. (**B**) Red, blue, and yellow in the ligand structure represent the oxygen, nitrogen, and sulfur atoms, respectively.

After docking simulation using the AutoDock Vina software, possible chemical interactions between the ligand (**5**, **9**, and kojic acid) and tyrosinase were determined using the LigandScout 4.4.8 software (Inte:Ligand, Vienna, Austria) [53]. Possible chemical interactions are presented in Figure 4. All test ligands were bound to the tyrosinase active site. The phenyl ring of compound **5** formed a hydrophobic interaction with Val248 amino acid residue. Compound **9** interacted with the residues of three amino acids. The phenyl ring hydrophobically interacted with Phe264 and Val283, and Val283 also had hydrophobic interaction with the sulfur of the thiazole ring. Moreover, the 2-hydroxyl group of the phenyl ring was hydrogen-bonded with Asn260 as a hydrogen bond donor. Similar to **9**, kojic acid formed a hydrogen bond with Asn260 as the hydrogen bond donor; however, no other interactions were observed. These chemical interactions provided **5**, **9**, and kojic acid with binding energies of −6.0, −6.3, and −5.4 kcal/mol, respectively. Thus, compounds **5** and **9** could tightly bind to the tyrosinase active site, thereby inhibiting tyrosinase activity.

### 2.5. Cell Viability of Compounds ***5*** and ***9*** on B16F10 Cells

As compounds **5** and **9** strongly inhibited mushroom tyrosinase activity, their ability to inhibit mammalian cellular tyrosinase activity and melanin production in cells was investigated. Prior to these cell-based experiments, the cytotoxicity of the compounds was assessed using EZ-Cytox solution and B16F10 murine cells. The cells were treated with three different concentrations (1, 2, and 5 μM) of **5** and **9** for 72 h, and cell viability was determined 2 h after treatment with the EZ-Cytox solution.

**Figure 5:**
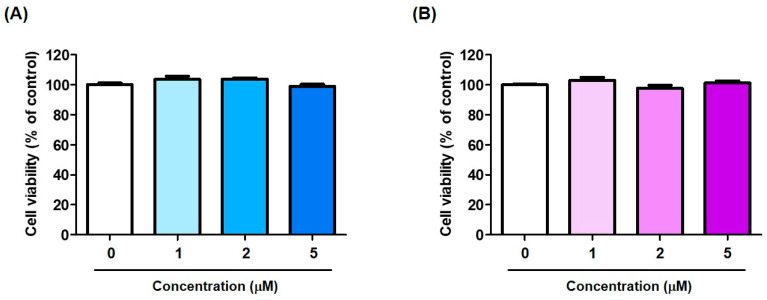
Effects of compounds **5** (**A**) and **9** (**B**) on B16F10 cell viability obtained at 0, 1, 2, and 5 μM for 72 h using an EZ-Cytox solution.

None of the compounds exhibited significant cytotoxicity at any tested concentration (Figure 5). Therefore, cell-based experiments performed on B16F10 cells were conducted at concentrations ≤ 5 μM for 72 h.

### 2.6. Effects of Compounds ***5*** and ***9*** on B16F10 Cellular Tyrosinase Activity

The potencies of compounds **5** and **9** in inhibiting mammalian cellular tyrosinase activity were evaluated using B16F10 cells. Compounds **5** and **9** and kojic acid (positive control) were administered at 1, 2, and 5 μM, respectively, 1 h before treatment with stimulators (3-isobutyl-1-methylxanthine [IBMX; 200 μM] and α-melanocyte-stimulating hormone [α-MSH; 1 μM]). After 72 h of incubation, the B16F10 cellular tyrosinase inhibitory activity was evaluated by measuring the optical density at 475 nm.

**Figure 6:**
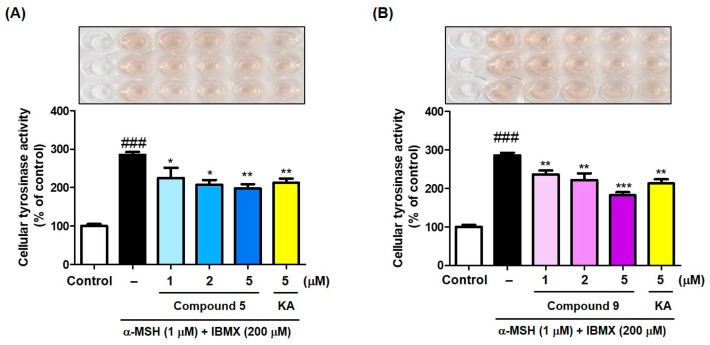
Cellular tyrosinase activity of **5** (**A**) and **9** (**B**) obtained at 1, 2, and 5 μM. Kojic acid (KA; 5 μM) was used as a positive control for comparing activity. Test samples (**5**, **9**, and KA) were treated for 72 h, and 200 μM IBMX and 1 μM α-MSH were exposed 1 h after treatment with test samples. *** *p* < 0.001, ** *p* < 0.01, and * *p* < 0.05 vs. IBMX plus α-MSH-treated group; ^###^ *p* < 0.001 vs. untreated group.

Exposure to the stimulators greatly enhanced cellular tyrosinase activity compared with the control (100%), as shown in Figure 6. Compounds **5** and **9** significantly decreased the cellular tyrosinase activity enhanced by treatment with stimulators in a dose-dependent manner. In the mushroom tyrosinase inhibition experiment, compound **9** showed an IC_50_ value that was about 100-fold lower than that of compound **5**, but showed similar inhibitory activity to compound **5** in the B16F10 cellular tyrosinase inhibition experiment. This is probably due to the structural differences between mushroom tyrosinase and murine tyrosinase and the Log *P* differences between the two compounds. The Log *P* values of compounds **5** and **9** estimated by ChemDraw Ultra 12.0 (CambridgeSoft Corporation, Cambridge, MA, USA) were 3.69 and 3.3, respectively. Compound **5**, which is more lipophilic than compound **9**, may have been better absorbed into cells, which may have contributed in part to the result that the cellular tyrosinase inhibitory activity of compound **5** was similar to that of compound **9**. Each compound significantly reduced cellular tyrosinase activity even at 1 μM, and kojic acid at 5 μM inhibited cellular tyrosinase activity similar to compounds **5** and **9** at 2 μM. The tyrosinase inhibitory activity of these compounds led to an investigation of their effects on melanin production in B16F10 cells.

### 2.7. Effects of Compounds ***5*** and ***9*** on Melanogenesis in B16F10 Cells

As compounds **5** and **9** reduced B16F10 cellular tyrosinase activity, their ability to inhibit melanin production in B16F10 cells was examined using kojic acid (5 μM) as a positive material. As in cellular tyrosinase experiments, compounds **5** and **9** (1, 2, and 5 μM) and kojic acid (5 μM) were administered 1 h before treatment with stimulators (IBMX plus α-MSH; 200 and 1 μM, respectively). After 72 h of incubation, the melanin content was evaluated by measuring the optical density at 405 nm.

**Figure 7:**
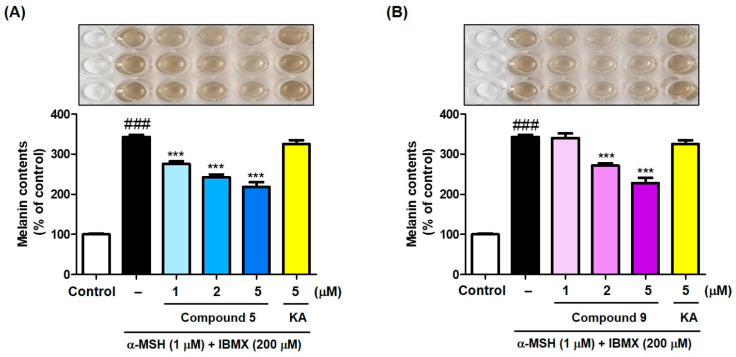
Effects of **5** (**A**) and **9** (**B**) on melanin content obtained at 1, 2, and 5 μM. Kojic acid (KA; 5 μM) was used as a positive control for comparing activity. Test samples (**5**, **9**, and KA) were treated for 72 h, and 200 μM IBMX and 1 μM α-MSH were exposed 1 h after treatment with test samples. *** *p* < 0.001 vs. IBMX plus α-MSH-treated group; ^###^ *p* < 0.001 vs. untreated group.

Exposure to stimulators greatly raised melanin content compared with the control (100%), as shown in Figure 7. Compounds **5** and **9** significantly reduced the stimulator-induced melanin contents in a concentration-dependent manner. In particular, each compound inhibited melanin production more potently than kojic acid at the same concentration (5 μM). Compound **9** showed inhibitory activity 100 times stronger than compound **5** in the mushroom tyrosinase inhibition experiments. However, possibly due to the structural differences between mushroom tyrosinase and murine tyrosinase and the Log *P* differences of **5** and **9**, the two compounds showed similar cellular tyrosinase inhibition effects in B16F10 murine cells. Therefore, both compounds exhibited inhibitory activity against melanin production in B16F10 murine cells similar to that in the cellular tyrosinase inhibition experiment.

### 2.8. Effects of Compounds ***5*** and ***9*** on In Situ B16F10 Cell Tyrosinase Inhibition

The effect of compounds **5** and **9** on in situ B16F10 cell tyrosinase inhibition was studied using kojic acid (5 μM) as a positive control. As in B16F10 cell-based experiments, compounds **5** and **9** (1, 2, and 5 μM) and kojic acid (5 μM) were administered 1 h before treatment with stimulators (IBMX plus α-MSH; 200 and 1 μM, respectively). After 72 h of incubation, the cells were treated with an excessive amount of l-dopa for 2 h, and then the melanin-stained cells were photographed, and the pigmented areas of the cells were determined using an ATTO CS Analyzer (ATTO, Tokyo, Japan).

**Figure 8:**
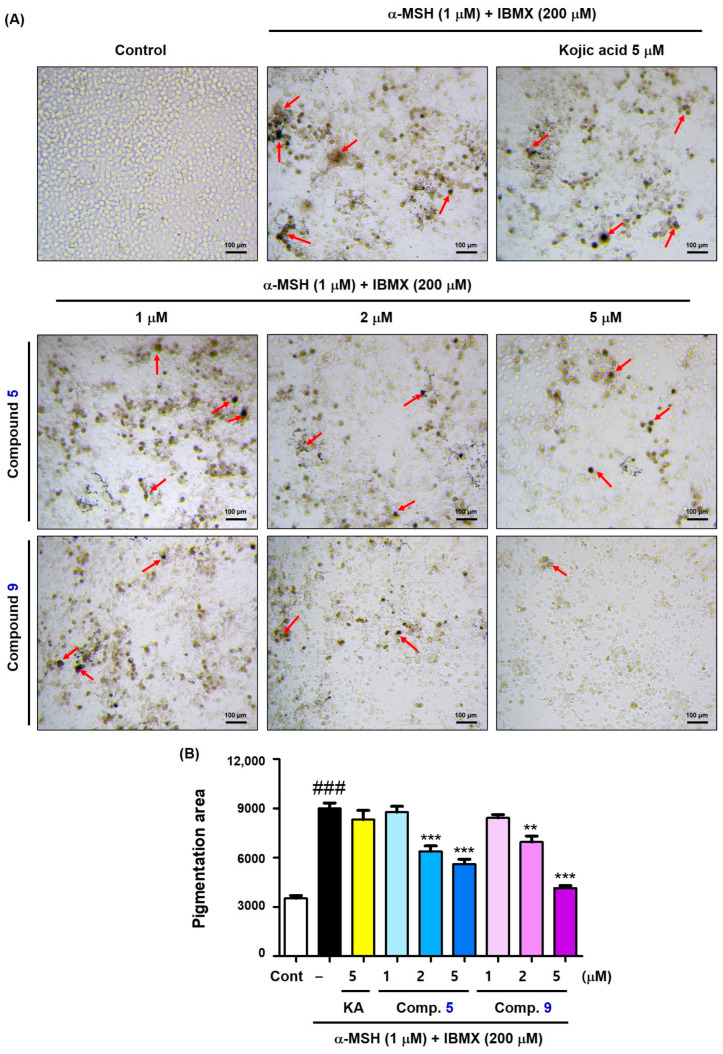
B16F10 cell tyrosinase inhibitory activity of compounds **5** and **9** determined in situ. (**A**) Images of cells stained with melanin. Bar = 100 μM. (**B**) Pigmented areas of cells stained with melanin. KA and Comp. refer to kojic acid and compound, respectively. Compounds **5** and **9** and kojic acid were administered at 1, 2, and 5 μM and 5 μM, respectively, and after 1 h, IBMX (200 μM) and α-MSH (1 μM) were exposed. In situ tyrosinase inhibition results were obtained 72 h after treatment with test samples. *** *p* < 0.001 and ** *p* < 0.01 vs. IBMX plus α-MSH-treated group; ^###^ *p* < 0.001 vs. non-treated group. Arrows represent cells strongly stained with melanin.

As depicted in Figure 8A (images of melanin-stained cells) and Figure 8B (pigmented areas of cells), exposure to stimulators produced a number of cells strongly stained with melanin compared with the control. Treatment with kojic acid slightly reduced the number of cells strongly stained with melanin. Conversely, exposure to compounds **5** and **9** evidently reduced the number of cells strongly stained with melanin in a concentration-dependent manner. Particularly, at a concentration of 5 μM, the melanin production-inhibitory potency of **9** was greater than that of **5**. Even at the low concentrations ≤ 5 μM, compounds **5** and **9** could effectively inhibit B16F10 cell tyrosinase activity. In contrast to the results of the mushroom tyrosinase inhibition experiment, both compounds showed similar murine tyrosinase inhibition activity in the in situ tyrosinase inhibition activity experiment in B16F10 cells.

### 2.9. Antioxidant Effects of Compounds ***5*** and ***9*** on 2,2-Diphenyl-1-picrylhydrazyl (DPPH) and 2,2′-Azino-bis(3-ethylbenzothiazoline-6-sulfonic acid) (ATBS)^+^ Radicals

The antioxidant ability of a compound may influence melanin production [54,55]. Therefore, the antioxidant efficacy of compounds **1**–**13** was assessed using ABTS^+^ and DPPH radicals.

The ABTS^+^ radical solution was obtained by mixing ABTS and potassium persulfate, an oxidizing reagent, and test samples (**1**–**13** and Trolox [positive control]; final concentration: 100 μM) were added to determine their ABTS^+^ radical scavenging ability by measuring the optical density at 732 nm.

**Figure 9:**
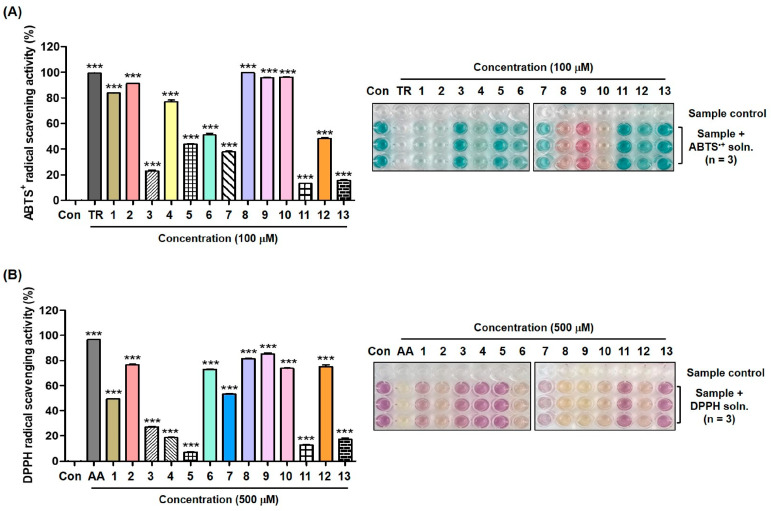
Antioxidant effect of compounds **1**–**13** on scavenging ABTS^+^ (**A**) and DPPH (**B**) radicals. (**A**) All test samples (**1**–**13** and Trolox [TR]) were administered at 100 μM, and ABTS^+^ radicals were generated by oxidizing ABTS with potassium persulfate. (**B**) All test samples (**1**–**13** and ascorbic acid [AA, positive control]) were administered at 500 μM. *** *p* < 0.001 vs. non-treated control. DPPH, 2,2-diphenyl-1-picrylhydrazyl; ATBS^+^, 2,2′-azino-bis(3-ethylbenzothiazoline-6-sulfonic acid)^+^.

ABTS^+^ radical scavenging assay results are depicted in Figure 9A. Of the thirteen synthesized compounds, six compounds (**1**, **2**, **4**, and **8**–**10**) exhibited strong ABTS^+^ radical scavenging activities of >77%. In particular, compounds **8**–**10** containing 3,4-dihydroxyphenyl, 2,4-dihydroxyphenyl, and 4-hydroxy-3,5-dimethoxyphenyl, respectively, inhibited ABTS^+^ radicals with inhibition rates of 99%, 96%, and 96%, respectively, similar to that of Trolox (99% inhibition). In general, compounds without hydroxyl substituents revealed lower ABTS^+^ radical scavenging activities than those with more hydroxyl substituents.

DPPH radical scavenging activity was determined by mixing the DPPH solution with compounds **1**–**13**, leaving it in the dark at 20 °C for 30 min, and measuring the optical density at 517 nm. Ascorbic acid (AA) was used as a positive control to compare the scavenging activity. All test samples were used at 500 μM.

The DPPH radical scavenging results are depicted in Figure 9B. Treatment with AA resulted in the most potent DPPH radical scavenging ability, with 96% inhibition. Of the thirteen synthesized compounds, six inhibited DPPH radicals by >73%, and compounds **8** and **9**, which contained 3,4-dihydroxyphenyl and 2,4-dihydroxyphenyl, respectively, exhibited the strongest DPPH radical scavenging potency of 82% and 85%, respectively. Thus, the antioxidant potency of **9** may contribute to its anti-melanogenic efficacy to some extent.

### 2.10. Depigmentation Effect of Compounds on Zebrafish Larvae

To identify whether the synthesized compounds could exert anti-melanogenic effects in vivo, in vivo experiments were performed using zebrafish embryos [56]. Freely obtained zebrafish embryos were transferred to a culture dish containing an E3-MB solution and incubated at 28 °C until use. For a preliminary experiment, zebrafish embryos were dechorionated using Pronase^®^ (Sigma-Aldrich) at 20 h post fertilization (hpf), and each compound at 0.1 mM was exposed to five zebrafish embryos in E3 solution at 24 hpf. The depigmentation abilities were compared at 72 hpf to select the compounds used in the main experiments. From the preliminary experiments (*n* = 1), five compounds (**2**, **5**, and **11**–**13**) were selected for the main depigmentation experiments (*n* = 3). As in the preliminary experiment, the obtained zebrafish embryos were dechorionated at 20 hpf, and each selected compound (0.1 mM) was administered to six zebrafish embryos in the E3 solution at 24 hpf. The pigmentation of zebrafish larvae was photographed and measured in dorsal and lateral views at 72 hpf using an ATTO CS analyzer (ATTO, Tokyo, Japan). Kojic acid was used as a positive control to compare depigmentation activity, and the concentration used was 20 mM, which is 200 times higher than that of the tested compounds.

**Figure 10:**
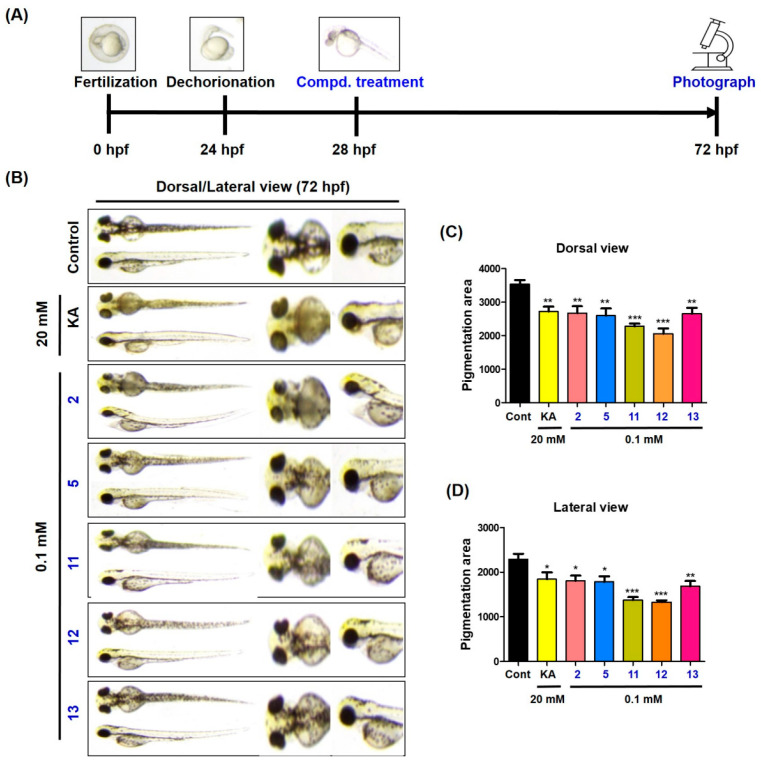
(**A**) Experimental manipulation over h post fertilization (hpf). (**B**) Dorsal and lateral views of zebrafish larvae at 72 hpf. Kojic acid (KA) and compounds **2**, **5**, and **11**–**13** were treated at 20 and 0.1 mM, respectively. Pigmented areas of zebrafish in dorsal (**C**) and lateral (**D**) views. *** *p* < 0.001, ** *p* < 0.01, * *p* < 0.05 vs. untreated control.

Figure 10 depicts the experimental manipulation over time, images of pigmentation, and pigmented areas of the zebrafish larvae at 72 hpf. Kojic acid exhibited a significant depigmentation effect compared with that of the control. All tested compounds also showed depigmentation efficacy similar to that of kojic acid, but at concentrations 200 times lower than that of kojic acid. These results suggest that all compounds tested may be promising anti-melanogenic agents.

### 2.11. Cell Viability of Compounds ***5*** and ***9*** on HaCaT Cells

In addition to melanocytes, keratinocytes are the primary cells present in the epidermis [57]. Therefore, the cytotoxicity of compounds **5** and **9**, which exhibited potent anti-melanogenic effects, was examined in HaCaT (keratinocytes) cells. Each compound was treated at concentrations of 0, 1, 2, 5, 10, and 20 μM for 24 h, and cell viability was measured using the EZ-Cytox solution.

**Figure 11:**
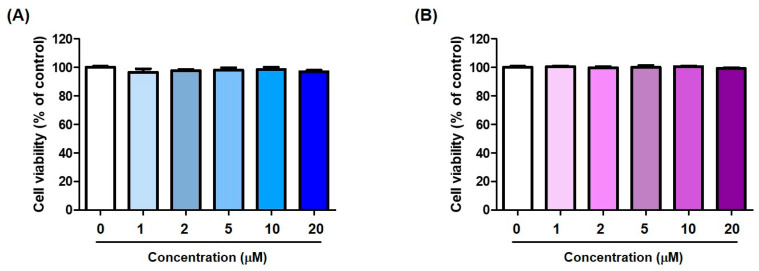
Effects of compounds **5** (**A**) and **9** (**B**) on HaCaT cell viability. Each compound was administered at concentrations of 0, 1, 2, 5, 10, and 20 μM for 24 h.

None of the compounds revealed significant cytotoxicity up to 20 μM, indicating that **5** and **9** may be used to inhibit melanin production at low concentrations without showing cytotoxicity to the epidermis (Figure 11).

## 3. Materials and Methods

### 3.1. Synthesis

#### General Methods

Thin-layer chromatography (TLC) on Merck silica gel 60F_254_ was used to monitor reactions, which were visualized under UV light (254 nm). Chemical solvents and reagents were acquired from SEJIN CI Co. (Seoul, Republic of Korea) and Daejung Chemicals (Siheung-si, Republic of Korea). Nuclear magnetic resonance (NMR) was used to confirm the structures of the synthesized compounds. ^1^H- and ^13^C-NMR data were acquired using a JEOL ECZ400S instrument (JEOL, Ltd. Tokyo, Japan). Chemical shifts and coupling constants were described in the form of *δ* values (parts per million, ppm) and Hz, respectively. The peak-splitting patterns were described as follows: brs = broad singlet, s = singlet, m = multiplet, dd = doublet of doublets, d = doublet, t = triplet, and q = quartet. Using an Agilent Accurate Mass Q-TOF (quadruple time of flight) liquid chromatography mass spectrometer (Agilent, Santa Clara, CA, USA), high-resolution mass spectrometry (HRMS) data were obtained in electrospray ionization-positive (ESI+) mode.

1-(Benzo[*d*][1,3]dioxol-5-yl)thiourea (**14**)

Benzoyl chloride (3.9 mL, 33.63 mmol) was slowly added to an acetone solution (30 mL) of ammonium thiocyanate (3.2 g, 42.04 mmol). After stirring for 20 min at 50 °C, 3,4-(methylenedioxy)aniline (5.0 g, 36.46 mmol) in acetone (35 mL) was added dropwise to the reaction mixture. The reaction mixture was stirred at 50 °C for 2 h before adding excess water. The precipitate generated by adding water was filtered to obtain a filter cake. After stirring the filter cake in 2N–NaOH solution (60 mL) at 100 °C for 1 h, water was added to the reaction mixture. The generated precipitate was filtered and washed with water to obtain compound **14** (3.4 g, 48%) as a solid. Compound **14** was used directly in the next step without further purification.

[1,3]Dioxolo[4′,5′:4,5]benzo[1,2-*d*]thiazol-6-amine (**15**)

To an acetic acid (30 mL) solution of **14** (1.71 g, 8.71 mmol), bromine (0.45 mL, 8.78 mmol) was added dropwise. The reaction mixture was stirred at RT for 40 min. After adding diethyl ether, the generated precipitate was filtered. Water (30 mL) and 25% ammonium hydroxide (15 mL) were added to the filter cake, and the mixture was stirred for 5 min. The remaining solid was filtered and washed with water to obtain compound **15** (1.454 g, 86%).

^1^H NMR (DMSO-*d*_6_, 400 MHz) *δ* 7.23 (s, 1H, 8-H), 6.93 (s, 1H, 4-H), 5.96 (s, 2H, CH_2_); ^13^C NMR (DMSO-*d*_6_, 100 MHz) *δ* 167.2, 147.2, 147.2, 143.6, 121.4, 101.7, 101.6, 99.2.

As an alternative synthetic procedure of **15**, a solution of potassium thiocyanate (3.4 g, 34.99 mmol) in acetic acid (18 mL) was added dropwise to a solution of 3,4-(methylenedioxy)aniline (1.2 g, 8.75 mmol) in acetic acid (10 mL). The reaction mixture was stirred at RT for 30 min. After cooling to 0 °C, a solution of bromine (0.48 mL, 9.37 mmol) in acetic acid (5 mL) was added dropwise. The reaction mixture was stirred at RT for 22 h, and cold water was added. The generated precipitate was filtered, and water was added to a flask containing the filter cake. After neutralization with 25% ammonium hydroxide, the generated precipitate was filtered and washed with water to obtain **15** (1.37 g, 81%) as a solid.

6-Aminobenzo[*d*][1,3]dioxole-5-thiol (**16**)

A solution of **15** (1.0 g, 5.15 mmol) in H_2_O (6.0 mL) and 2-methoxyethanol (6.0 mL) was refluxed with potassium hydroxide (5.88 g, 104.80 mmol) for 2 h. After cooling, the reaction mixture was neutralized with acetic acid, and the resulting precipitate was filtered and washed with water. Ethyl acetate and water were added to the filter cake, the solution was partitioned, and the organic layer was evaporated in vacuo. Water and a small amount of ethyl acetate were added to the flask containing the organic-layer residue, filtered, and washed with a small amount of ethyl acetate to obtain pure compound **16** (480 mg, 55%) as a solid.

^1^H NMR (CDCl_3_, 400 MHz) *δ* 6.70 (s, 1H, 4-H), 6.27 (s, 1H, 7-H), 5.86 (s, 2H, 2-H_2_), 4.13 (brs, 2H, NH_2_); ^13^C NMR (DMSO-*d*_6_, 100 MHz) *δ* 151.0, 145.4, 140.0, 115.3, 109.5, 101.1, 96.8.

General synthetic procedure of compounds **1**–**13**

A solution of **16** (80 mg, 0.47 mmol) and the appropriately substituted benzaldehyde (1.0 equiv.) in *N*,*N*-dimethylformamide (DMF; 1.0 mL) was heated at 80 °C with Na_2_S_2_O_5_ (1.0 equiv.) for 1.5–4.5 h. After cooling, DMF was removed in vacuo to produce a solid. Water was added to the flask containing the solid, and the suspension was filtered and washed with water, if necessary, additionally with MeOH or a small amount of dichloromethane to obtain pure compounds **1**–**13** as solids in a yield of 60–84%.

In the NMR spectra, the proton positions of compounds **1**–**13** are numbered identically to those of 6-phenyl-[1,3]dioxolo[4′,5′:4,5]benzo[1,2-*d*]thiazole to reduce confusion.

4-([1,3]Dioxolo[4′,5′:4,5]benzo[1,2-*d*]thiazol-6-yl)-2-methoxyphenol (**1**)

^1^H NMR (DMSO-*d*_6_, 400 MHz) *δ* 9.72 (brs, 1H, OH), 7.61 (s, 1H, 8-H), 7.53 (s, 1H, 2′-H), 7.51 (s, 1H, 4-H), 7.39 (d, *J* = 8.0 Hz, 1H, 6′-H), 6.91 (d, *J* = 8.0 Hz, 1H, 5′-H), 6.13 (s, 2H, 2-H_2_), 3.87 (s, 3H, OCH_3_); ^13^C NMR (DMSO-*d*_6_, 100 MHz) *δ* 166.3, 150.0, 149.1, 148.6, 148.2, 146.7, 127.7, 125.2, 121.0, 116.4, 110.1, 102.5, 102.3, 101.4, 56.2; HRMS (ESI+) *m*/*z* C_15_H_12_NO_4_S (M + H)^+^ calcd 302.0482, obsd 302.0489; yield, 63%.

4-([1,3]Dioxolo[4′,5′:4,5]benzo[1,2-*d*]thiazol-6-yl)-2-ethoxyphenol (**2**)

^1^H NMR (DMSO-*d*_6_, 400 MHz) *δ* 9.64 (s, 1H, OH), 7.60 (s, 1H, 8-H), 7.51 (d, *J* = 2.0 Hz, 1H, 2′-H), 7.50 (s, 1H, 4-H), 7.39 (dd, *J* = 8.0, 2.0 Hz, 1H, 6′-H), 6.91 (d, *J* = 8.0 Hz, 1H, 5′-H), 6.12 (s, 2H, 2-H_2_), 4.12 (q, *J* = 6.8 Hz, 2H, OC*H_2_*CH_3_), 1.38 (t, *J* = 6.8 Hz, 3H, OCH_2_C*H_3_*); ^13^C NMR (DMSO-*d*_6_, 100 MHz) *δ* 166.3, 150.2, 149.1, 148.1, 147.7, 146.7, 127.6, 125.2, 121.0, 116.5, 111.4, 102.5, 102.3, 101.4, 64.5, 15.2; HRMS (ESI+) *m*/*z* C_16_H_14_NO_4_S (M + H)^+^ calcd 316.0638, obsd 316.0636; yield, 66%.

6-(4-Methoxyphenyl)-[1,3]dioxolo[4′,5′:4,5]benzo[1,2-*d*]thiazole (**3**) [58]

^1^H NMR (DMSO-*d*_6_, 400 MHz) *δ* 7.93 (d, *J* = 8.8 Hz, 2H, 2′-H, 6′-H), 7.63 (s, 1H, 8-H), 7.51 (s, 1H, 4-H), 7.08 (d, *J* = 8.8 Hz, 2H, 3′-H, 5′-H), 6.13 (s, 2H, 2-H_2_), 3.84 (s, 3H, OCH_3_); ^13^C NMR (DMSO-*d*_6_, 100 MHz) *δ* 165.8, 161.8, 149.2, 148.2, 146.8, 128.7, 127.8, 126.4, 115.2, 102.5, 102.4, 101.4, 56.0; HRMS (ESI+) *m*/*z* C_15_H_12_NO_3_S (M + H)^+^ calcd 286.0532, obsd 286.0538; yield, 84%.

5-([1,3]Dioxolo[4′,5′:4,5]benzo[1,2-*d*]thiazol-6-yl)-2-methoxyphenol (**4**)

^1^H NMR (DMSO-*d*_6_, 400 MHz) *δ* 9.42 (s, 1H, OH), 7.56 (s, 1H, 8-H), 7.46 (s, 1H, 4-H), 7.41 (d, *J* = 2.0 Hz, 1H, 2′-H), 7.36 (dd, *J* = 8.4, 2.0 Hz, 1H, 6′-H), 6.99 (d, *J* = 8.4 Hz, 1H, 5′-H), 6.09 (s, 2H, 2-H_2_), 3.79 (s, 3H, OCH_3_); ^13^C NMR (DMSO-*d*_6_, 100 MHz) *δ* 166.0, 150.6, 149.2, 148.2, 147.5, 146.8, 127.7, 126.5, 118.9, 113.7, 112.9, 102.5, 102.4, 101.4, 56.2; HRMS (ESI+) *m*/*z* C_15_H_12_NO_4_S (M + H)^+^ calcd 302.0482, obsd 302.0487; yield, 60%.

4-([1,3]Dioxolo[4′,5′:4,5]benzo[1,2-*d*]thiazol-6-yl)phenol (**5**)

^1^H NMR (DMSO-*d*_6_, 400 MHz) *δ* 10.10 (s, 1H, OH), 7.83 (d, *J* = 8.8 Hz, 2H, 2′-H, 6′-H), 7.60 (s, 1H, 8-H), 7.48 (s, 1H, 4-H), 6.90 (d, *J* = 8.8 Hz, 2H, 3′-H, 5′-H), 6.12 (s, 2H, 2-H_2_); ^13^C NMR (DMSO-*d*_6_, 100 MHz) *δ* 166.3, 160.5, 149.2, 148.1, 146.7, 128.9, 127.6, 124.9, 116.5, 102.4, 102.3, 101.4; HRMS (ESI+) *m*/*z* C_14_H_10_NO_3_S (M + H)^+^ calcd 272.0376, obsd 272.0378; yield, 77%.

4-([1,3]Dioxolo[4′,5′:4,5]benzo[1,2-*d*]thiazol-6-yl)-2-bromophenol (**6**)

^1^H NMR (DMSO-*d*_6_, 400 MHz) *δ* 10.97 (s, 1H, OH), 8.08 (d, *J* = 2.0 Hz, 1H, 2′-H), 7.80 (dd, *J* = 8.4, 2.0 Hz, 1H, 6′-H), 7.62 (s, 1H, 8-H), 7.50 (s, 1H, 4-H), 7.08 (d, *J* = 8.4 Hz, 1H, 5′-H), 6.13 (s, 2H, 2-H_2_); ^13^C NMR (DMSO-*d*_6_, 100 MHz) *δ* 164.5, 157.0, 149.1, 148.3, 146.9, 131.2, 128.1, 127.9, 126.3, 117.3, 110.6, 102.5, 102.4, 101.4; HRMS (ESI+) *m*/*z* C_14_H_9_BrNO_3_S (M + H)^+^ calcd 349.9481, obsd 349.9483, (M + H + 2)^+^ calcd 351.9461, obsd 351.9472; yield, 82%.

4-([1,3]Dioxolo[4′,5′:4,5]benzo[1,2-*d*]thiazol-6-yl)-2,6-dibromophenol (**7**)

^1^H NMR (DMSO-*d*_6_, 400 MHz) *δ* 10.63 (brs, 1H, OH), 8.07 (s, 2H, 2′-H, 6′-H), 7.64 (s, 1H, 8-H), 7.51 (s, 1H, 4-H), 6.14 (s, 2H, 2-H_2_); ^13^C NMR (DMSO-*d*_6_, 100 MHz) *δ* 162.7, 153.4, 148.9, 148.5, 147.3, 130.6, 128.3, 127.9, 112.9, 102.6, 102.5, 101.4; HRMS (ESI+) *m*/*z* C_14_H_8_Br_2_NO_3_S (M + H)^+^ calcd 427.8586, obsd 427.8586, (M + H + 2)^+^ calcd 429.8566, obsd 429.8566, (M + H + 4)^+^ calcd 431.8546, obsd 431.8547; yield, 70%.

4-([1,3]Dioxolo[4′,5′:4,5]benzo[1,2-*d*]thiazol-6-yl)benzene-1,2-diol (**8**)

^1^H NMR (DMSO-*d*_6_, 400 MHz) *δ* 9.57 (s, 1H, OH), 9.39 (s, 1H, OH), 7.59 (s, 1H, 8-H), 7.48 (s, 1H, 4-H), 7.43 (d, *J* = 2.0 Hz, 1H, 2′-H), 7.29 (dd, *J* = 8.0, 2.0 Hz, 1H, 6′-H), 6.85 (d, *J* = 8.0 Hz, 1H, 5′-H), 6.12 (s, 2H, 2-H_2_); ^13^C NMR (DMSO-*d*_6_, 100 MHz) *δ* 166.4, 149.2, 148.9, 148.1, 146.6, 146.3, 127.5, 125.2, 119.2, 116.6, 114.0, 102.4, 102.3, 101.4; HRMS (ESI+) *m*/*z* C_14_H_10_NO_4_S (M + H)^+^ calcd 288.0325, obsd 288.0327; yield, 69%.

4-([1,3]Dioxolo[4′,5′:4,5]benzo[1,2-*d*]thiazol-6-yl)benzene-1,3-diol (**9**)

^1^H NMR (DMSO-*d*_6_, 400 MHz) *δ* 11.53 (brs, 1H, OH), 10.05 (s, 1H, OH), 7.81 (d, *J* = 8.8 Hz, 1H, 6′-H), 7.59 (s, 1H, 8-H), 7.48 (s, 1H, 4-H), 6.47–6.40 (m, 2H, 3′-H, 5′-H), 6.11 (s, 2H, 2-H_2_); ^13^C NMR (DMSO-*d*_6_, 100 MHz) *δ* 165.1, 161.5, 158.1, 148.1, 147.0, 146.4, 129.8, 126.8, 111.0, 108.8, 103.2, 102.3, 101.7, 101.1; HRMS (ESI+) *m*/*z* C_14_H_10_NO_4_S (M + H)^+^ calcd 288.0325, obsd 288.0325; yield, 76%.

4-([1,3]Dioxolo[4′,5′:4,5]benzo[1,2-*d*]thiazol-6-yl)-2,6-dimethoxyphenol (**10**)

^1^H NMR (DMSO-*d*_6_, 400 MHz) *δ* 9.08 (brs, 1H, OH), 7.61 (s, 1H, 8-H), 7.52 (s, 1H, 4-H), 7.21 (s, 2H, 2′-H, 6′-H), 6.13 (s, 2H, 2-H_2_), 3.87 (s, 6H, 2×CH_3_); ^13^C NMR (DMSO-*d*_6_, 100 MHz) *δ* 166.4, 149.1, 148.8, 148.2, 146.7, 139.1, 127.8, 124.0, 104.7, 102.5, 102.4, 101.4, 56.7; HRMS (ESI+) *m*/*z* C_16_H_14_NO_5_S (M + H)^+^ calcd 332.0587, obsd 332.0588; yield, 72%.

6-(3,4-Dimethoxyphenyl)-[1,3]dioxolo[4′,5′:4,5]benzo[1,2-*d*]thiazole (**11**)

^1^H NMR (DMSO-*d*_6_, 400 MHz) *δ* 7.58 (s, 1H, 8-H), 7.56 (d, *J* = 2.0 Hz, 1H, 2′-H), 7.52 (dd, *J* = 8.4, 2.0 Hz, 1H, 6′-H), 7.50 (s, 1H, 4-H), 7.10 (d, *J* = 8.0 Hz, 1H, 5′-H), 6.12 (s, 2H, 2-H_2_), 3.88 (s, 3H, CH_3_), 3.85 (s, 3H, CH_3_); ^13^C NMR (DMSO-*d*_6_, 100 MHz) *δ* 166.0, 152.0, 150.0, 149.3, 148.3, 147.0, 128.0, 126.8, 120.8, 113.1, 110.5, 102.6, 102.4, 101.3, 56.5, 56.5; HRMS (ESI+) *m*/*z* C_16_H_14_NO_4_S (M + H)^+^ calcd 316.0638, obsd 316.0635; yield, 63%.

6-(2,4-Dimethoxyphenyl)-[1,3]dioxolo[4′,5′:4,5]benzo[1,2-*d*]thiazole (**12**)

^1^H NMR (DMSO-*d*_6_, 400 MHz) *δ* 8.27 (d, *J* = 8.8 Hz, 1H, 6′-H), 7.59 (s, 1H, 8-H), 7.48 (s, 1H, 4-H), 6.78 (d, *J* = 2.4 Hz, 1H, 3′-H), 6.72 (dd, *J* = 8.8, 2.4 Hz, 1H, 5′-H), 6.11 (s, 2H, 2-H_2_), 4.03 (s, 3H, CH_3_), 3.86 (s, 3H, CH_3_); ^13^C NMR (DMSO-*d*_6_, 100 MHz) *δ* 162.9, 161.1, 158.4, 148.0, 147.3, 146.4, 129.8, 128.6, 115.1, 107.3, 102.2, 102.0, 100.8, 99.1, 56.6, 56.1; HRMS (ESI+) *m*/*z* C_16_H_14_NO_4_S (M + H)^+^ calcd 316.0638, obsd 316.0641; yield, 81%.

6-(3,4,5-Trimethoxyphenyl)-[1,3]dioxolo[4′,5′:4,5]benzo[1,2-*d*]thiazole (**13**)

^1^H NMR (DMSO-*d*_6_, 400 MHz) *δ* 7.60 (s, 1H, 8-H), 7.52 (s, 1H, 4-H), 7.25 (s, 2H, 2′-H, 6′-H), 6.13 (s, 2H, 2-H_2_), 3.90 (s, 6H, 2×CH_3_), 3.77 (s, 3H, CH_3_); ^13^C NMR (DMSO-*d*_6_, 100 MHz) *δ* 165.8, 154.0, 149.2, 148.4, 147.2, 140.9, 129.2, 128.4, 105.2, 102.7, 102.4, 101.3, 60.8, 56.9; HRMS (ESI+) *m*/*z* C_17_H_16_NO_5_S (M + H)^+^ calcd 346.0744, obsd 346.0746; yield, 75%.

### 3.2. Mushroom Tyrosinase Inhibitory Activity Assay [59,60]

The mushroom tyrosinase inhibitory activity of compounds **1**–**13** was evaluated with l-tyrosine or l-dopa as tyrosinase substrates. As the positive control, kojic acid was utilized. To determine their IC_50_ values, compounds and kojic acid were used at three different concentrations (0.08, 0.4, and 2 μM or 2, 10, and 50 μM with l-dopa; 0.02, 0.08, and 0.4 μM or 2, 10, and 50 μM with l-tyrosine). A substrate mixture (170 μL) composed of sodium phosphate buffer (pH 6.5; 17.2 mM) and l-tyrosine or l-dopa (345 μM), mushroom tyrosinase solution (20 μL; 800 units/mL) (Sigma-Aldrich, St. Louis, MO, USA) dissolved in sodium phosphate buffer, and a compound or kojic acid solution (10 μL) dissolved in dimethyl sulfoxide (DMSO) was added to each well of a 96-well plate. The plate was incubated at 37 °C for 30 min, and absorbance of dopachrome formation was recorded spectrophotometrically at 475 nm using a VersaMax^®^ Elisa (VE) microplate reader (Molecular Devices, San Jose, CA, USA) to determine tyrosinase inhibition activity. A VE microplate reader was used in all subsequent experiments to measure optical density. All experiments were carried out independently three times.

### 3.3. Kinetic Analysis of Mushroom Tyrosinase with Compound ***5*** or ***9*** [61,62]

Kinetic analysis of mushroom tyrosinase was performed by recording the initial dopachrome formation rate in the presence of compounds **5** and **9**. A substrate mixture (170 μL) consisting of sodium phosphate buffer (pH 6.5; 17.2 mM) and l-dopa (0.5, 1, 2, 4, 8, and 16 mM), a compound solution (10 μL) dissolved in DMSO, and mushroom tyrosinase solution (20 μL; 120 units/mL) dissolved in sodium phosphate buffer was added to each well of a 96-well plate. The optical density of dopachrome production in each well was recorded at 475 nm at 5 min intervals for 30 min. Compounds were tested at 80, 40, 20, and 0 μM for **5** and at 2, 1.0, 0.5, and 0 μM for **9**, respectively. Potting the inverse of the substrate concentration vs. the reciprocal of the initial dopachrome production rate generated Lineweaver*–*Burk plots.

### 3.4. In Silico Docking Simulations of Compounds ***5*** and ***9*** Against Mushroom Tyrosinase [63]

The 3D X-ray structure of tyrosinase (*Agaricus bisporus*; PDB ID: 2Y9X) was acquired from the PDB (http://www.rcsb.org, accessed on 18 November 2024) to perform in silico docking simulations of compounds **5** and **9** against mushroom tyrosinase. Kojic acid was used to compare binding affinities. Tropolone binds to the tyrosinase active site as a ligand in the crystal structure of 2Y9X. Each ligand (**5**, **9**, and kojic acid) was subjected to energy minimization using Chem3D Pro 12.0 to obtain the most stable conformation. After removing the tropolone bound to the active site, each ligand was docked with the 3D tyrosinase structure using AutoDock Vina (ver. 1.2.0). The binding energy of each ligand was determined using Chimera 1.17.3 (https://www.sussex.ac.uk/its/services/software/list?filter=c&id=465, accessed on 22 November 2024) and AutoDock Vina. Possible chemical interactions between each ligand and tyrosinase were acquired from LigandScout 4.4.8 (http://www.inteligand.com/ligandscout/download.shtml, accessed on 22 November 2024).

### 3.5. Cell Culture

HaCaT human keratinocytes and B16F10 murine cells were cultured at 37 °C in a humidified environment with 5% CO_2_ and a solution comprising 1% penicillin–streptomycin solution (100×), Dulbecco’s modified Eagle’s medium (Welgene, Gyeongsan-si, Republic of Korea), and 10% fetal bovine serum (Gibco, Grand Island, NY, USA).

### 3.6. Cytotoxicity Assay on HaCaT and B16F10 Cells [64,65]

The effect of compounds **5** and **9** on cell viability of HaCaT and B16F10 cells was assessed using an EZ-Cytox solution (EZ-1000^®^, DoGenBio, Seoul, Republic of Korea). HaCaT (5 × 10^3^ cells/well) and B16F10 (1 × 10^3^ cells/well) cells were seeded in a 96-well plate, respectively, and cultured for 24 h under the same conditions as the cell culture. Each well was treated with **5** and **9** at 0, 1, 2, and 5 μM for B16F10 cells and 0, 1, 2, 5, 10, and 20 μM for HaCaT cells. The plates were incubated for 72 h for B16F10 cells and 24 h for HaCaT cells under a CO_2_ incubator. Each well was treated with the EZ-Cytox solution (10 μL), and the plate was left for 2 h under a CO_2_ incubator. Well optical density was measured at 450 nm to determine cytotoxicity.

### 3.7. Cellular Tyrosinase Activity Assay Using B16F10 Cells [66]

To evaluate the effect of compounds **5** and **9** on cellular tyrosinase activity, B16F10 cells (5000 cells/well) were inoculated in a 6-well plate and cultured for 24 h under the same conditions as the cell culture. Kojic acid was used to compare the activities. Each well was treated with kojic acid (positive control) at 5 μM or compounds **5** and **9** at 5, 2, 1, and 0 μM. The plate was incubated in a 5% CO_2_ incubator maintained at 37 °C for 1 h. Each well was treated with stimulators (1 μM α-MSH [Alomone Labs^TM^, Jerusalem, Israel] and 200 μM IBMX [Thermo Fisher, Rockford, IL, USA]) and incubated in a CO_2_ incubator for 72 h. The cells were rinsed twice with PBS (pH 7.4) and treated with 100 μL lysis buffer solution consisting of Triton X-100 (1%; 5 μL), phosphate buffer (50 mM; pH 6.5; 90 μL), and phenylmethanesulfonyl fluoride (2 mM; 5 μL). The lysates were placed for 30 min at −80 °C. After centrifuging at 10,000 *g* and 4 °C for 10 min, an aliquot (80 μL) of the supernatants was transferred to each well of a 96-well plate, and 20 μL of l-dopa solution (10 mM) was added to each well. The optical density at 475 nm was recorded every min for 10 min to determine cellular tyrosinase activity.

### 3.8. Cellular Melanin Content Assay Using B16F10 Cells [66]

B16F10 cells were inoculated and cultured in a 6-well plate for 24 h as the cell tyrosinase activity assay, and then treated with kojic acid (5 μM) or compounds **5** and **9** (0, 1, 2, and 5 μM). The plate was incubated in a CO_2_ incubator for 1 h. Each well was treated with stimulators (α-MSH and IBMX; 1 μM and 200 μM, respectively) and incubated in a CO_2_ incubator for 72 h. The incubated cells were rinsed twice with PBS (pH 7.4). 1N–NaOH solution (100 μL) was added to each well and warmed at 60 °C for 1 h. The lysates were transferred to each well of a 96-well plate. The optical density was measured at 405 nm.

### 3.9. In Situ Cellular Tyrosinase Activity Assay Using B16F10 Cells [67,68]

B16F10 (3 × 10^3^ cells/well) cells in a 24-well plate were incubated for 24 h in a 5% CO_2_ incubator maintained at 37 °C. Each well was treated with a test sample (5, 2, and 1 μM for **5** and **9** and 5 μM for kojic acid [positive control]). Stimulators (α-MSH and IBMX; 1 μM and 200 μM, respectively) were exposed to cells 1 h after treatment with the test sample, and the cells were incubated for 72 h in a 5% CO_2_ incubator. The cells were fixed for 40 min using paraformaldehyde solution (4%) (Sigma-Aldrich, St. Louis, MO, USA), washed with PBS (pH 7.4), and permeabilized for 2 min using Triton X-100 (0.1%). After washing twice with PBS, each well was exposed to l-dopa solution (500 μL; 2 mM). After incubation for 2 h, images of the cells stained with excess melanin were obtained using a camera connected to a microscope (Motic, Hong Kong).

### 3.10. Scavenging Assay for ABTS^•+^ [69]

To prepare ABTS^•+^ stock solution, equal amounts of ABTS (14 mM aqueous solution) and K_2_S_2_O_8_ (4.9 mM aqueous solution) were mixed and stored in the dark at 18 °C for 15 h. The prepared ABTS^•+^ solution was diluted in ethanol to adjust the absorbance to 0.7 ± 0.01 at 732 nm. The test samples (Trolox [positive control] and compounds **1**–**13**) were dissolved in a cosolvent of ethanol and DMSO (10:1 [*v*/*v*]). The adjusted ABTS^•+^ solution (90 μL) and test sample (10 μL) were added to each well of a 96-well plate, and the plate was stored in a dark place at 18 °C for 2 min. The optical density was measured at 732 nm every min for 10 min. The test sample solutions were tested at a final concentration of 100 μM. The percentage of radical scavenging activity was calculated as follows: % Radical scavenging activity = 100 × [(Abs_c_ − Abs_t_)/Abs_c_], where Abs_t_ and Abs_c_ are optical densities of the test sample and control, respectively.

### 3.11. Scavenging Assay for DPPH Radicals [70,71]

An aliquot (20 μL) of test sample (compounds **1**–**13** and ascorbic acid [positive control]; 5 mM) in DMSO and 180 μL DPPH solution (0.2 mM) in methanol were mixed in each well of a 96-well plate. The mixed solution was kept in the dark at 18 °C for 30 min. A VE microplate reader was used to measure the optical density of each well at 517 nm.

### 3.12. In Vivo Pigmentation Assay Using Zebrafish Embryos [72,73]

Zebrafish (*Danio rerio*) embryos were kindly provided by the Zebrafish Disease Modeling Center at Chungnam National University, Daejeon, Republic of Korea. The embryos were placed in a culture dish containing an E3-MB solution and an E3 solution with 0.001% methylene blue, and incubated at 28 °C until use. The E3 solution was prepared by dissolving 6.4 mg potassium chloride, 146.1 mg sodium chloride, 19.9 mg magnesium sulfate, and 18.3 mg calcium chloride in 500 mL distilled water. At 20 h post fertilization (hpf), zebrafish embryos were dechorionated using Pronase^®^ (Sigma-Aldrich Co., St. Louis, MO, USA). Each compound at 100 μM was exposed to five zebrafish embryos for a preliminary experiment (*n* = 1) or six zebrafish embryos for the main experiment (*n* = 3) at 24 hpf. Pigmentation of zebrafish larvae was imaged using the SMZ745T microscope (Nikon, Tokyo, Japan), and the pigmented areas were measured in dorsal and lateral views at 72 hpf using an ATTO CS analyzer (ATTO, Tokyo, Japan). Kojic acid (20 mM) was used as a standard.

### 3.13. Statistical Analysis

GraphPad Prism 5 (La Jolla, CA, USA) was used for analyzing the significance between groups. All results were expressed as mean ± standard errors of the mean (SEM). Significance set at *p* < 0.05 was determined via a one-way analysis of variance followed by the Newman–Keuls test. All experiments were performed independently at least in triplicate.

## 4. Conclusions

Thirteen 6-(substituted phenyl)-[1,3]dioxolo[4′,5′:4,5]benzo[1,2-*d*]thiazole compounds, **1**–**13**, were synthesized as potential tyrosinase inhibitors. Compound **9** potently inhibited mushroom tyrosinase activity, with IC_50_ values of 0.74 and 0.24 μM in the presence of l-dopa and l-tyrosine, respectively. Compounds **5** and **9** exhibited more potent mushroom tyrosinase inhibitory activity than that of kojic acid and were identified as competitive tyrosinase inhibitors based on the kinetic analysis. Additionally, these compounds inhibited cellular tyrosinase activity and melanin production in B16F10 cells. Furthermore, five compounds significantly reduced pigmentation in zebrafish larvae, achieving effects similar to those of kojic acid, even at 200-fold-lower concentrations.

## Figures and Tables

**Table 1:** IC_50_ values and substitution pattern on the phenyl ring of target compounds **1**–**13** against mushroom tyrosinase.

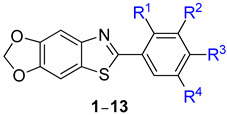						
Cpd	R^1^	R^2^	R^3^	R^4^	IC_50_ (μM, Mean ± SEM) ^a^ /Inhibition (%, Mean ± SEM) ^b^ at 50 μM	
					l-Dopa	l-Tyrosine
**1**	H	OCH_3_	OH	H	ND ^c^/1.71 ± 0.36	ND/25.90 ± 1.51
**2**	H	OEt	OH	H	49.73 ± 2.77/50.33 ± 2.04	ND/14.90 ± 0.76
**3**	H	H	OCH_3_	H	ND/17.19 ± 3.09	ND/11.05 ± 0.82
**4**	H	OH	OCH_3_	H	40.89 ± 3.57/63.04 ± 5.54	ND/25.52 ± 2.57
**5**	H	H	OH	H	36.12 ± 2.17/63.28 ± 3.14	34.21 ± 0.68/61.39 ± 0.65
**6**	H	Br	OH	H	ND/27.94 ± 0.78	ND/17.0 ± 2.36
**7**	H	Br	OH	Br	ND/29.70 ± 0.39	ND/17.72 ± 1.10
**8**	H	OH	OH	H	ND/18.61 ± 1.38	ND/4.95 ± 0.63
**9**	OH	H	OH	H	0.74 ± 0.03/76.11 ± 0.41 ^d^	0.24 ± 0.00/71.76 ± 0.11 ^e^
**10**	H	OCH_3_	OH	OCH_3_	50.13 ± 8.65/53.20 ± 8.09	ND/0.54 ± 0.16
**11**	H	OCH_3_	OCH_3_	H	ND/19.45 ± 0.35	ND/0.82 ± 0.05
**12**	OCH_3_	H	OCH_3_	H	ND/32.77 ± 1.69	ND/10.63 ± 1.53
**13**	H	OCH_3_	OCH_3_	OCH_3_	ND/4.99 ± 1.93	ND/16.56 ± 3.62
KA ^f^					26.18 ± 1.34/74.09 ± 2.32	15.99 ± 0.72/90.83 ± 0.63

^a^ IC_50_ values were determined with l-dopa or l-tyrosine as tyrosinase substrates. ^b^ When the percent inhibition was less than 50% at 50 μM, it was expressed as percent inhibition at 50 μM. ^c^ ND, not determined. ^d^ Inhibition (%) measured at 2.0 μM. ^e^ Inhibition (%) measured at 0.4 μM. ^f^ Kojic acid, positive material. SEM, standard error of the mean.

## Data Availability

The data presented in this study are available in the article and Supplementary Materials.

## Supplementary Materials

The following supporting information can be downloaded at: https://www.mdpi.com/article/10.3390/molecules30071535/s1, S1. ^1^H NMR spectrum of analog **1**; S2. ^13^C NMR spectrum of analog **1**; S3. HRMS (ESI+) spectrum of analog **1**; S4. ^1^H NMR spectrum of analog **2**; S5. ^13^C NMR spectrum of analog **2**; S6. HRMS (ESI+) spectrum of analog **2**; S7. ^1^H NMR spectrum of analog **3**; S8. ^13^C NMR spectrum of analog **3**; S9. HRMS (ESI+) spectrum of analog **3**; S10. ^1^H NMR spectrum of analog **4**; S11. ^13^C NMR spectrum of analog **4**; S12. HRMS (ESI+) spectrum of analog **4**; S13. ^1^H NMR spectrum of analog **5**; S14. ^13^C NMR spectrum of analog **5**; S15. HRMS (ESI+) spectrum of analog **5**; S16. ^1^H NMR spectrum of analog **6**; S17. ^13^C NMR spectrum of analog **6**; S18. HRMS (ESI+) spectrum of analog **6**; S19. ^1^H NMR spectrum of analog **7**; S20. ^13^C NMR spectrum of analog **7**; S21. HRMS (ESI+) spectrum of analog **7**; S22. ^1^H NMR spectrum of analog **8**; S23. ^13^C NMR spectrum of analog **8**; S24. HRMS (ESI+) spectrum of analog **8**; S25. ^1^H NMR spectrum of analog **9**; S26. ^13^C NMR spectrum of analog **9**; S27. HRMS (ESI+) spectrum of analog **9**; S28. ^1^H NMR spectrum of analog **10**; S29. ^13^C NMR spectrum of analog **10**; S30. HRMS (ESI+) spectrum of analog **10**; S31. ^1^H NMR spectrum of analog **11**; S32. ^13^C NMR spectrum of analog **11**; S33. HRMS (ESI+) spectrum of analog **11**; S34. ^1^H NMR spectrum of analog **12**; S35. ^13^C NMR spectrum of analog **12**; S36. HRMS (ESI+) spectrum of analog **12**; S37. ^1^H NMR spectrum of analog **13**; S38. ^13^C NMR spectrum of analog **13**; S39. HRMS (ESI+) spectrum of analog **13**; S40. ^1^H NMR spectrum of analog **15**; S41. ^13^C NMR spectrum of analog **15**; S42. ^1^H NMR spectrum of analog **16**; S43. ^13^C NMR spectrum of analog **16**; S44. Alignment of the re-docked ligand (green) and co-crystallized ligand (red) with the 2Y9X protein; S45. Depigmentation results of **11** and **13** performed using zebrafish embryos; S46. Graphs used to determine IC_50_ values for compounds **2** and **4** in the presence of L-dopa; S47. Graphs used to determine IC_50_ values for compounds **5** and **9** in the presence of L-dopa; S48. Graphs used to determine IC_50_ values for compounds **10** and kojic acid in the presence of L-dopa; S49. Graphs used to determine IC_50_ values for compounds **5** and **9** in the presence of L-tyrosine; S50. Graphs used to determine an IC_50_ values for kojic acid in the presence of L-tyrosine; S51. Optical density values of compound **5** for 0 min (A) and 10 min (B) in a 96-well plate and values of velocity (V) per minute (min) (C) and 1/V (D) used for Lineweaver–Burk plot; S52. Optical density values of compound **9** for 0 min (A) and 10 min (B) in a 96-well plate and values of velocity (V) per minute (min) (C) and 1/V (D) used for Lineweaver–Burk plot; S53. Values used for Dixon plot of compound **5**; S54. Values used for Dixon plot of compound **9**.
