# Supplementary material for: Design and Synthesis of Novel 6-(Substituted phenyl)-[1,3]dioxolo[4′,5′:4,5]benzo[1,2-d]thiazole Compounds as Tyrosinase Inhibitors: In Vitro and In Vivo Insights
Source: Molecules. 2025 Mar 30;30(7):1535. doi: 10.3390/molecules30071535 (PMC11990524; doi:10.3390/molecules30071535)
Supplement: Supplementary file 1 [file molecules-30-01535-s001.zip › molecules-3514429-supplementary.pdf]

# Supporting Information

# For

## Design and synthesis of novel 6-(substituted phenyl)-[1,3]dioxolo[4′,5′:4,5]benzo[1,2-*d*]thiazole compounds as tyrosinase inhibitors: in vitro and in vivo insights

Hyeon Seo Park [1,†], Hee Jin Jung [1,†], Hye Soo Park [1], Hye Jin Kim [1], Sang Gyun Noh [2], Yujin Park [3], Pusoon Chun [4], Hae Young Chung [2], Hyung Ryong Moon [1,*]

[1]*Department of Manufacturing Pharmacy, College of Pharmacy and Research Institute for Drug Development, Pusan National University, Busan 46241, Republic of Korea*
[2]*Department of Pharmacy, College of Pharmacy and Research Institute for Drug Development, Pusan National University, Busan 46241, Republic of Korea*
[3]*Department of Medicinal Chemistry, New Drug Development Center, Daegu-Gyeongbuk Medical Innovation Foundation, Daegu 41061, Republic of Korea*
[4]*College of Pharmacy and Inje Institute of Pharmaceutical Sciences and Research, Inje University, Gimhae 50834, Republic of Korea*

**Contents**

S1. $^{1}$H NMR spectrum of analog **1** ........ 5

S2. $^{13}$C NMR spectrum of analog **1** ........ 6

S3. HRMS (ESI+) spectrum of analog **1** ........ 7

S4. $^{1}$H NMR spectrum of analog **2** ........ 8

S5. $^{13}$C NMR spectrum of analog **2** ........ 9

S6. HRMS (ESI+) spectrum of analog **2** ........ 10

S7. $^{1}$H NMR spectrum of analog **3** ........ 11

S8. $^{13}$C NMR spectrum of analog **3** ........ 12

S9. HRMS (ESI+) spectrum of analog **3** ........ 13

S10. $^{1}$H NMR spectrum of analog **4** ........ 14

S11. $^{13}$C NMR spectrum of analog **4** ........ 15

S12. HRMS (ESI+) spectrum of analog **4** ........ 16

S13. $^{1}$H NMR spectrum of analog **5** ........ 17

S14. $^{13}$C NMR spectrum of analog **5** ........ 18

S15. HRMS (ESI+) spectrum of analog **5** ........ 19

S16. $^{1}$H NMR spectrum of analog **6** ........ 20

S17. $^{13}$C NMR spectrum of analog **6** ........ 21

S18. HRMS (ESI+) spectrum of analog **6** ........ 22

S19. $^{1}$H NMR spectrum of analog **7** ........ 23

S20. $^{13}$C NMR spectrum of analog **7** ........ 24

S21. HRMS (ESI+) spectrum of analog **7** ........ 25

S22. $^{1}$H NMR spectrum of analog **8** ........ 26

S23. $^{13}$C NMR spectrum of analog **8** ........ 27

S24. HRMS (ESI+) spectrum of analog **8** ........................................................................ 28

S25. $^{1}$H NMR spectrum of analog **9** ........................................................................ 29

S26. $^{13}$C NMR spectrum of analog **9** ........................................................................ 30

S27. HRMS (ESI+) spectrum of analog **9** ........................................................................ 31

S28. $^{1}$H NMR spectrum of analog **10** ........................................................................ 32

S29. $^{13}$C NMR spectrum of analog **10** ........................................................................ 33

S30. HRMS (ESI+) spectrum of analog **10** ........................................................................ 34

S31. $^{1}$H NMR spectrum of analog **11**........................................................................ 35

S32. $^{13}$C NMR spectrum of analog **11** ........................................................................ 36

S33. HRMS (ESI+) spectrum of analog **11**........................................................................ 37

S34. $^{1}$H NMR spectrum of analog **12** ........................................................................ 38

S35. $^{13}$C NMR spectrum of analog **12** ........................................................................ 39

S36. HRMS (ESI+) spectrum of analog **12** ........................................................................ 40

S37. $^{1}$H NMR spectrum of analog **13** ........................................................................ 41

S38. $^{13}$C NMR spectrum of analog **13** ........................................................................ 42

S39. HRMS (ESI+) spectrum of analog **13** ........................................................................ 43

S40. $^{1}$H NMR spectrum of analog **15** ........................................................................ 44

S41. $^{13}$C NMR spectrum of analog **15** ........................................................................ 45

S42. $^{1}$H NMR spectrum of analog **16** ........................................................................ 46

S43. $^{13}$C NMR spectrum of analog **16** ........................................................................ 47

S44. Alignment of the re-docked ligand (green) and co-crystallized ligand (red) with the 2Y9X protein...........................................................................................48

S45. Depigmentation results of **11** and **13** performed using zebrafish embryos..................49

S46. Graphs used to determine $IC_{50}$ values for compounds **2** and **4** in the presence of L-dopa.50

S47. Graphs used to determine $IC_{50}$ values for compounds **5** and **9** in the presence of L-dopa.51

S48. Graphs used to determine $IC_{50}$ values for compounds **10** and kojic acid in the presence of L-dopa…………………………………………………………………………………...52

S49. Graphs used to determine $IC_{50}$ values for compounds **5** and **9** in the presence of L-tyrosine……………………………………………………………………………...…..53

S50. Graphs used to determine an $IC_{50}$ value for kojic acid in the presence of L-tyrosine…...……………………………………………………………………………..54

S51. Optical density values of compound **5** for 0 min (A) and 10 min (B) in a 96-well plate and values of velocity (V) per minute (min) (C) and 1/V (D) used for Lineweaver-Burk plot..…...55

S52. Optical density values of compound **9** for 0 min (A) and 10 min (B) in a 96-well plate and values of velocity (V) per minute (min) (C) and 1/V (D) used for Lineweaver-Burk plot……56

S53. Values used for Dixon plot of compound **5**…………………………………………....57

S54. Values used for Dixon plot of compound **9**……………………………………………58

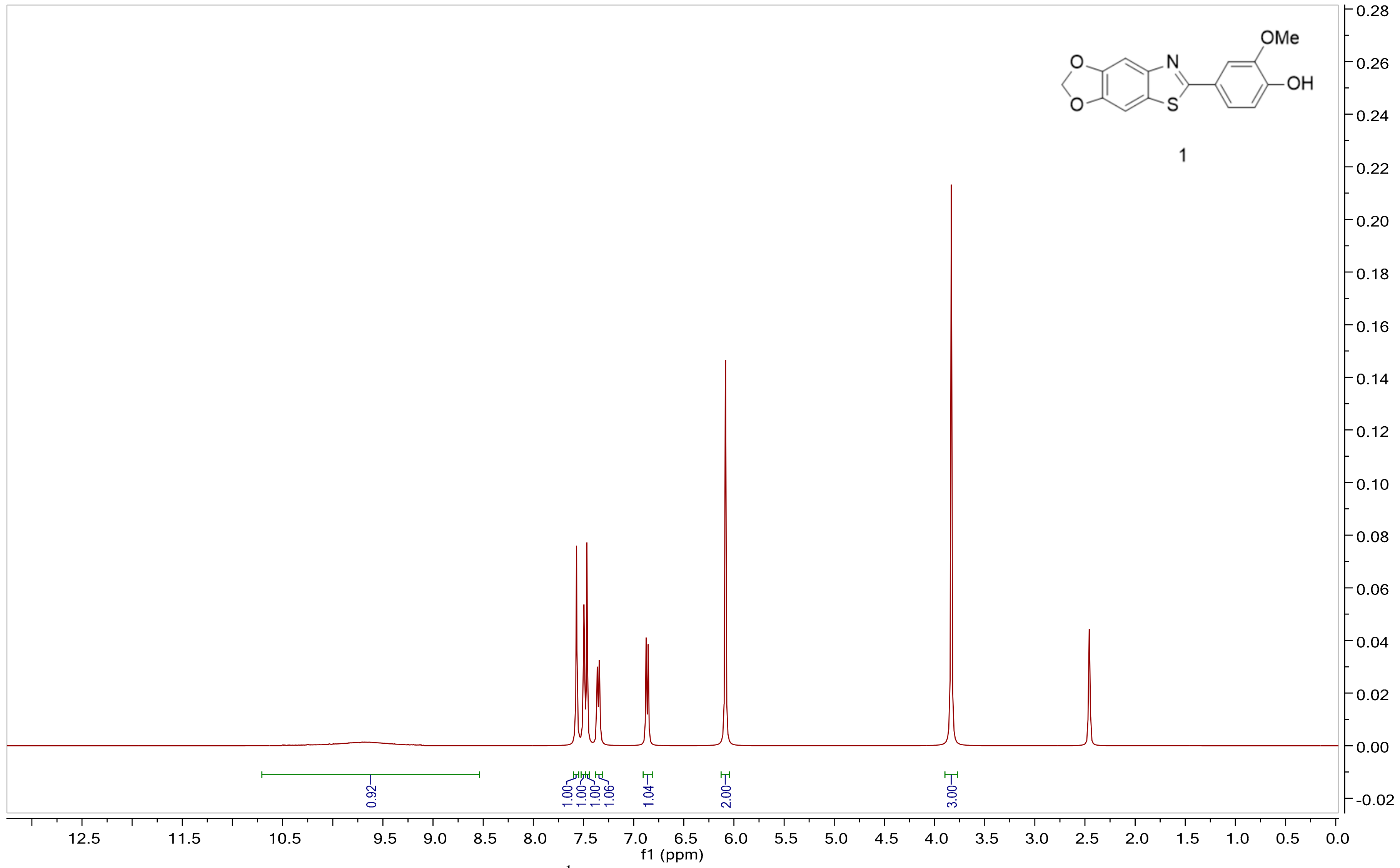


S1. $^{1}$H NMR spectrum of analog **1**

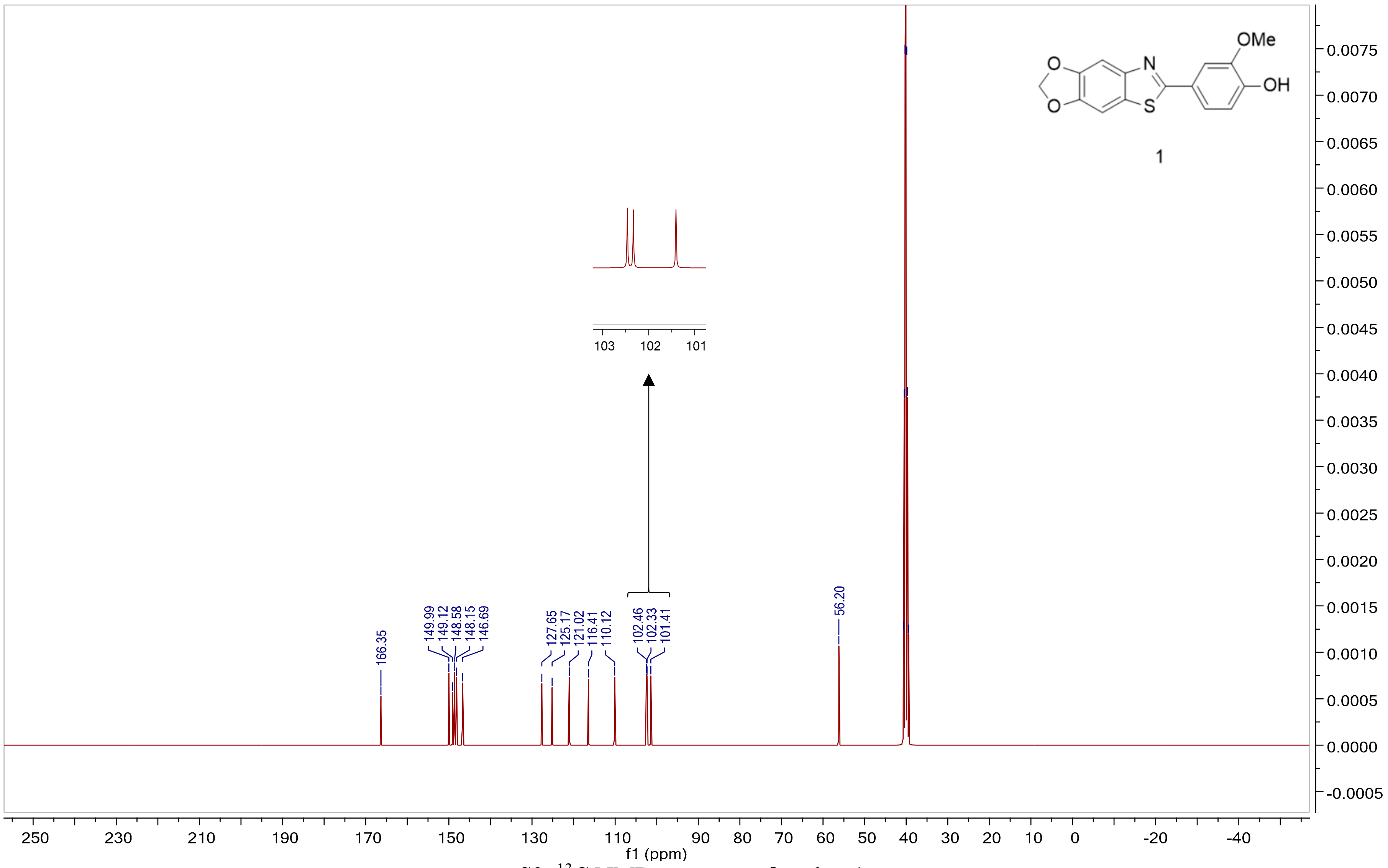


S2. $^{13}$C NMR spectrum of analog **1**

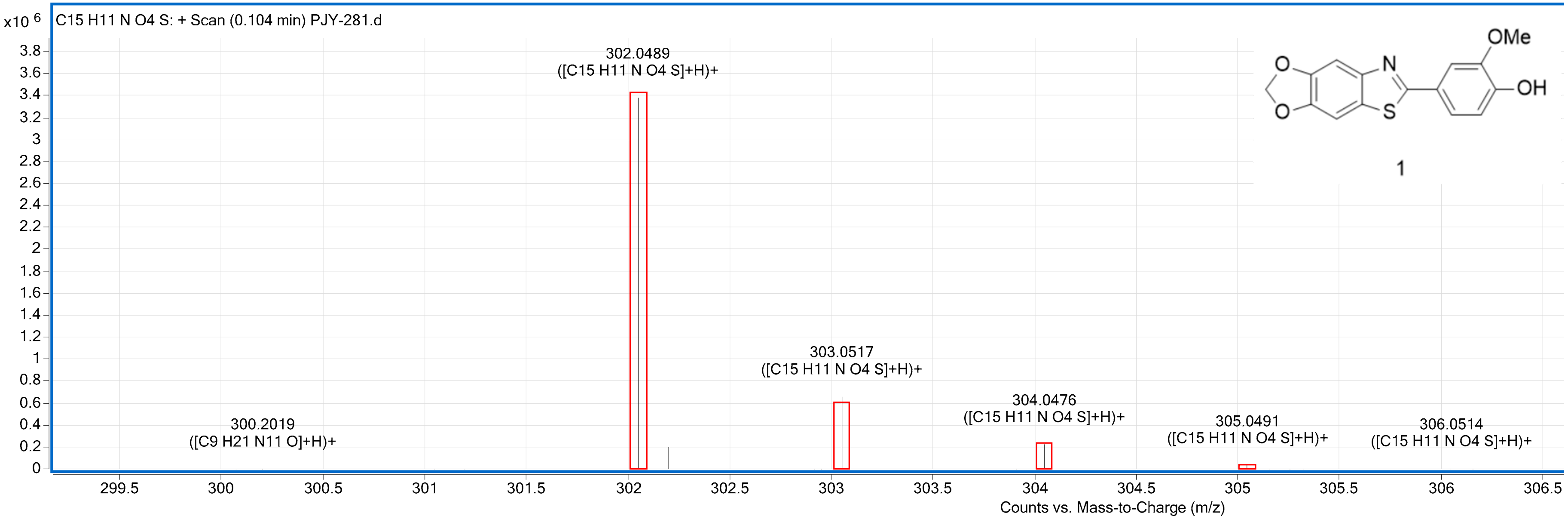


S3. HRMS (ESI+) spectrum of analog **1**

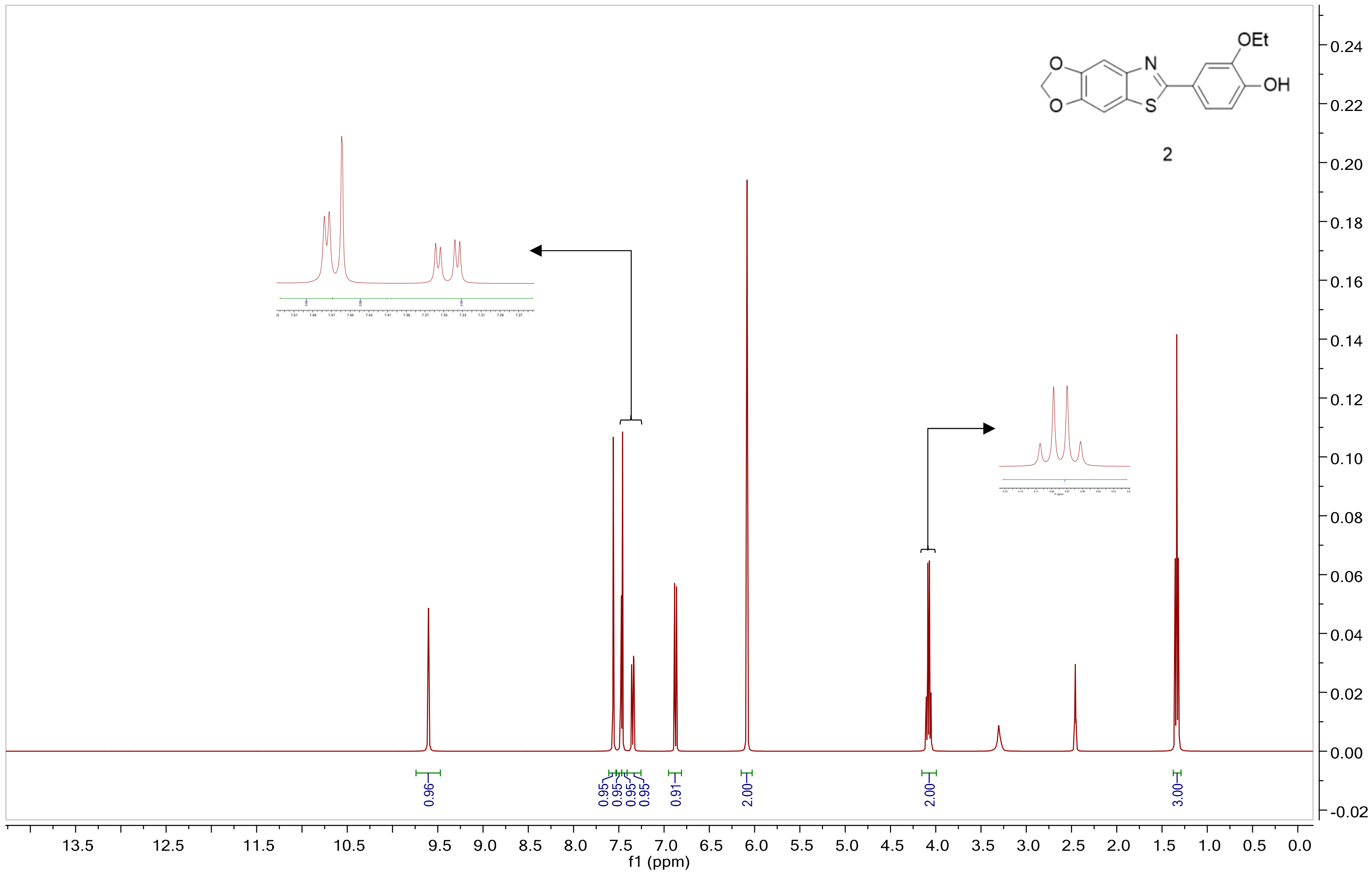


S4. $^{1}$H NMR spectrum of analog **2**

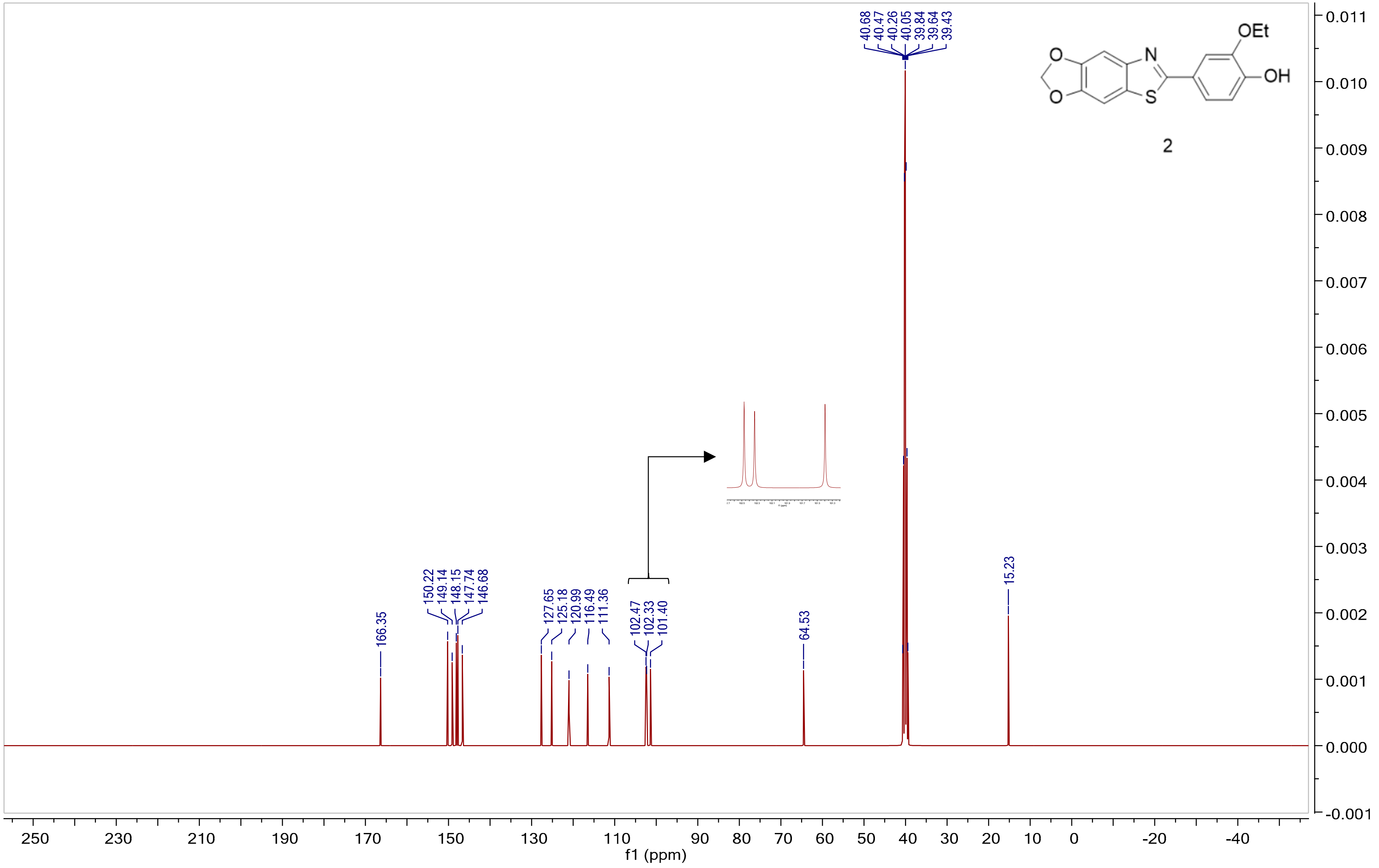


S5. $^{13}C$ NMR spectrum of analog **2**

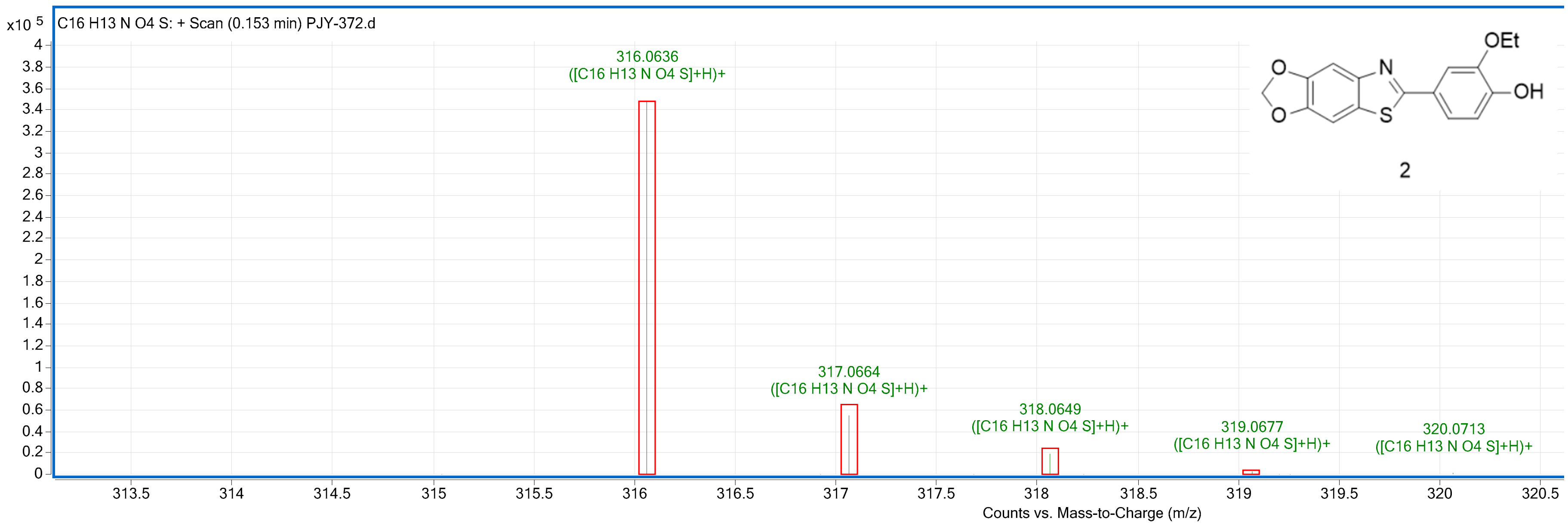


S6. HRMS (ESI+) spectrum of analog **2**

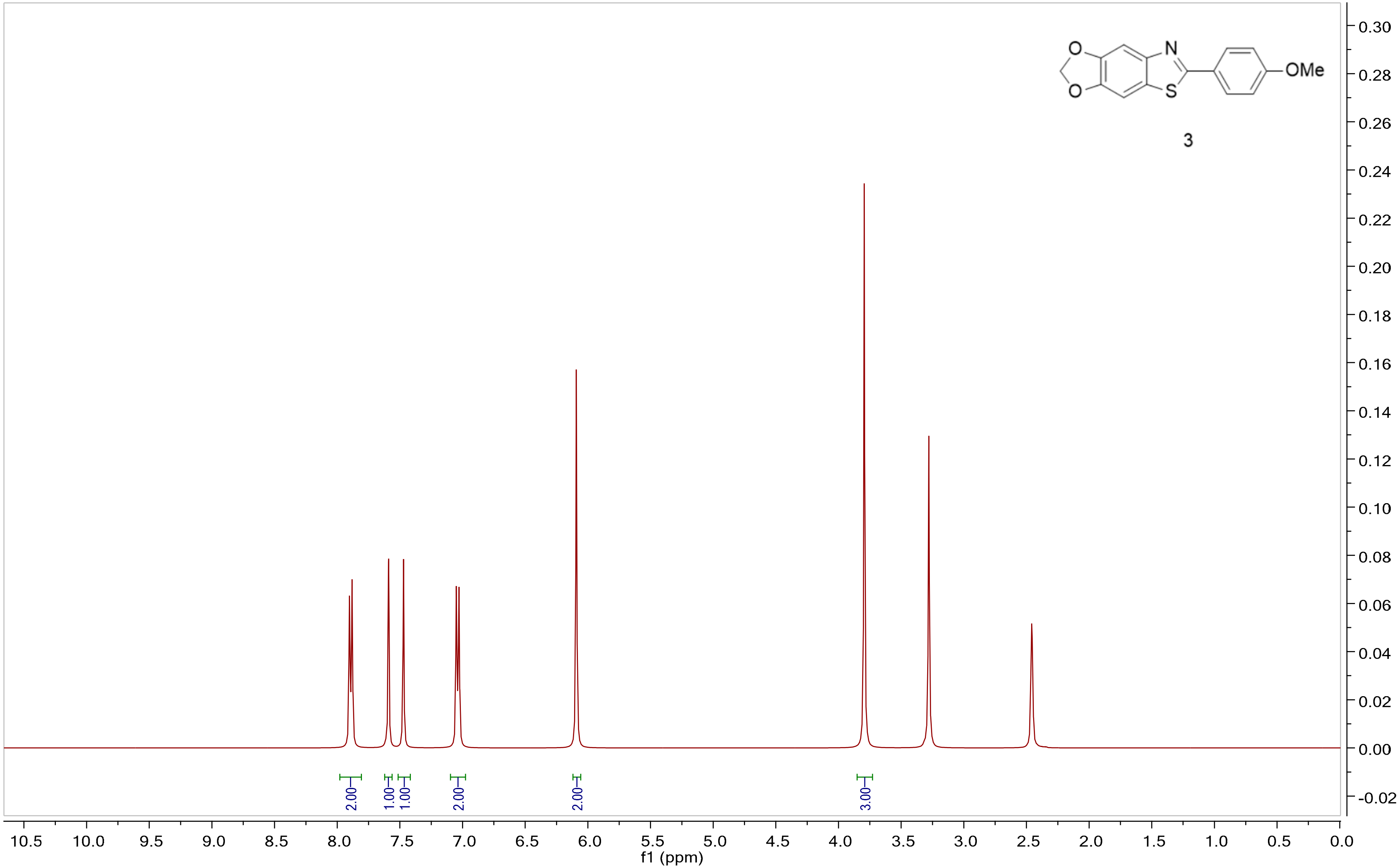


S7. $^{1}$H NMR spectrum of analog **3**

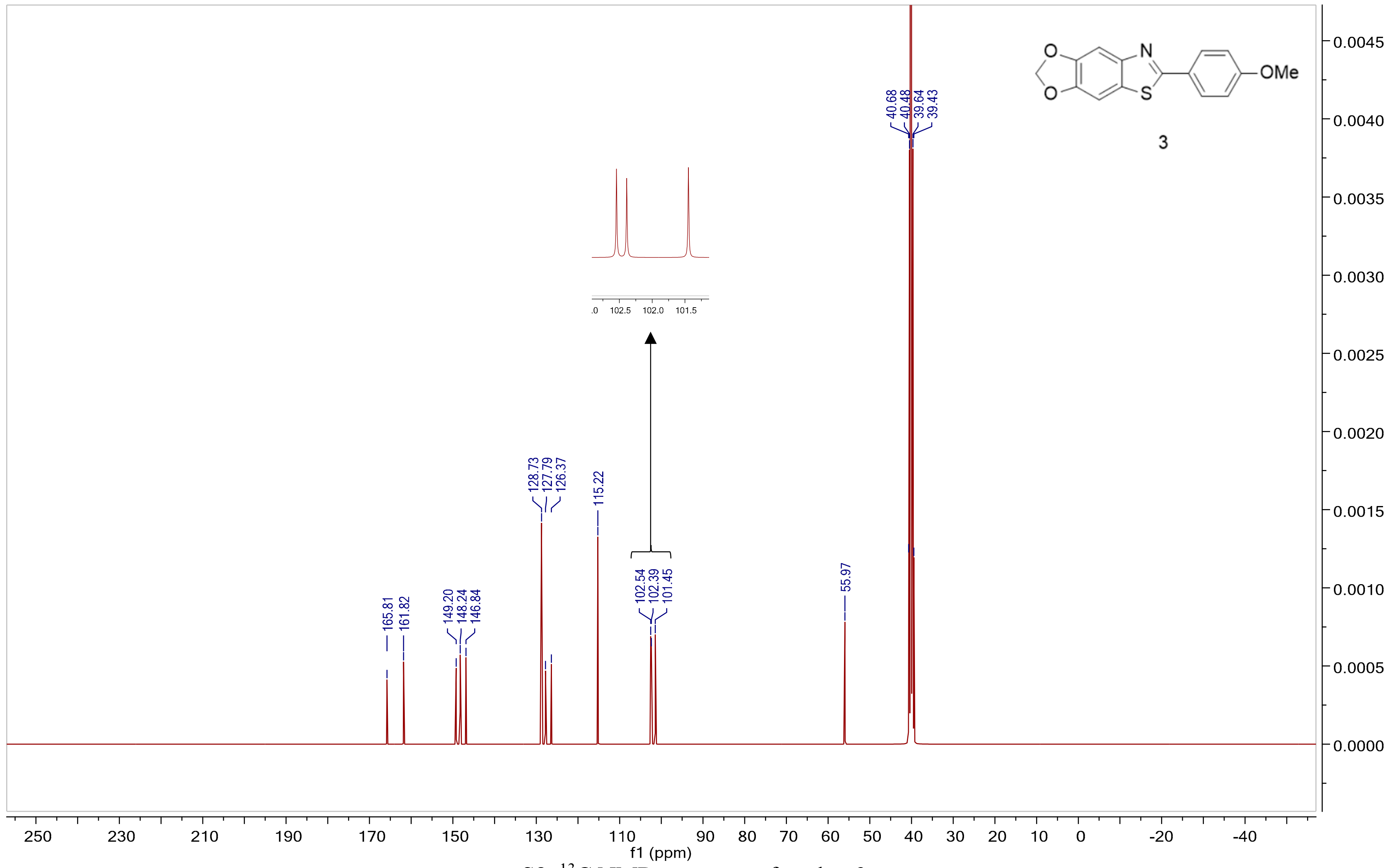


S8. $^{13}C$ NMR spectrum of analog **3**

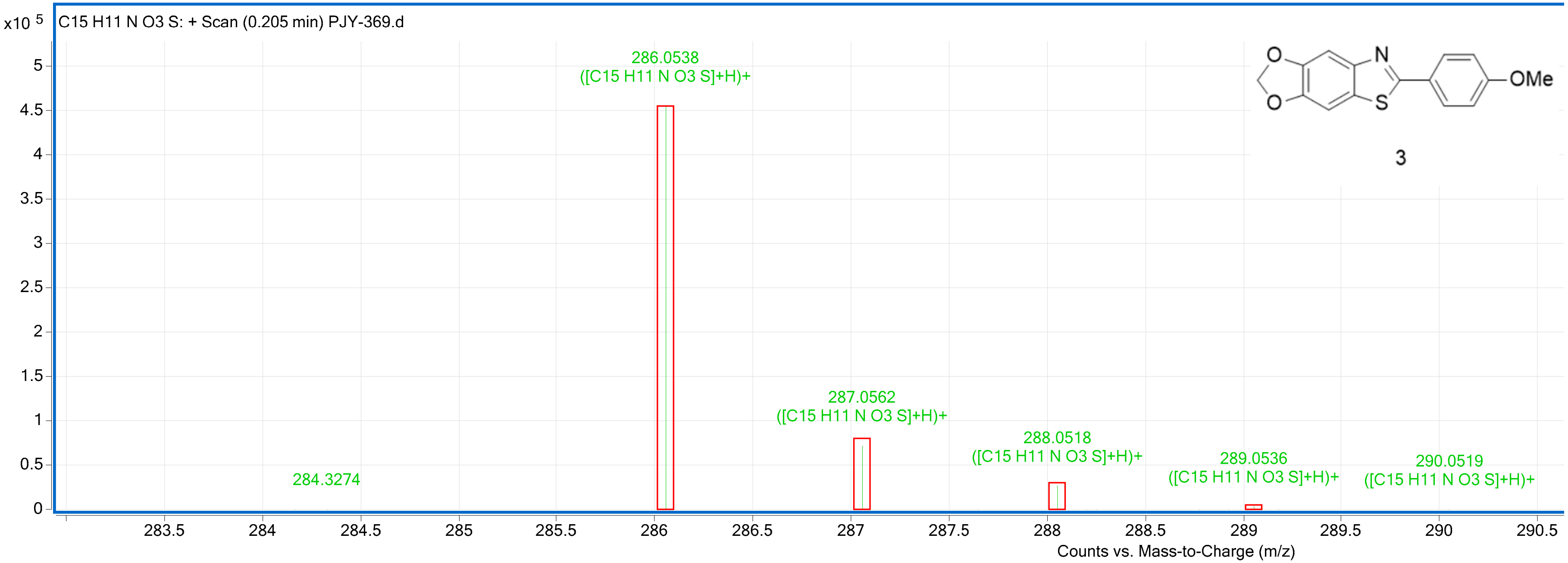


S9. HRMS (ESI+) spectrum of analog **3**

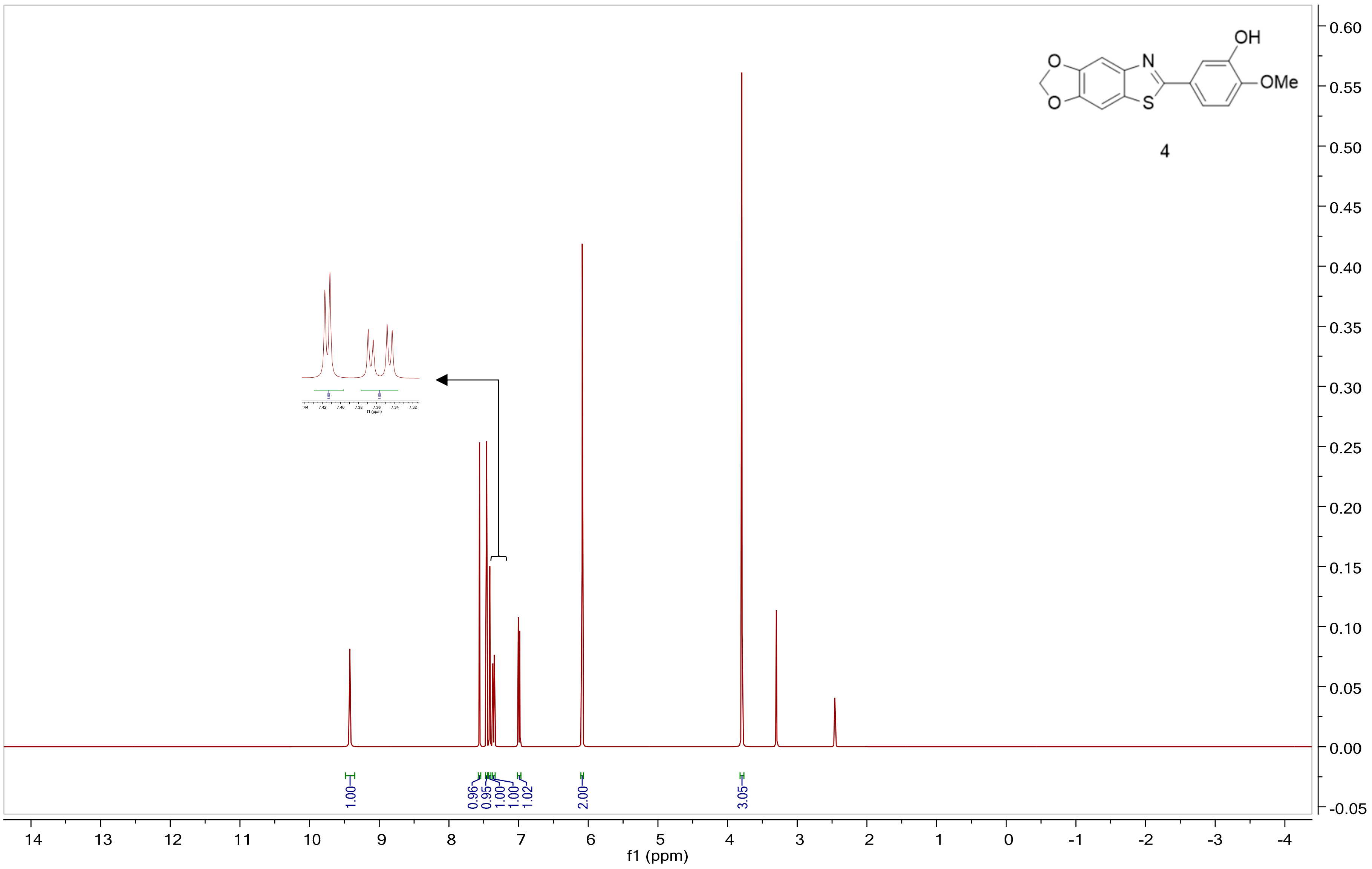


S10. $^{1}H$ NMR spectrum of analog **4**

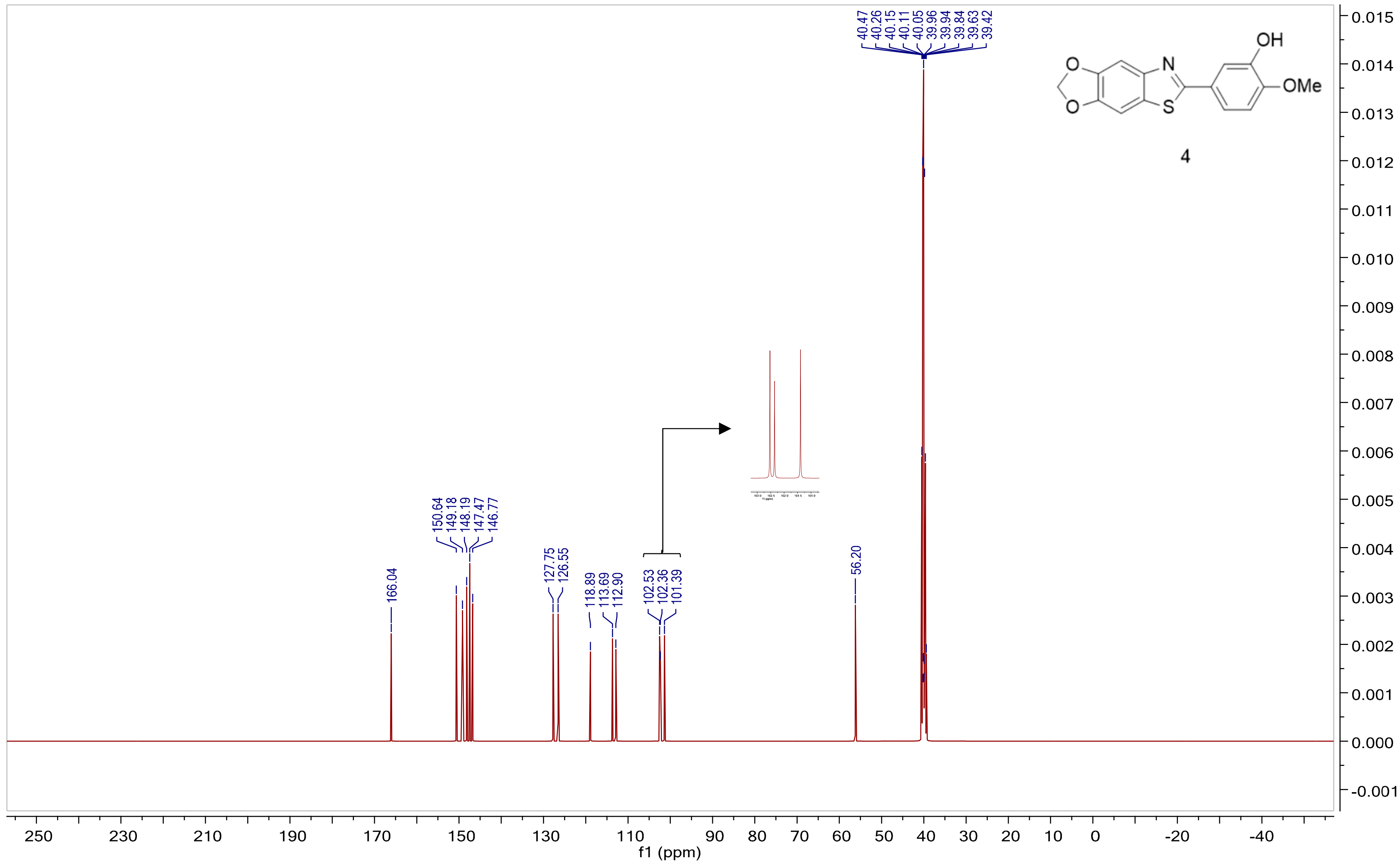


S11. $^{13}$C NMR spectrum of analog **4**

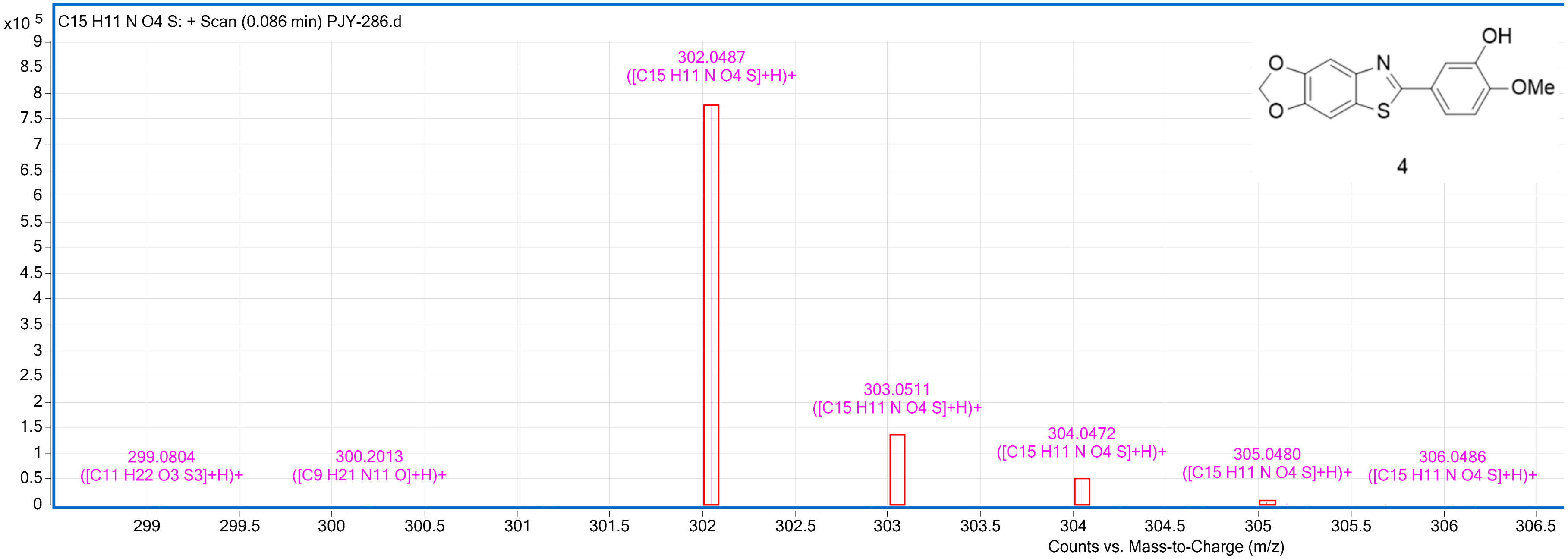


S12. HRMS (ESI+) spectrum of analog **4**

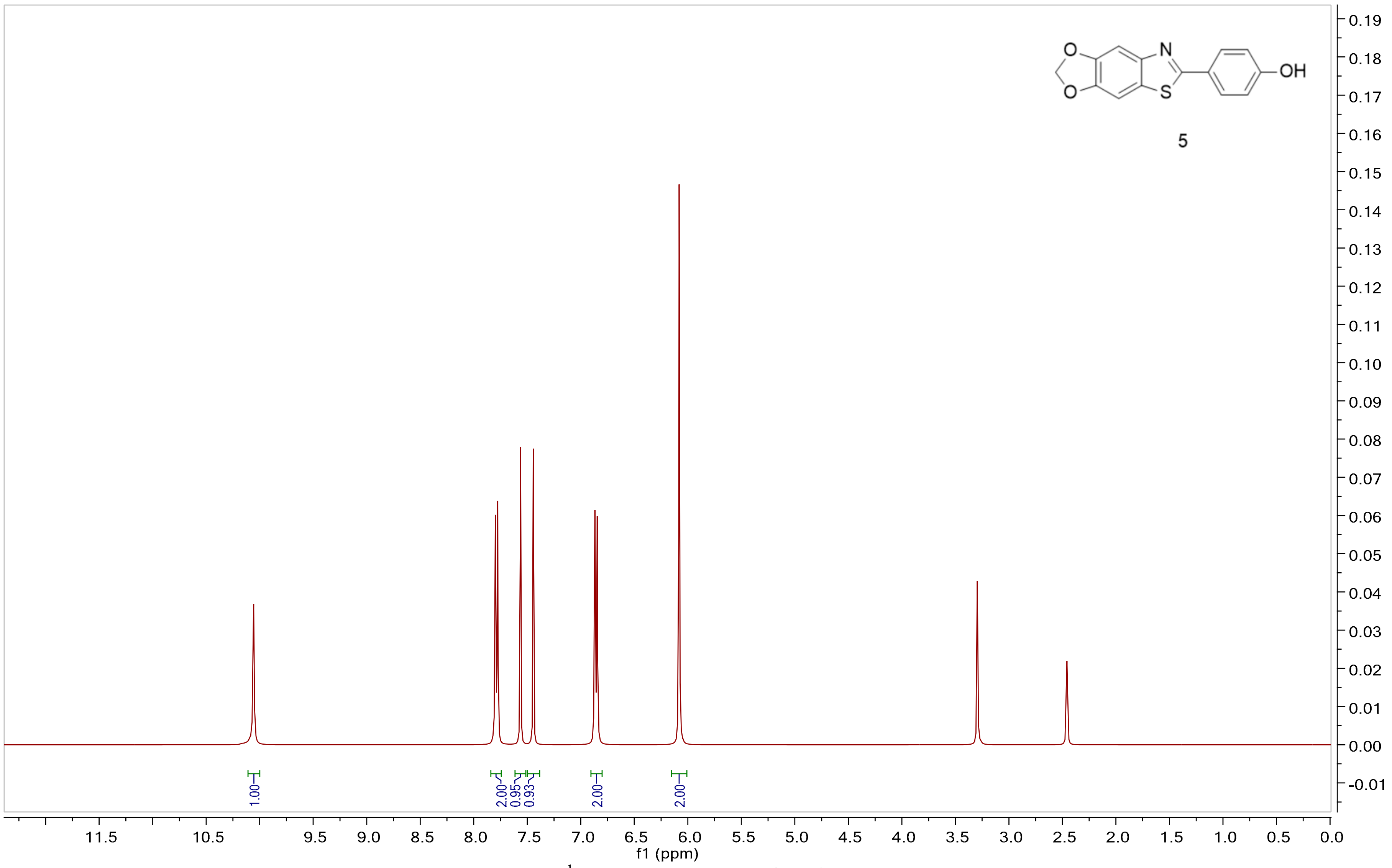


S13. $^{1}$H NMR spectrum of analog **5**

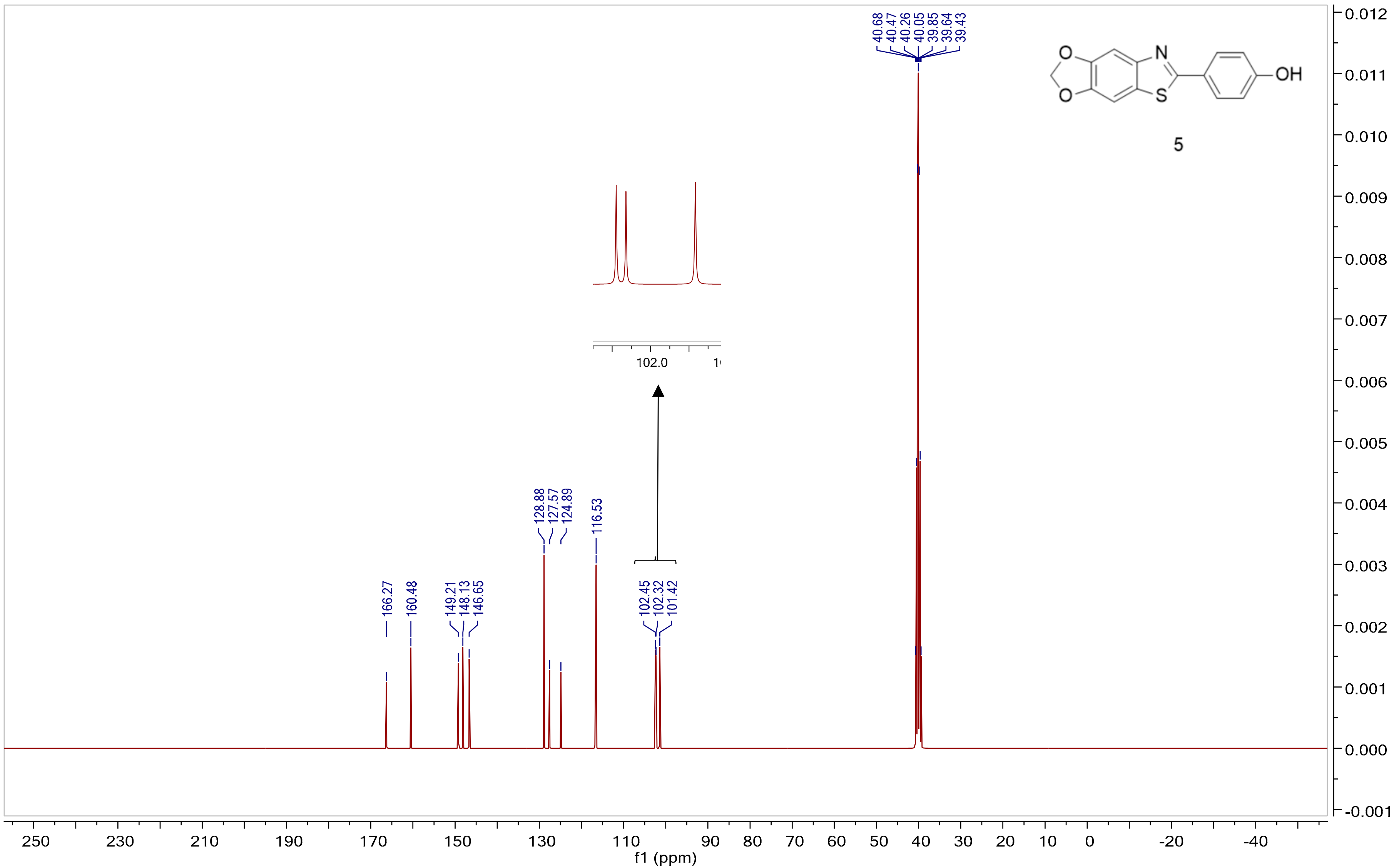


S14. $^{13}$C NMR spectrum of analog **5**

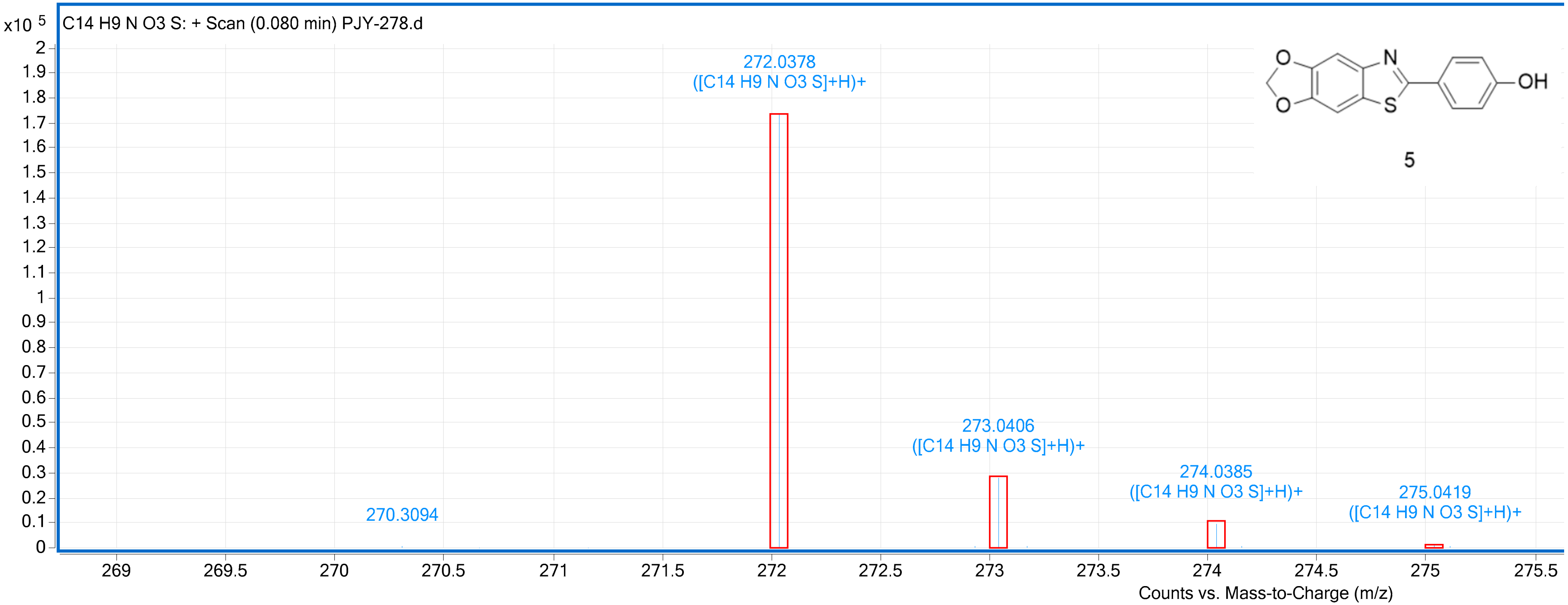


S15. HRMS (ESI+) spectrum of analog **5**

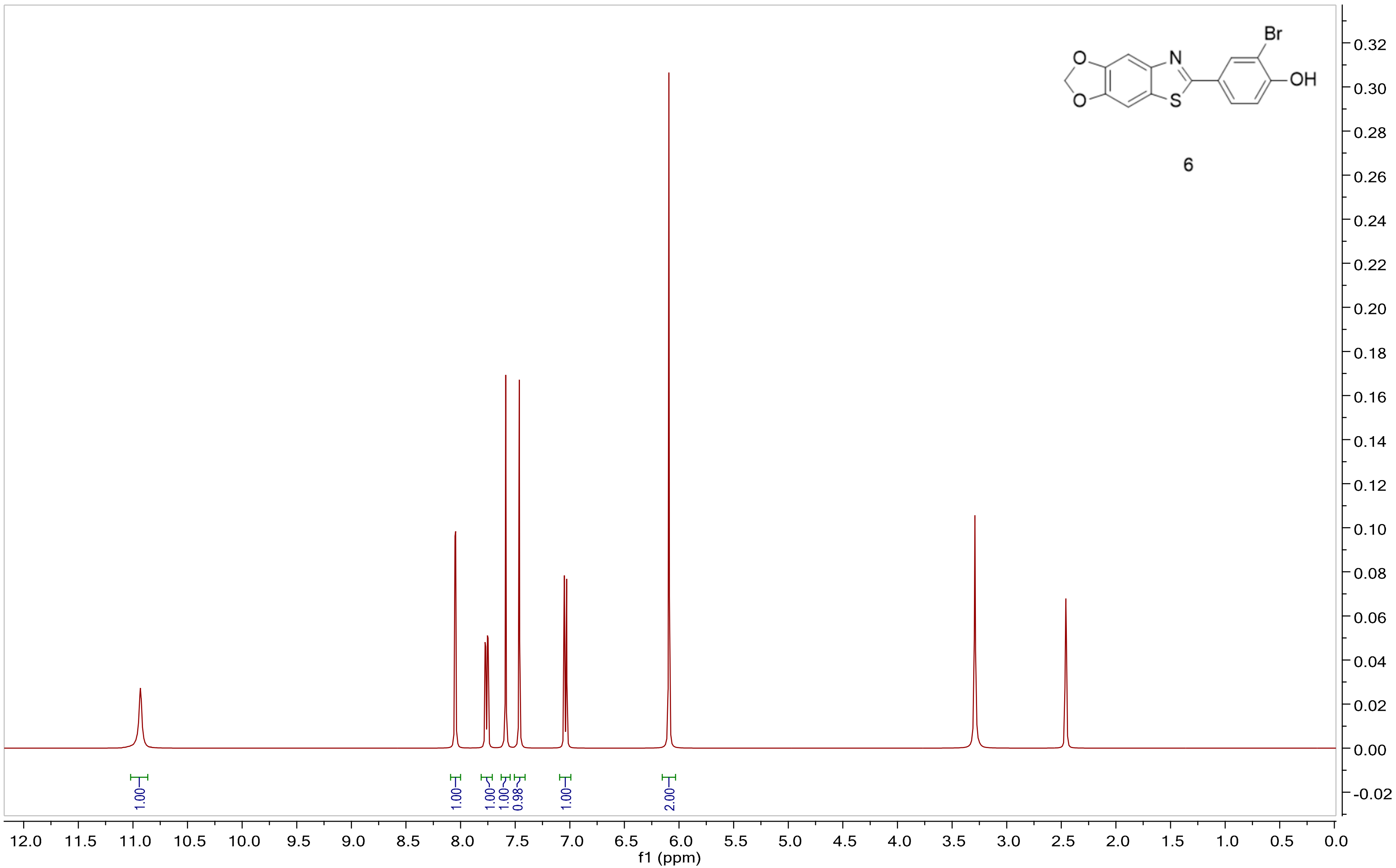


S16. $^{1}$H NMR spectrum of analog **6**

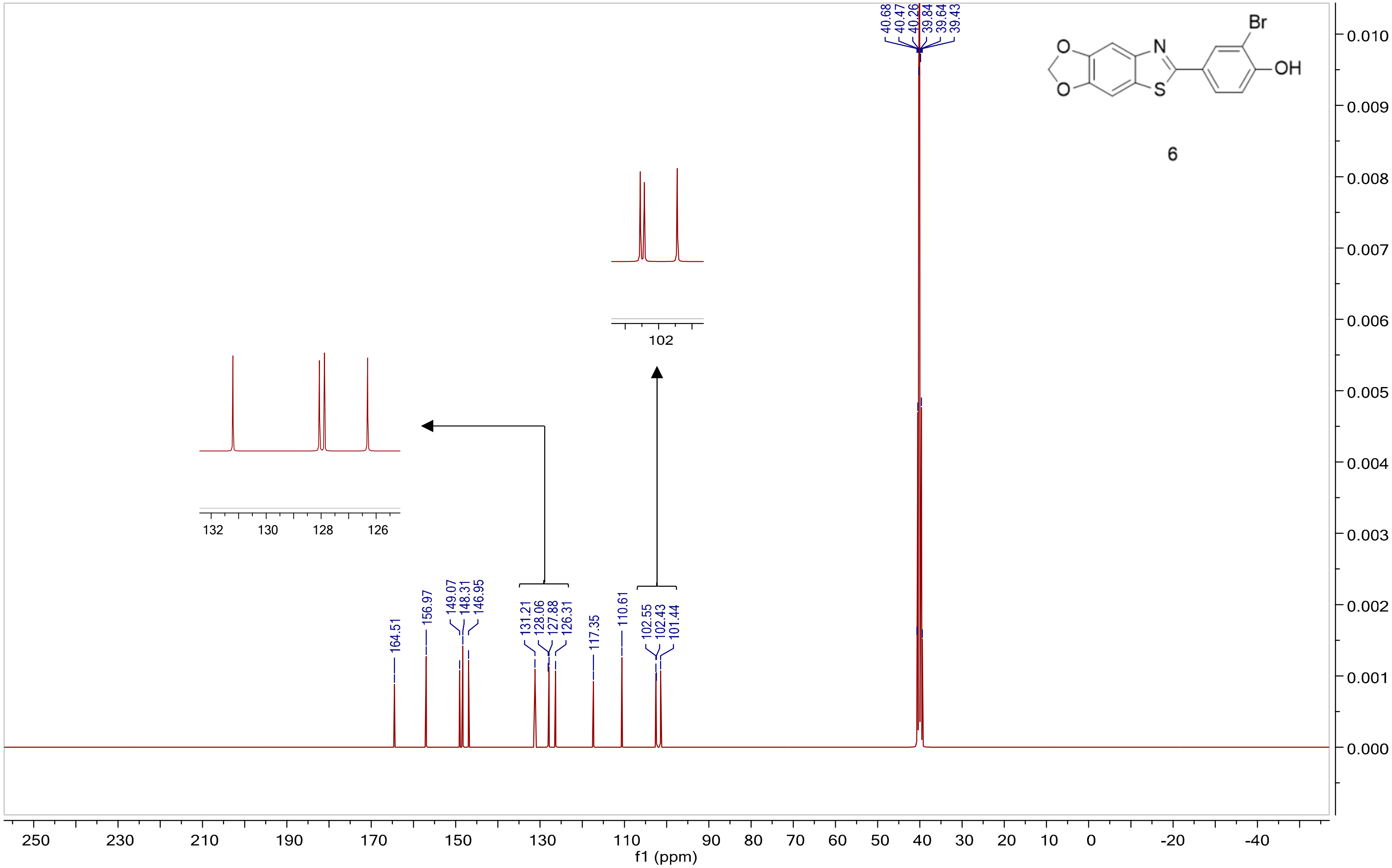


S17. $^{13}$C NMR spectrum of analog **6**

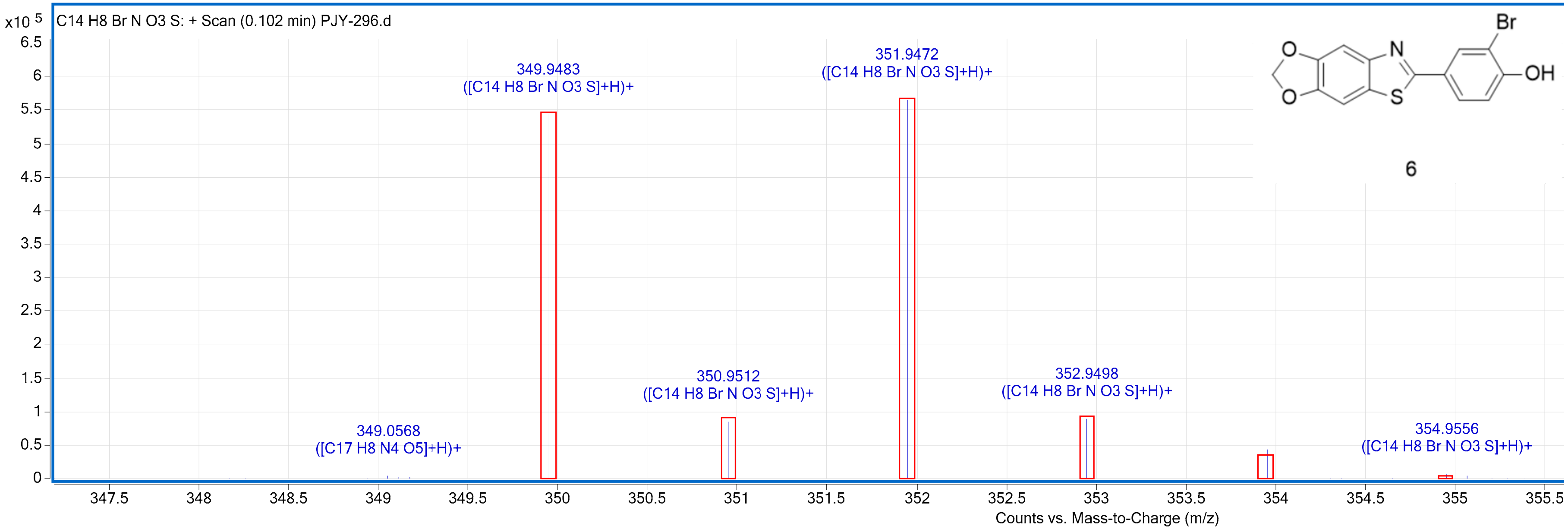


S18. HRMS (ESI+) spectrum of analog **6**

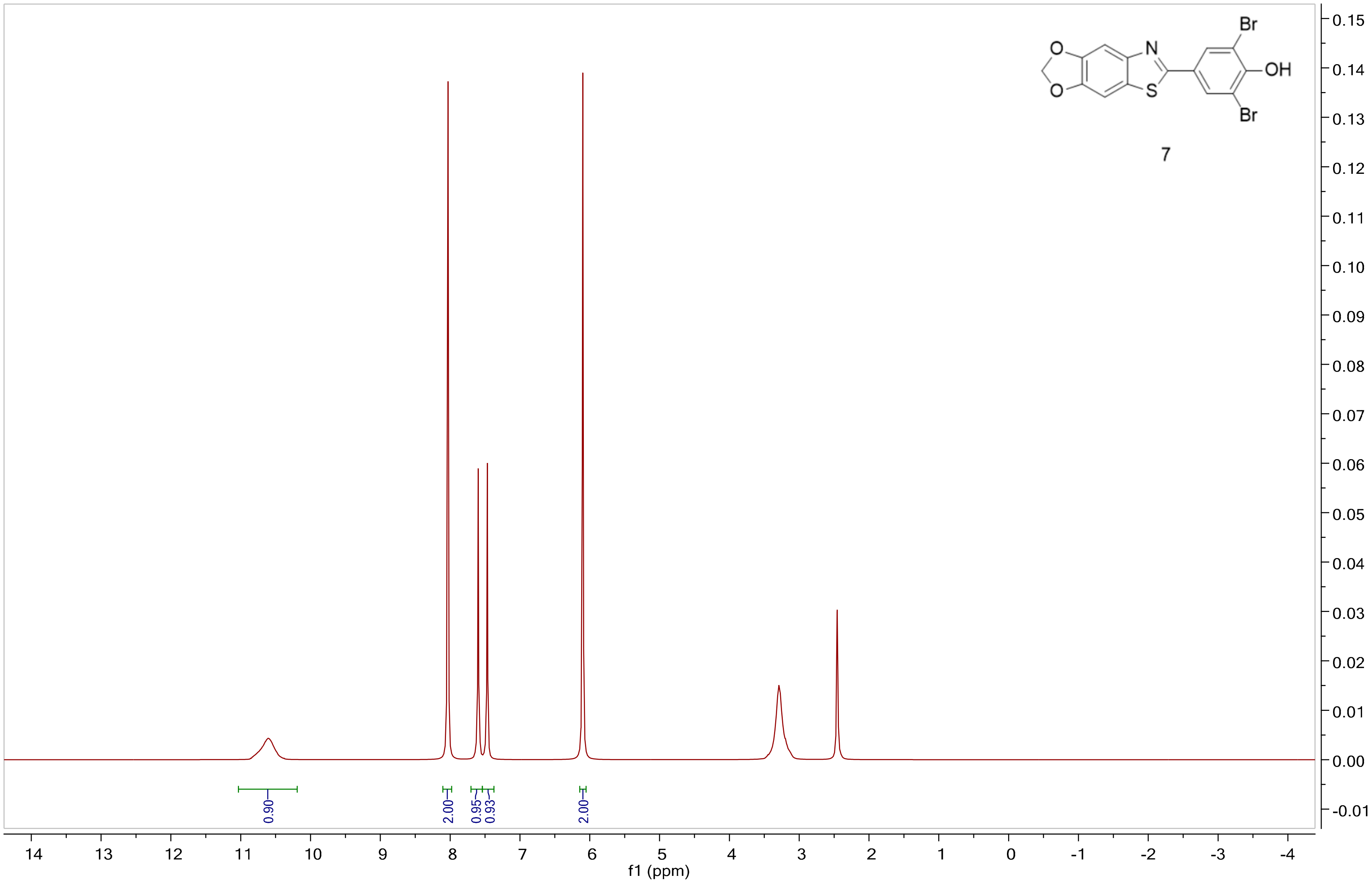


S19. $^{1}$H NMR spectrum of analog **7**

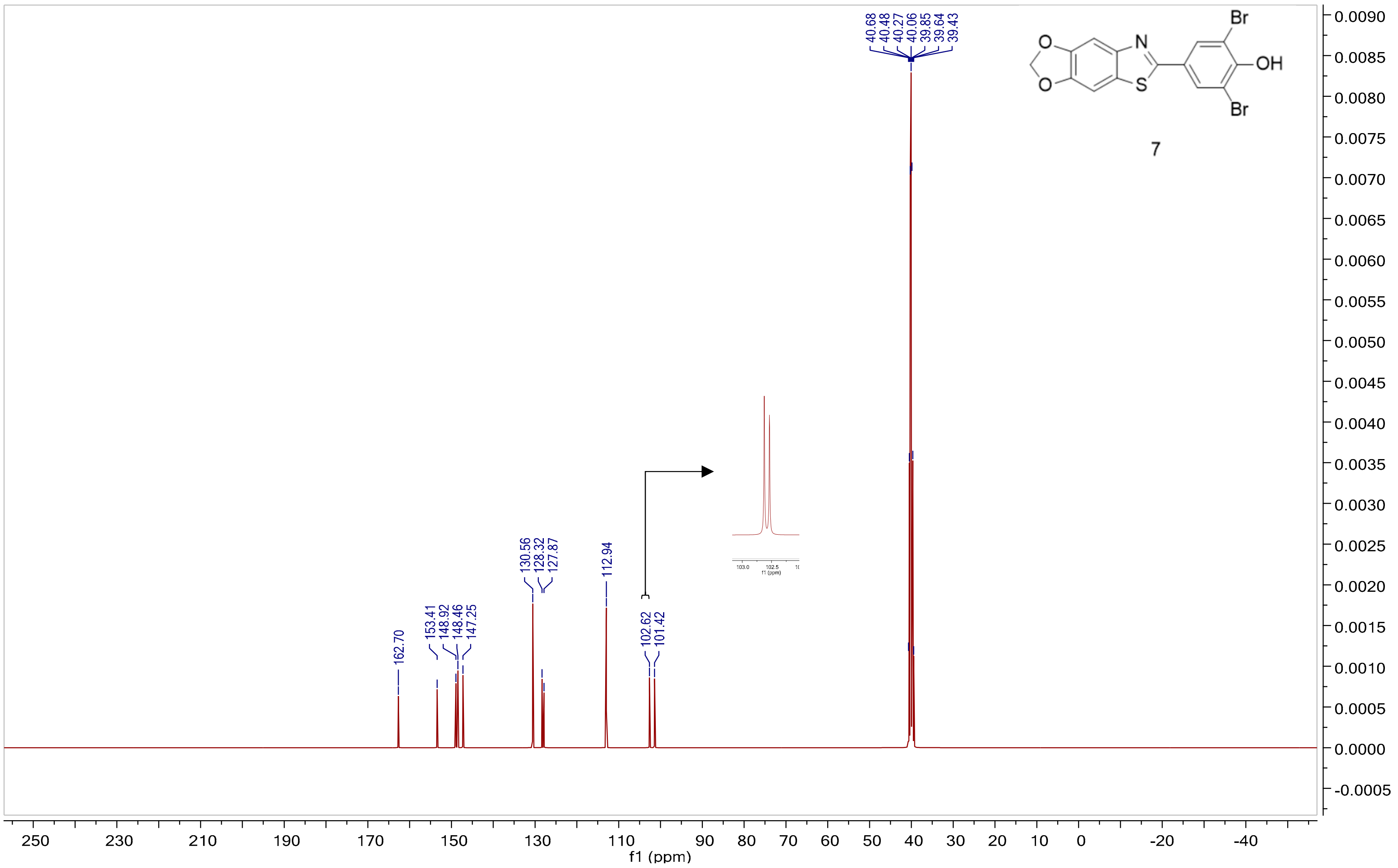


S20. $^{13}$C NMR spectrum of analog **7**

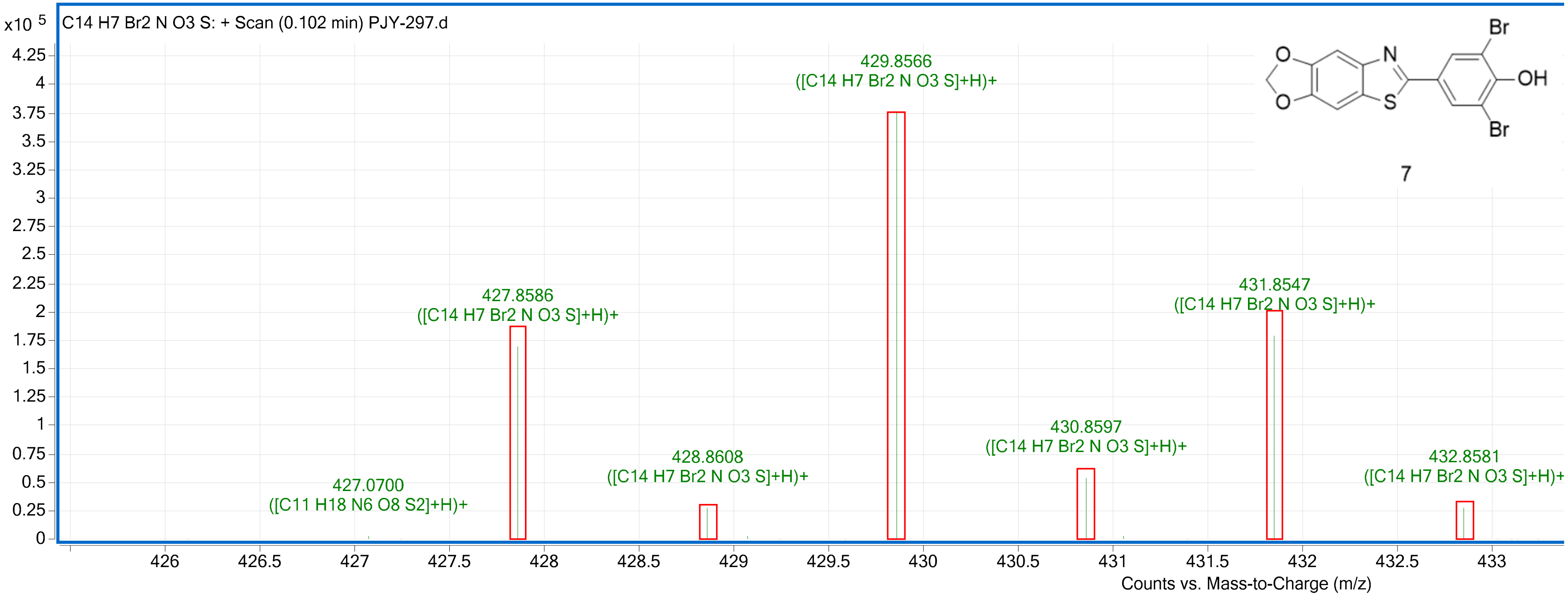


S21. HRMS (ESI+) spectrum of analog **7**

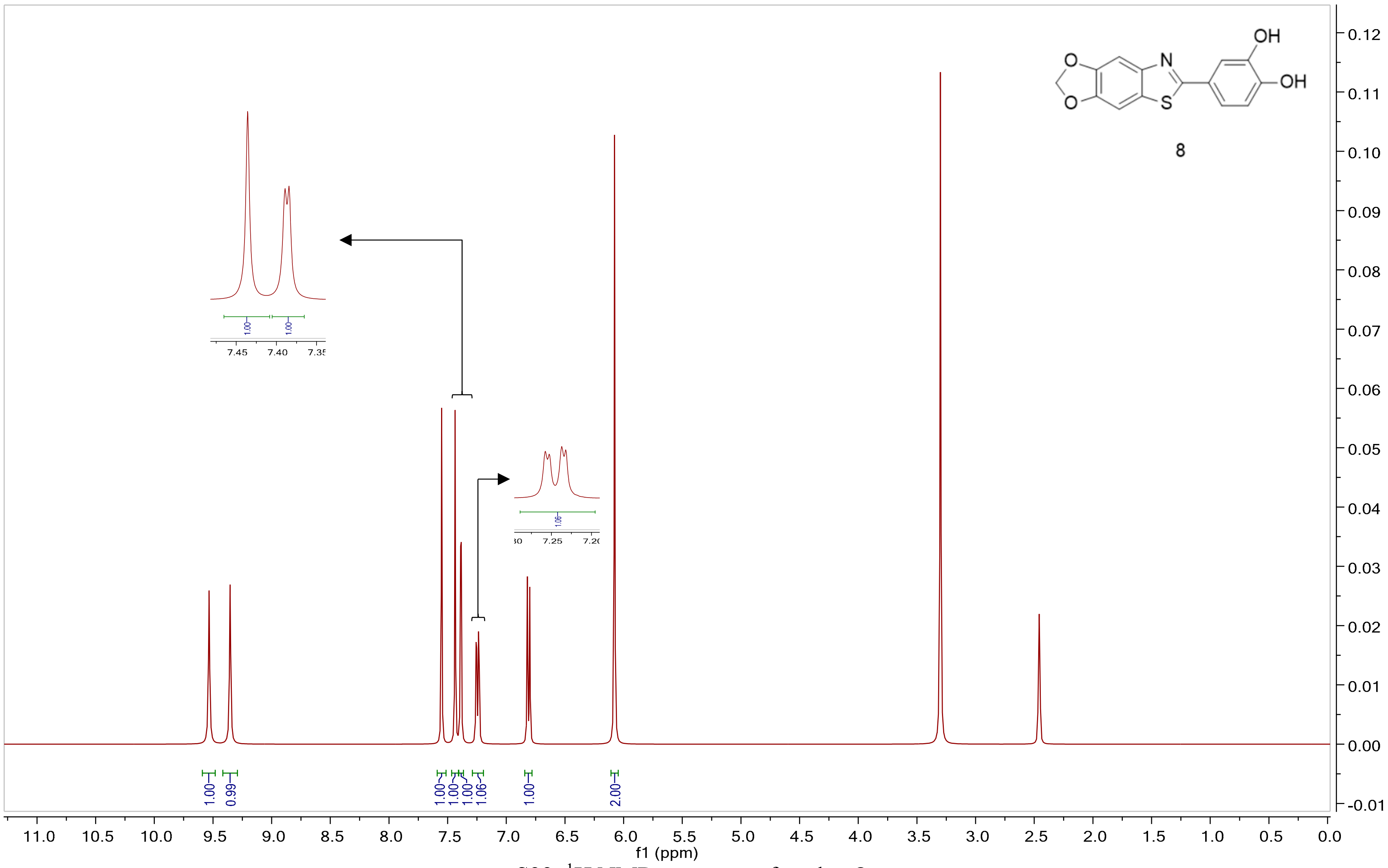


S22. $^{1}$H NMR spectrum of analog **8**

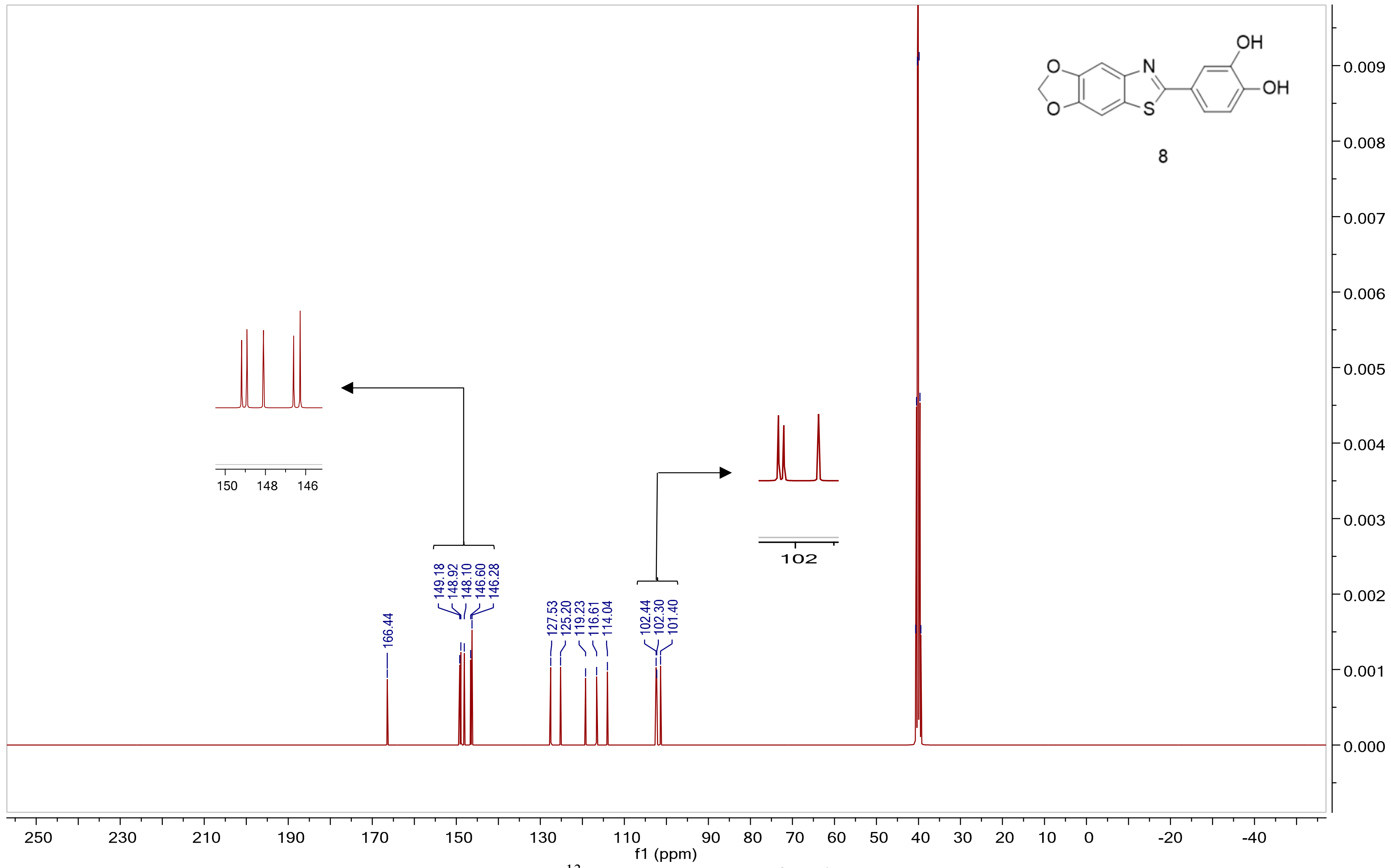


S23. $^{13}$C NMR spectrum of analog **8**

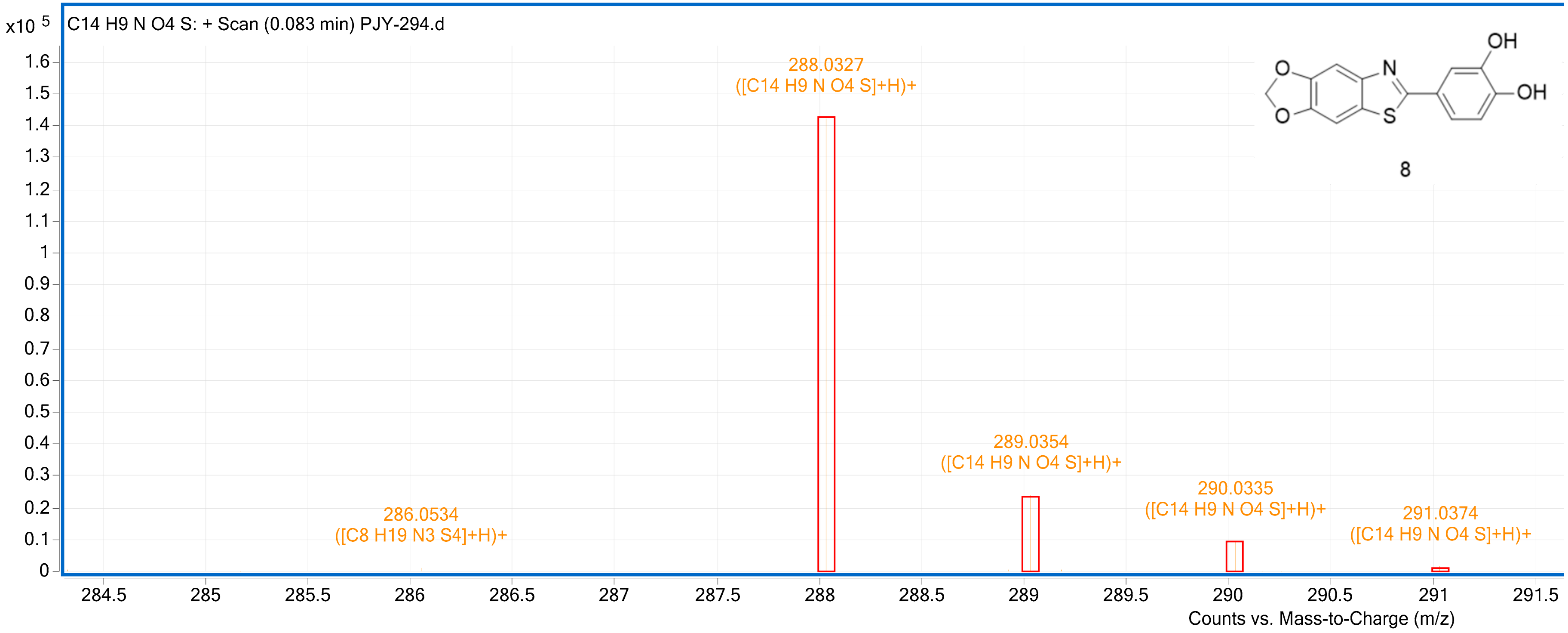


S24. HRMS (ESI+) spectrum of analog **8**

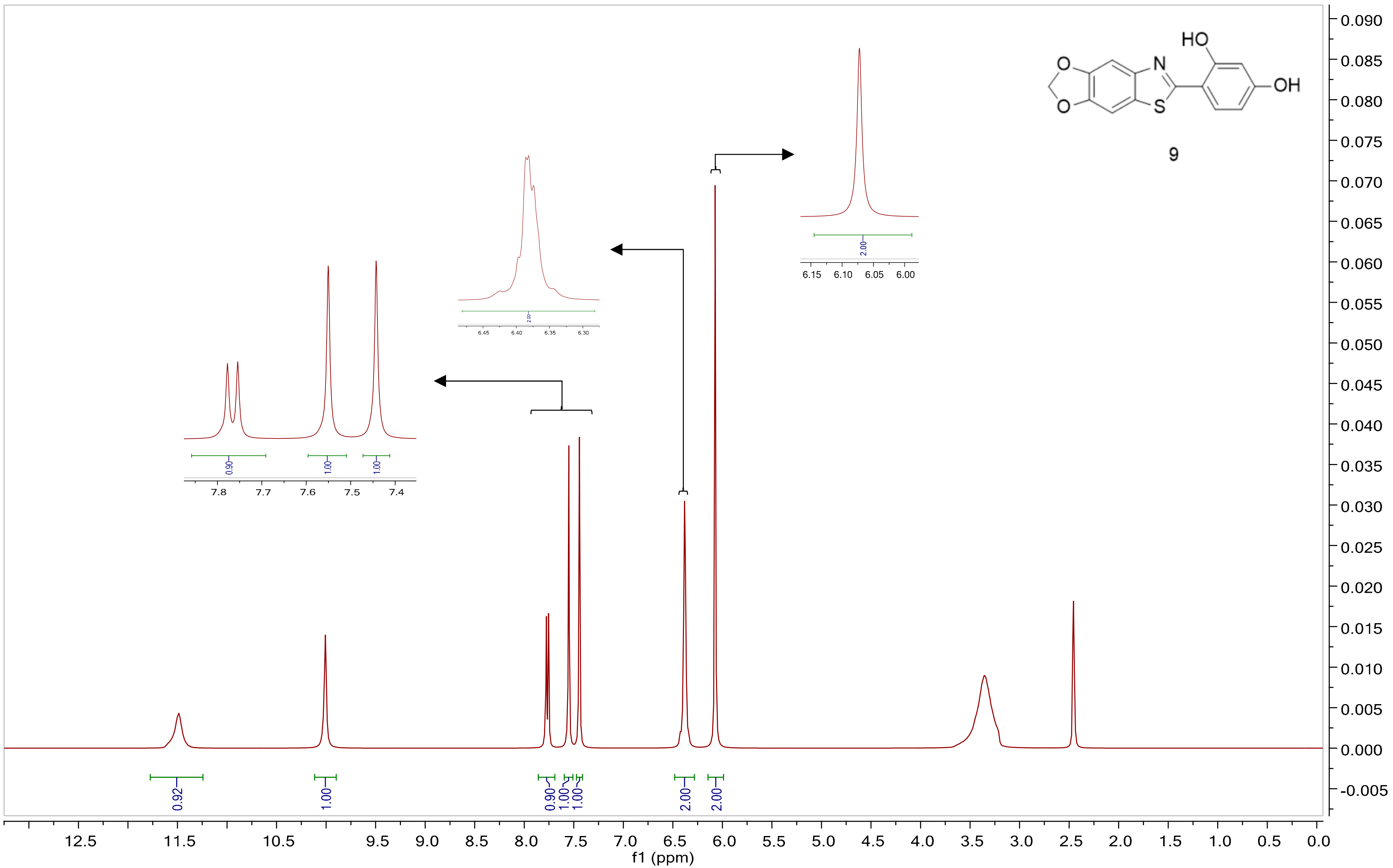


S25. $^{1}$H NMR spectrum of analog **9**

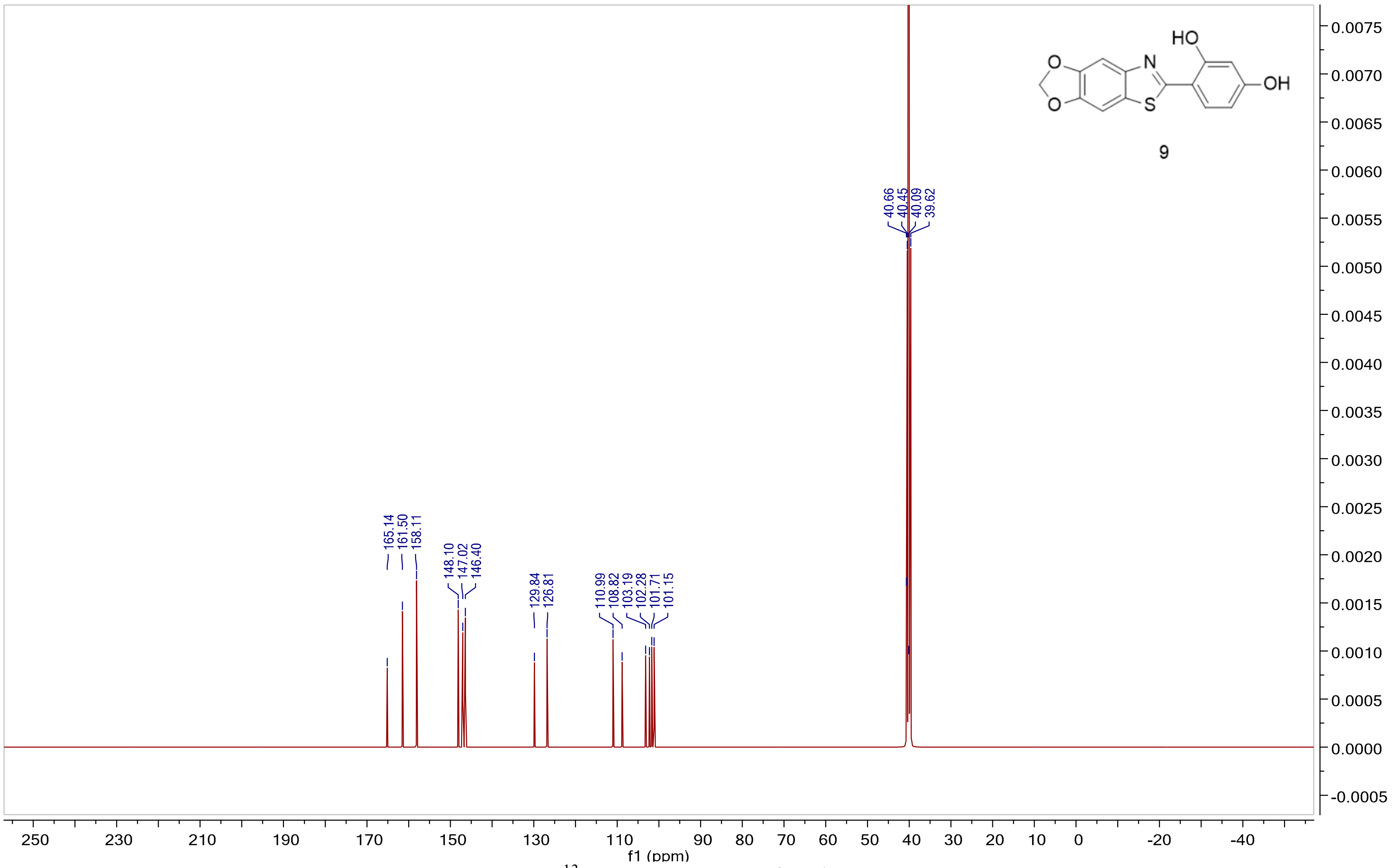


S26. $^{13}$C NMR spectrum of analog **9**

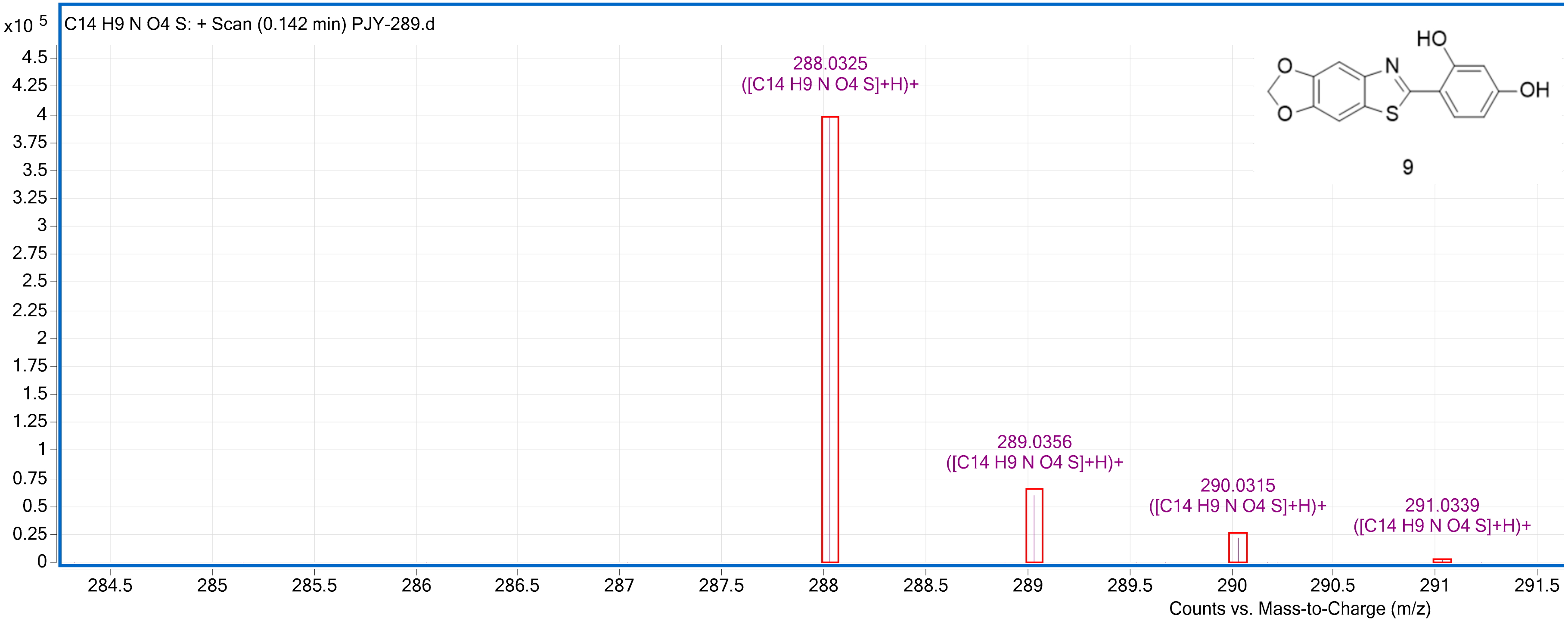


S27. HRMS (ESI+) spectrum of analog **9**

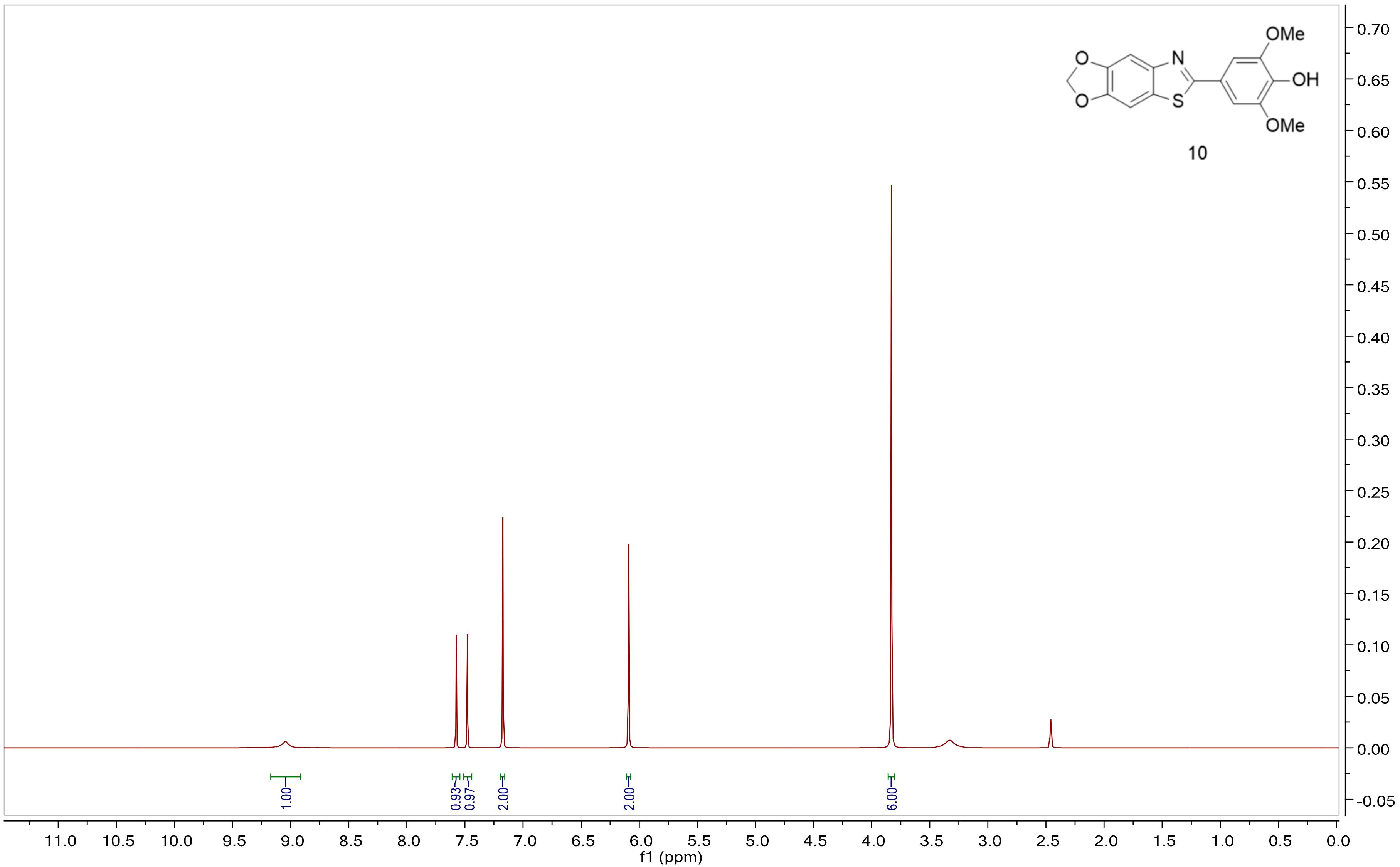


S28. $^{1}$H NMR spectrum of analog **10**

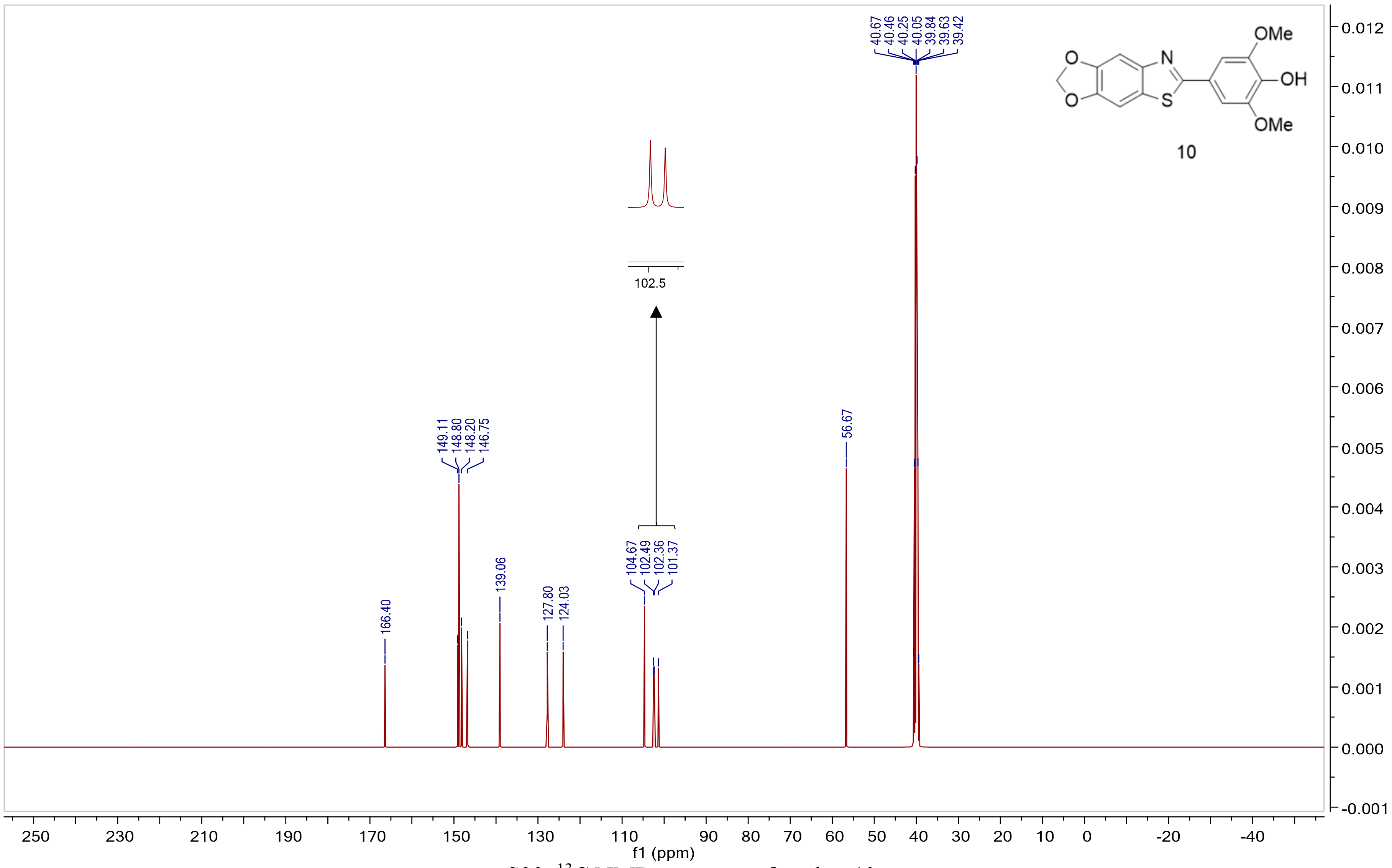


S29. $^{13}$C NMR spectrum of analog **10**

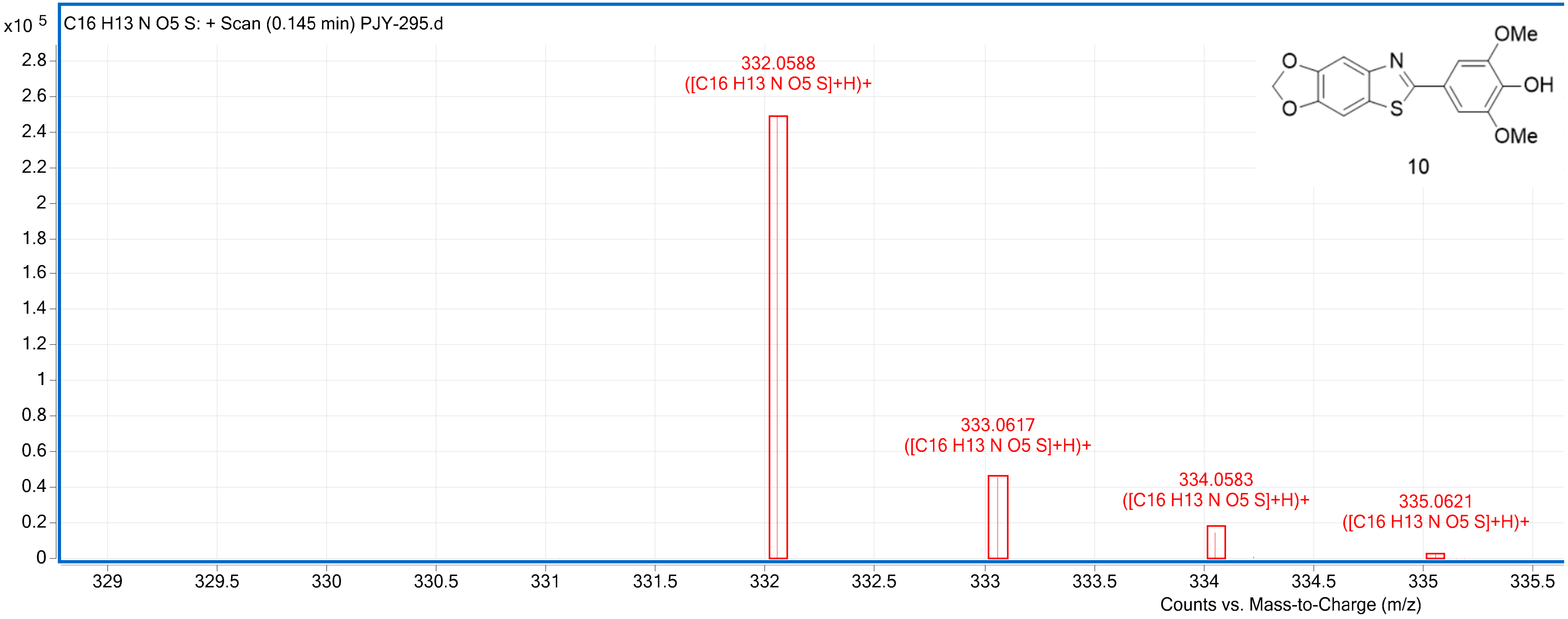


S30. HRMS (ESI+) spectrum of analog **10**

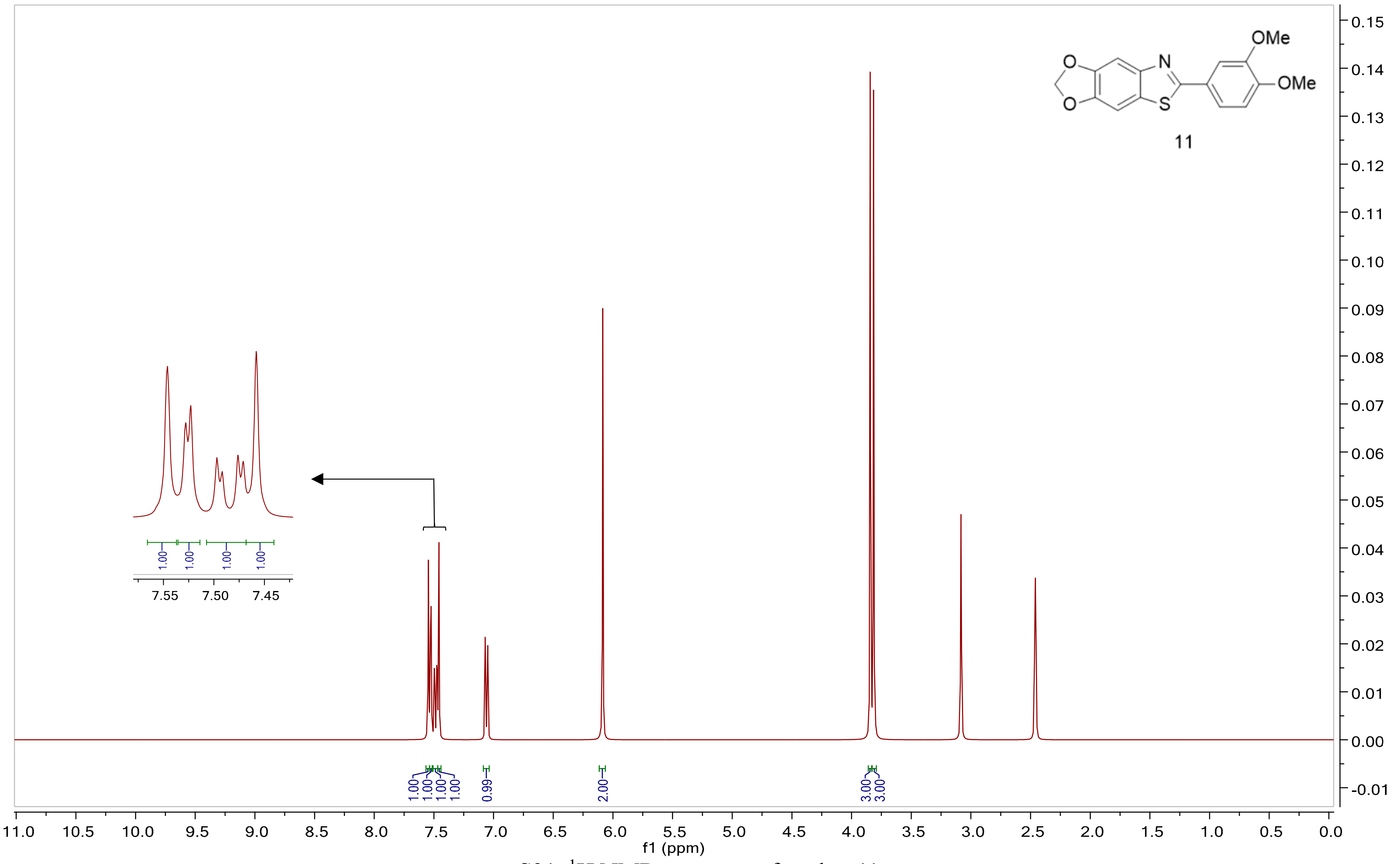


S31. $^{1}$H NMR spectrum of analog **11**

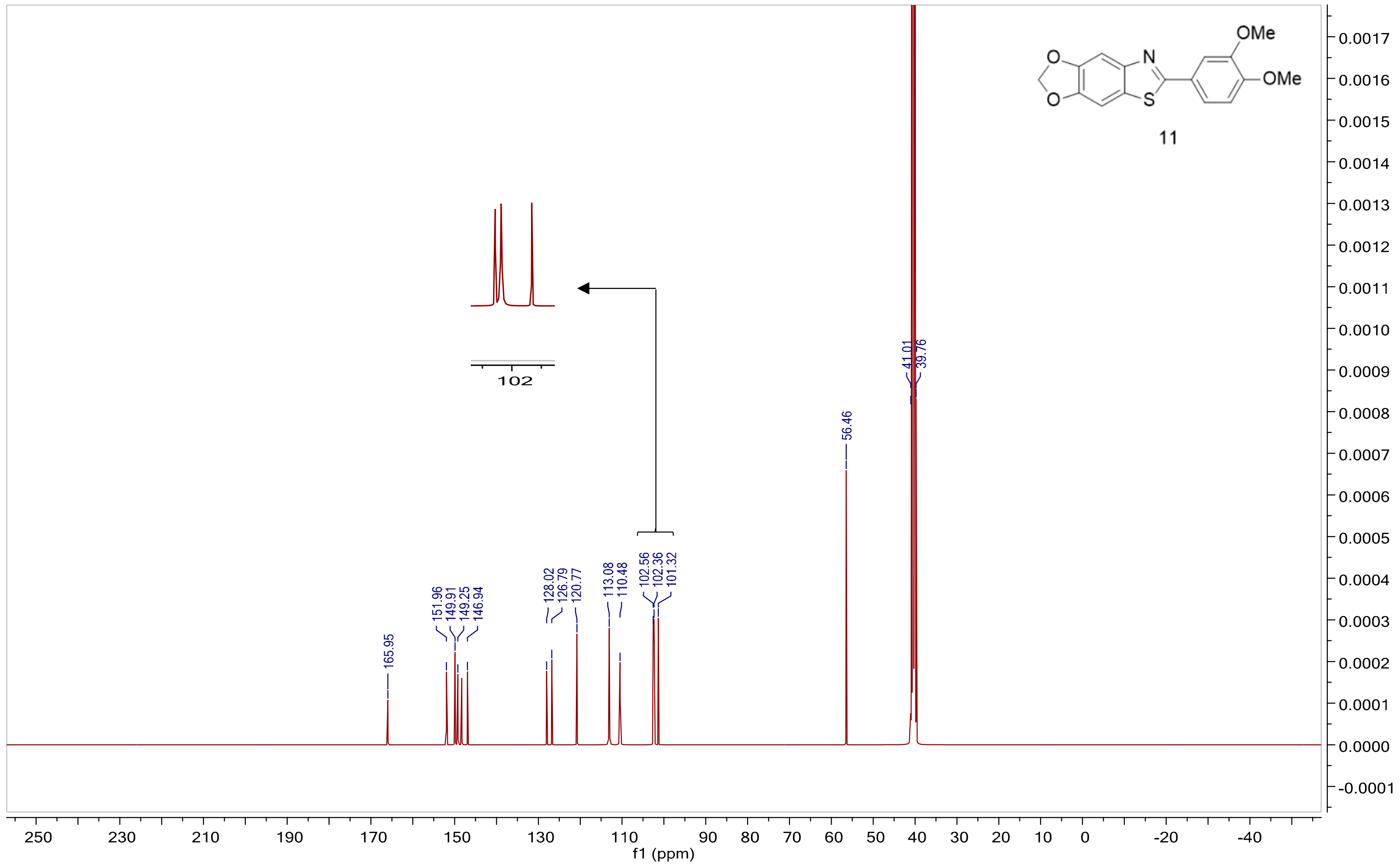


S32. $^{13}$C NMR spectrum of analog **11**

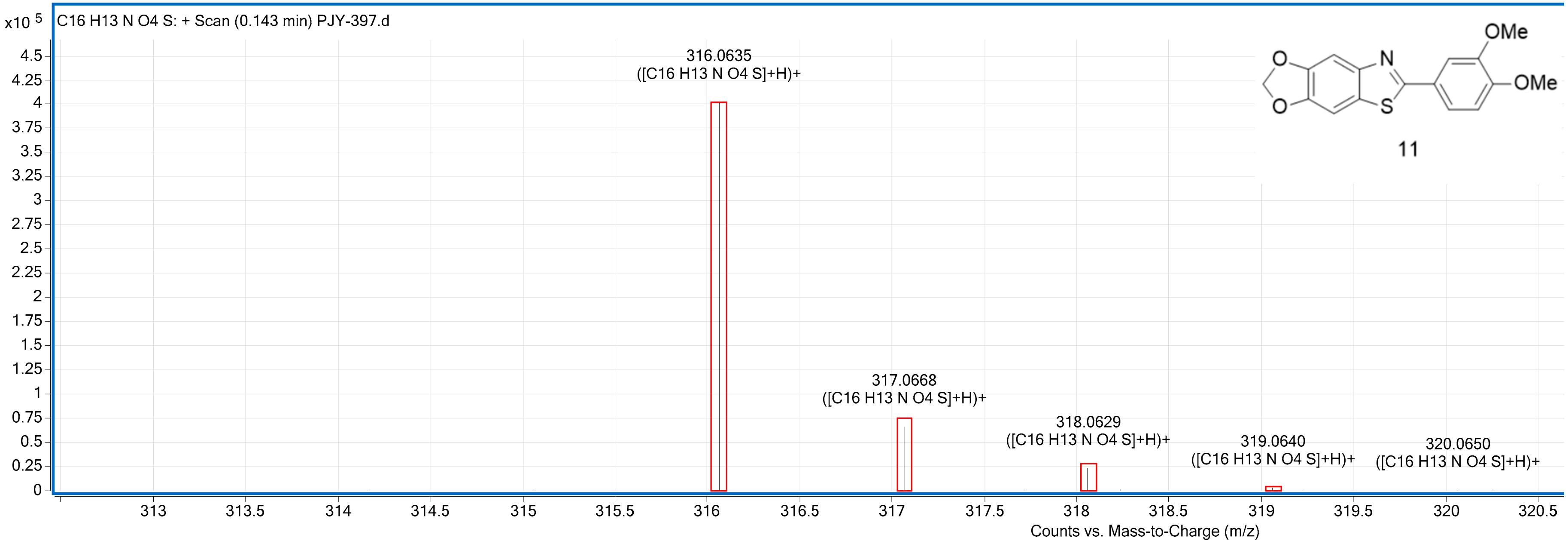


S33. HRMS (ESI+) spectrum of analog **11**

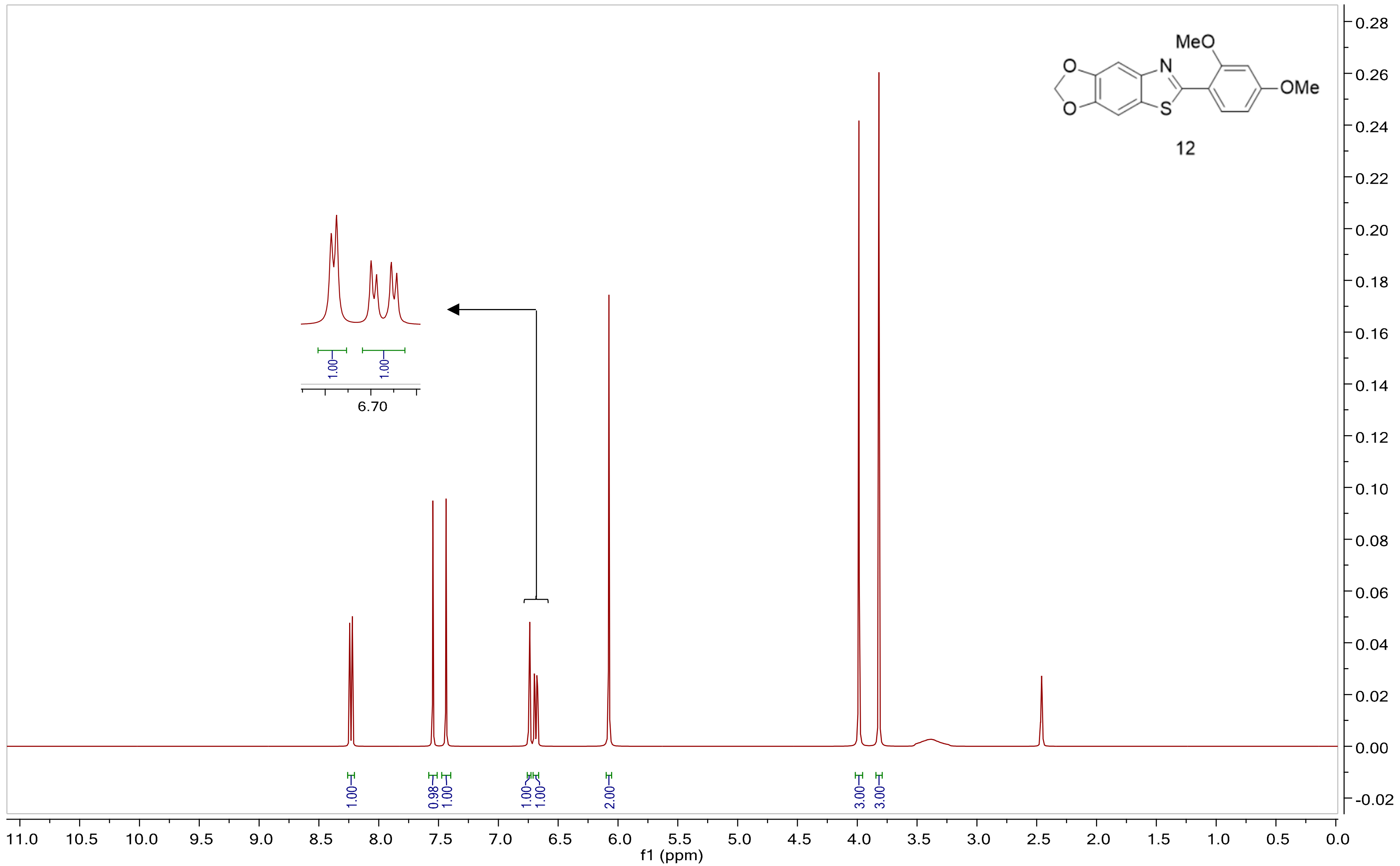


S34. $^{1}$H NMR spectrum of analog **12**

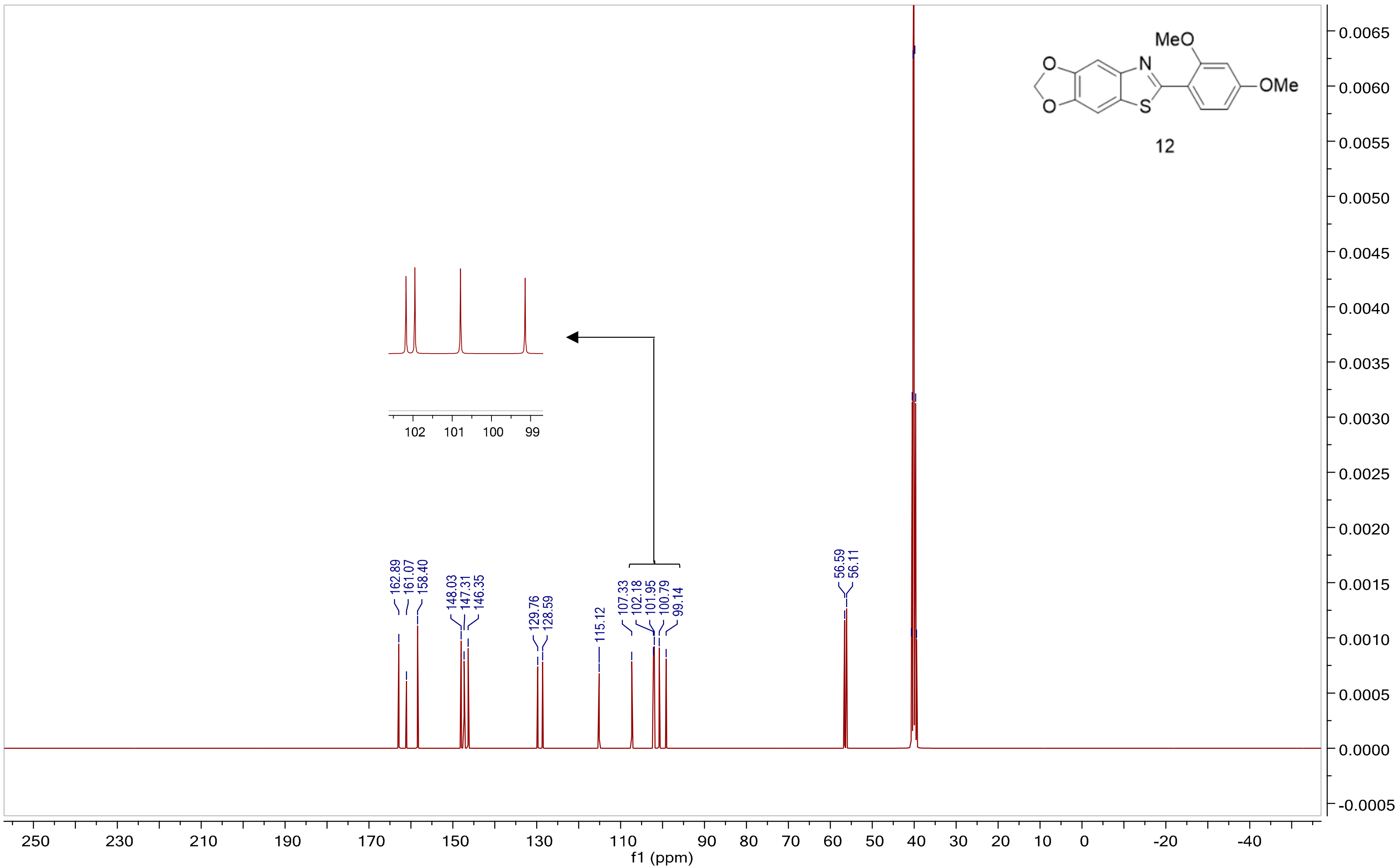


S35. $^{13}$C NMR spectrum of analog **12**

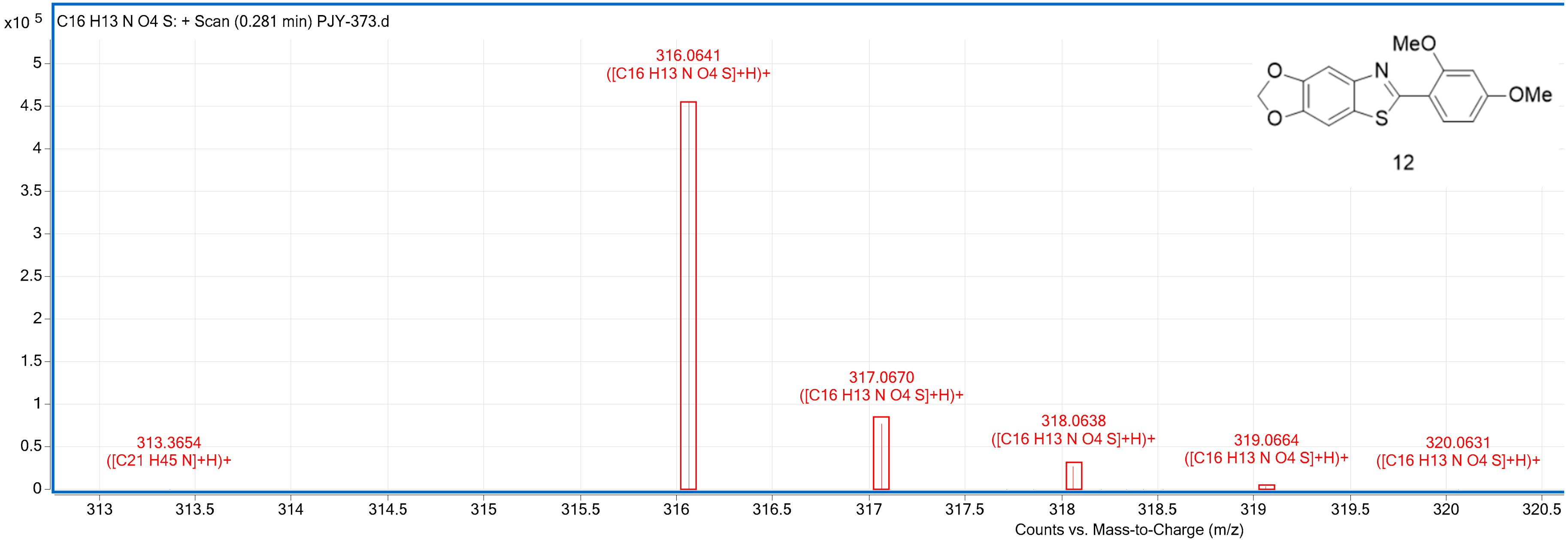


S36. HRMS (ESI+) spectrum of analog **12**

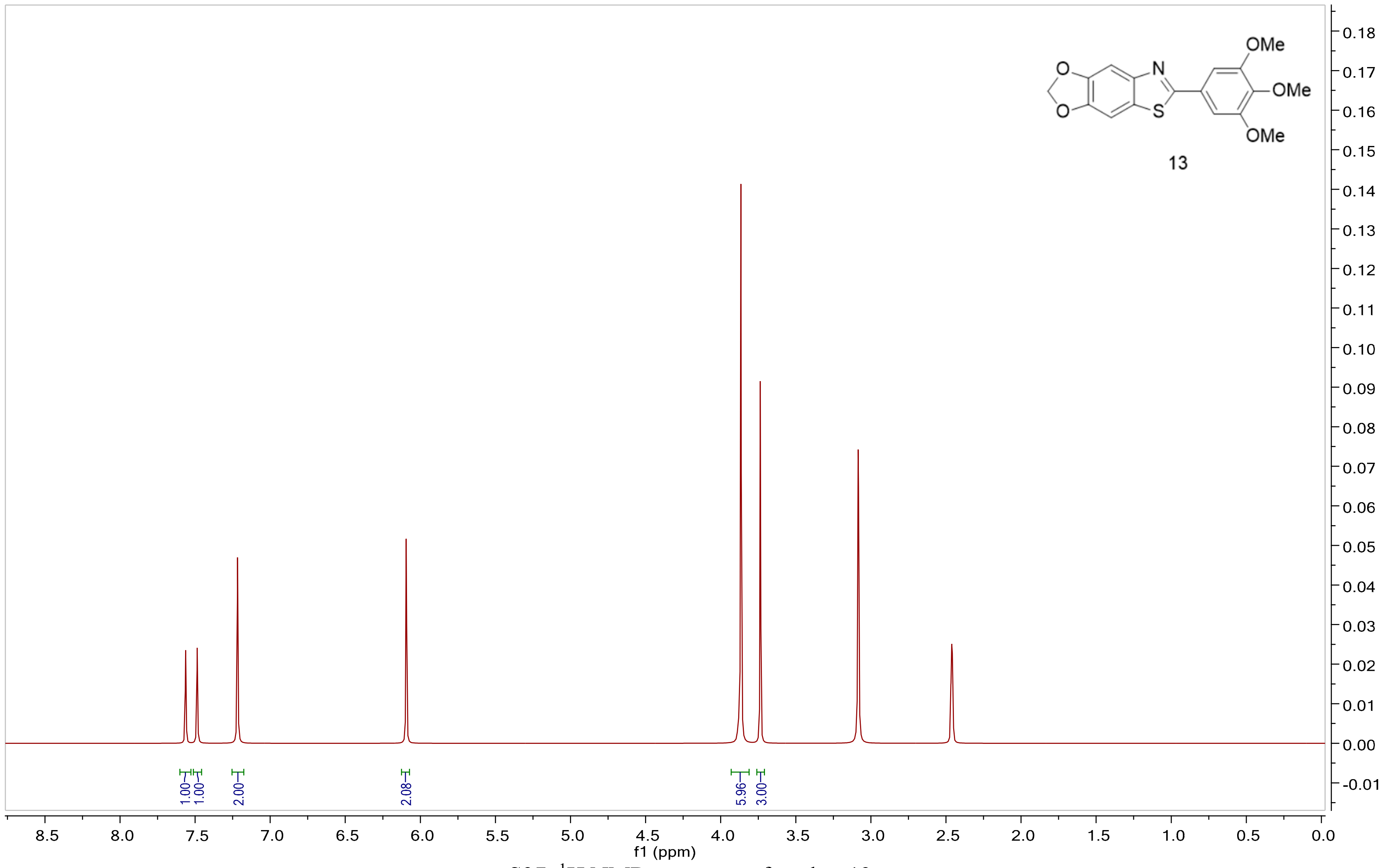


S37. $^{1}$H NMR spectrum of analog **13**

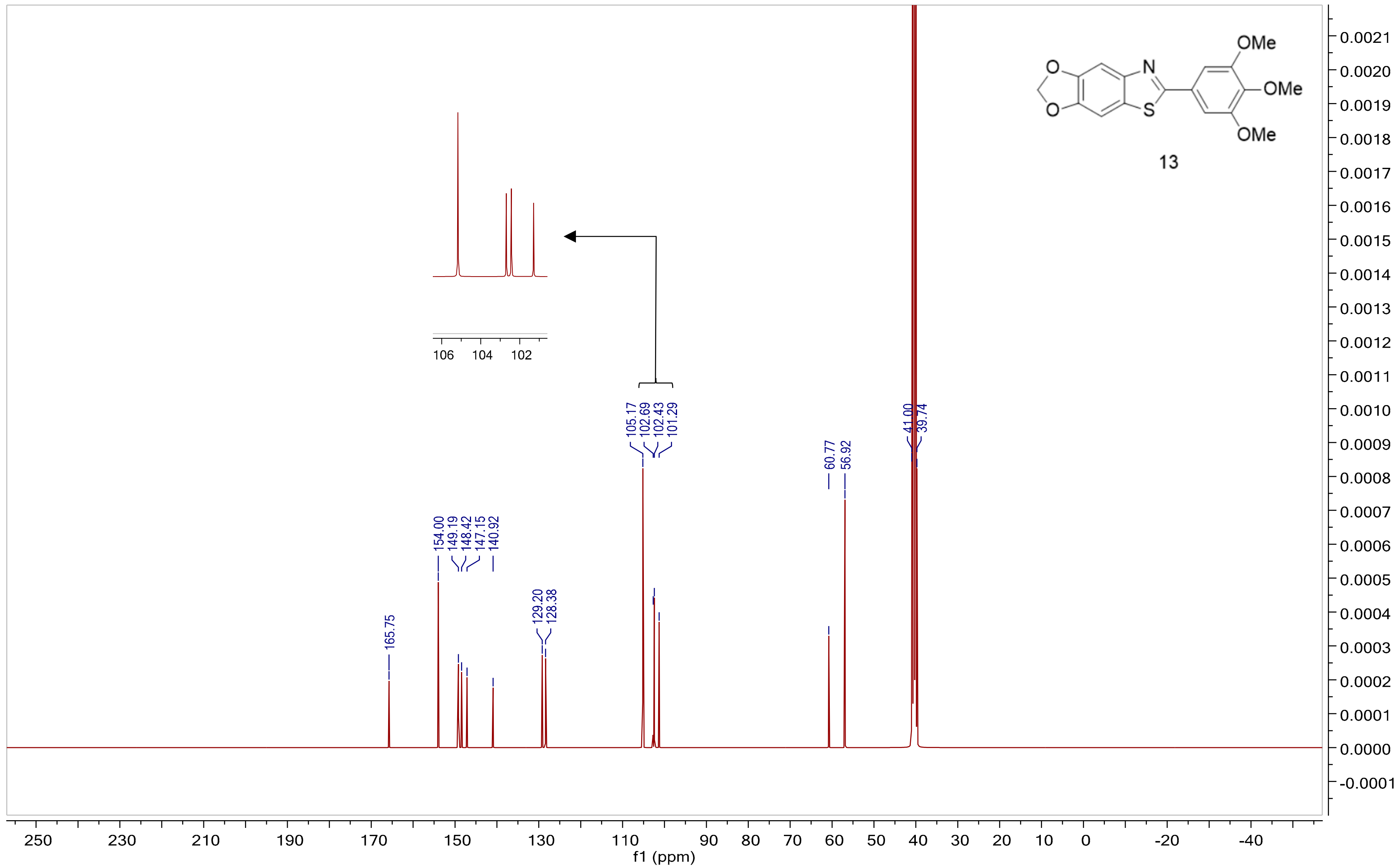


S38. $^{13}$C NMR spectrum of analog **13**

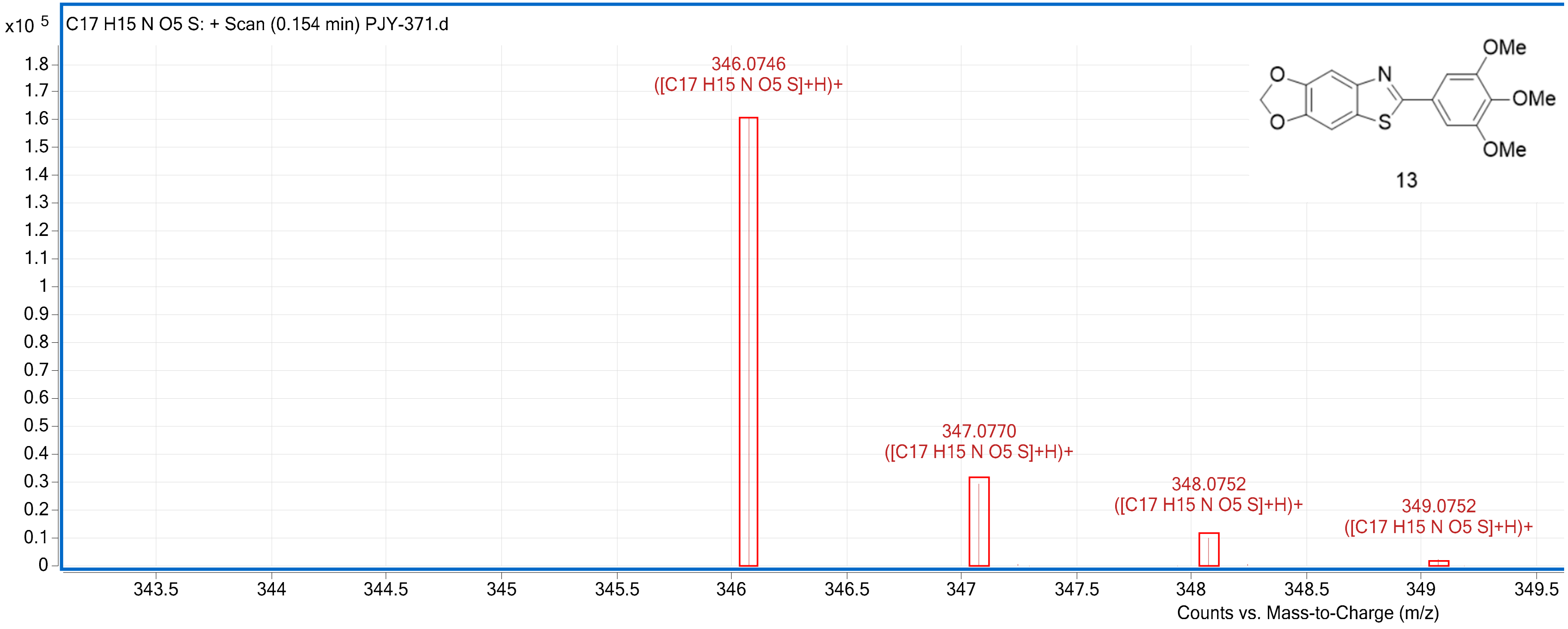


S39. HRMS (ESI+) spectrum of analog **13**

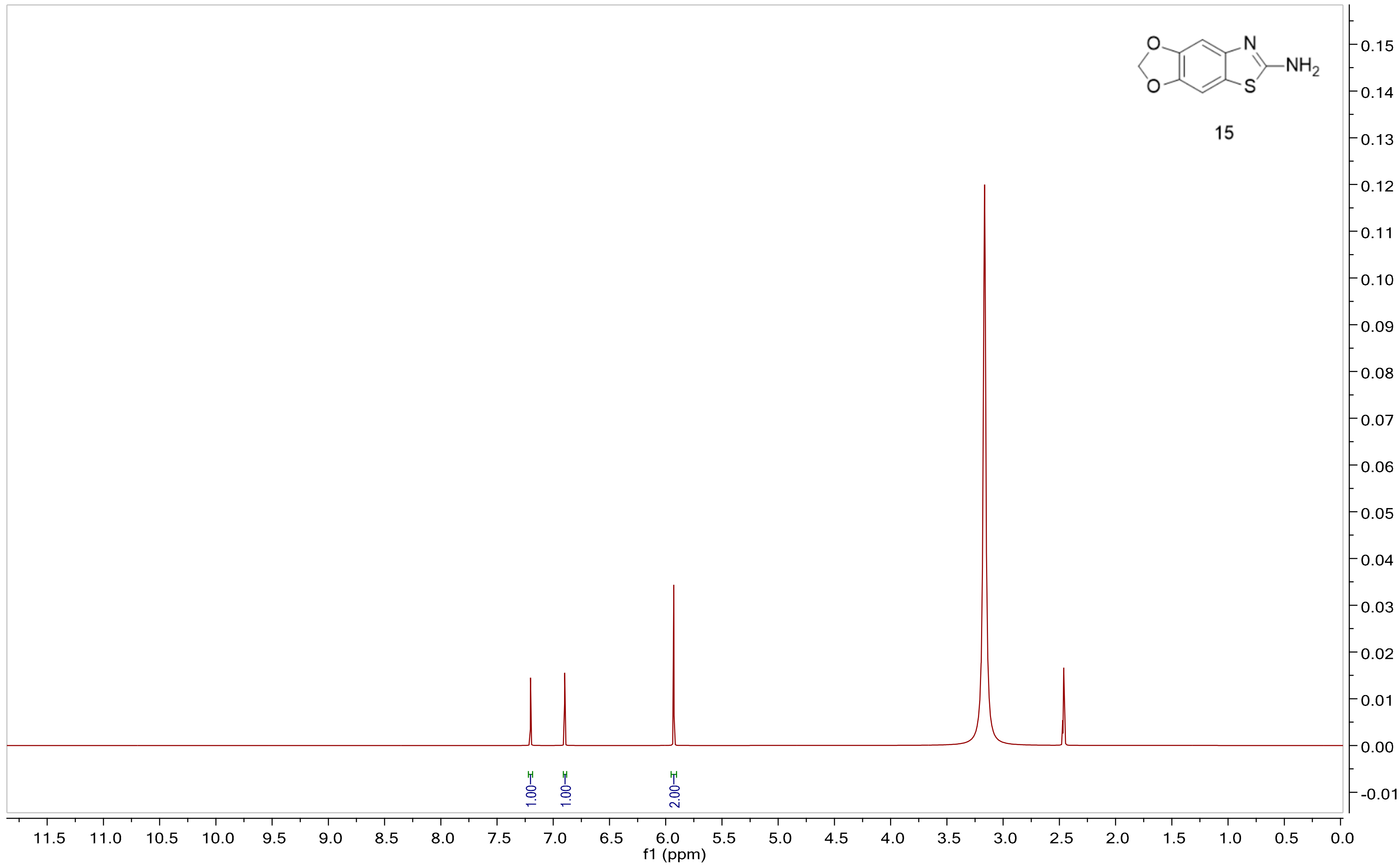


S40. $^{1}H$ NMR spectrum of analog **15**

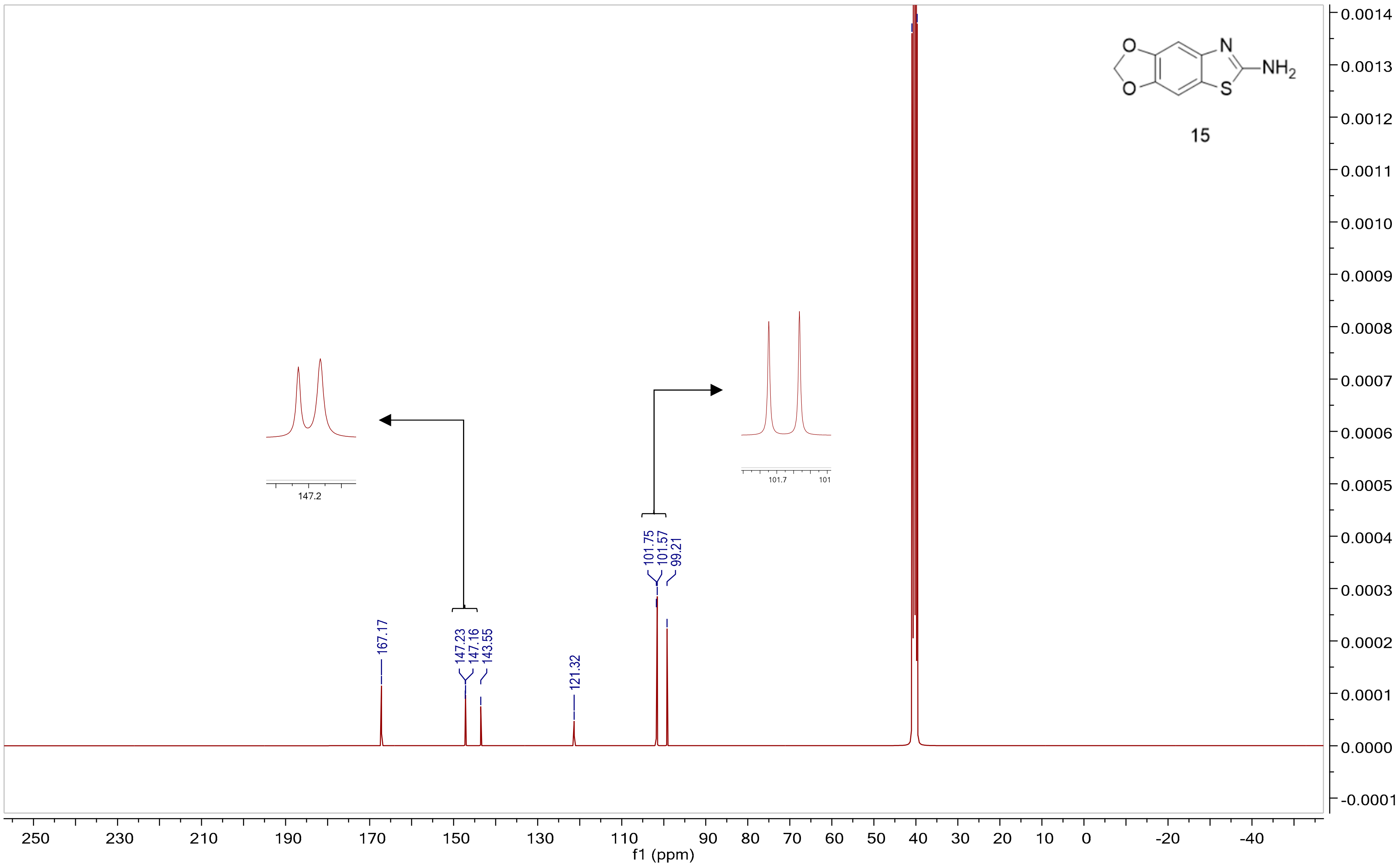


S41. $^{13}$C NMR spectrum of analog **15**

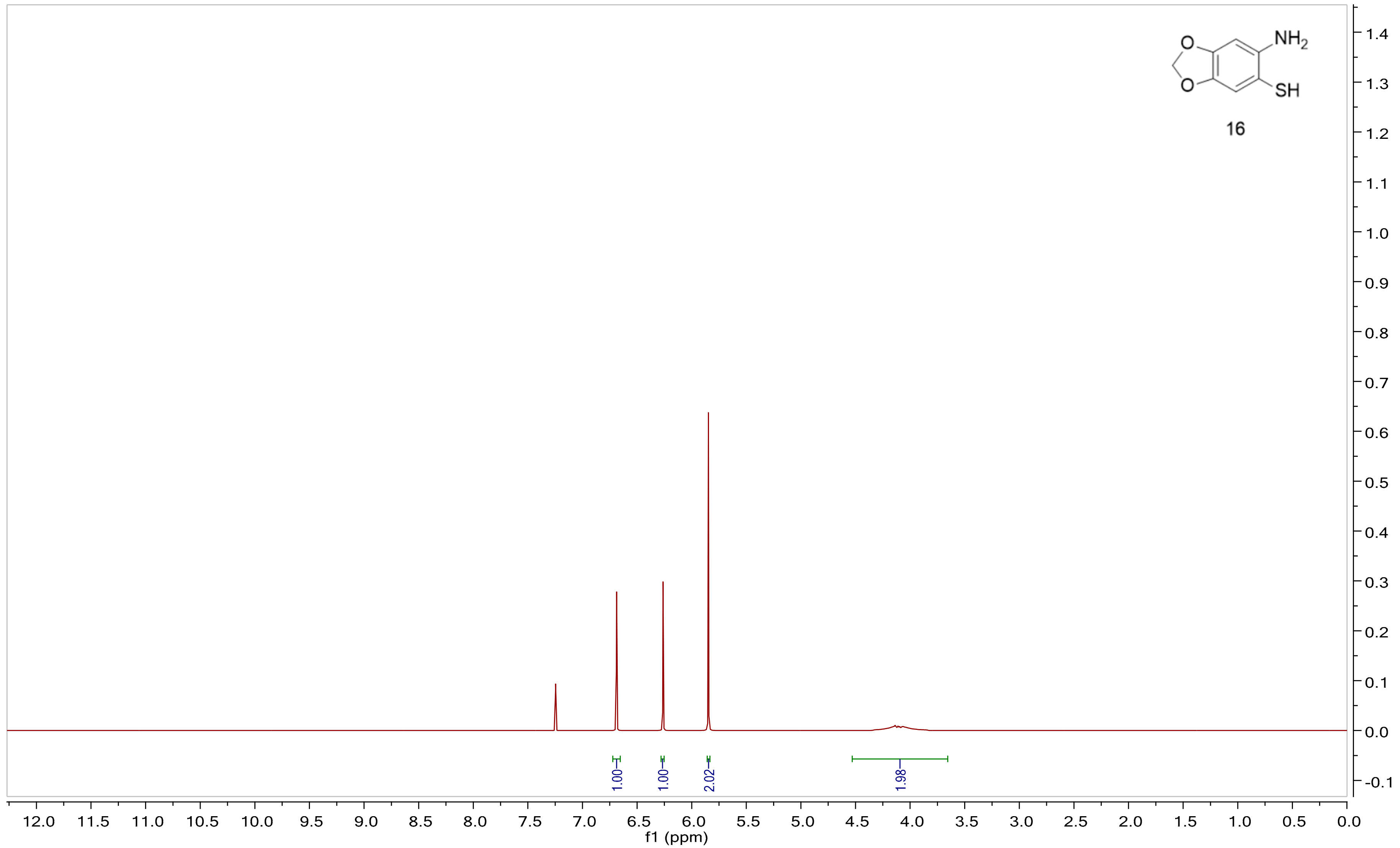


S42. $^{1}$H NMR spectrum of analog **16**

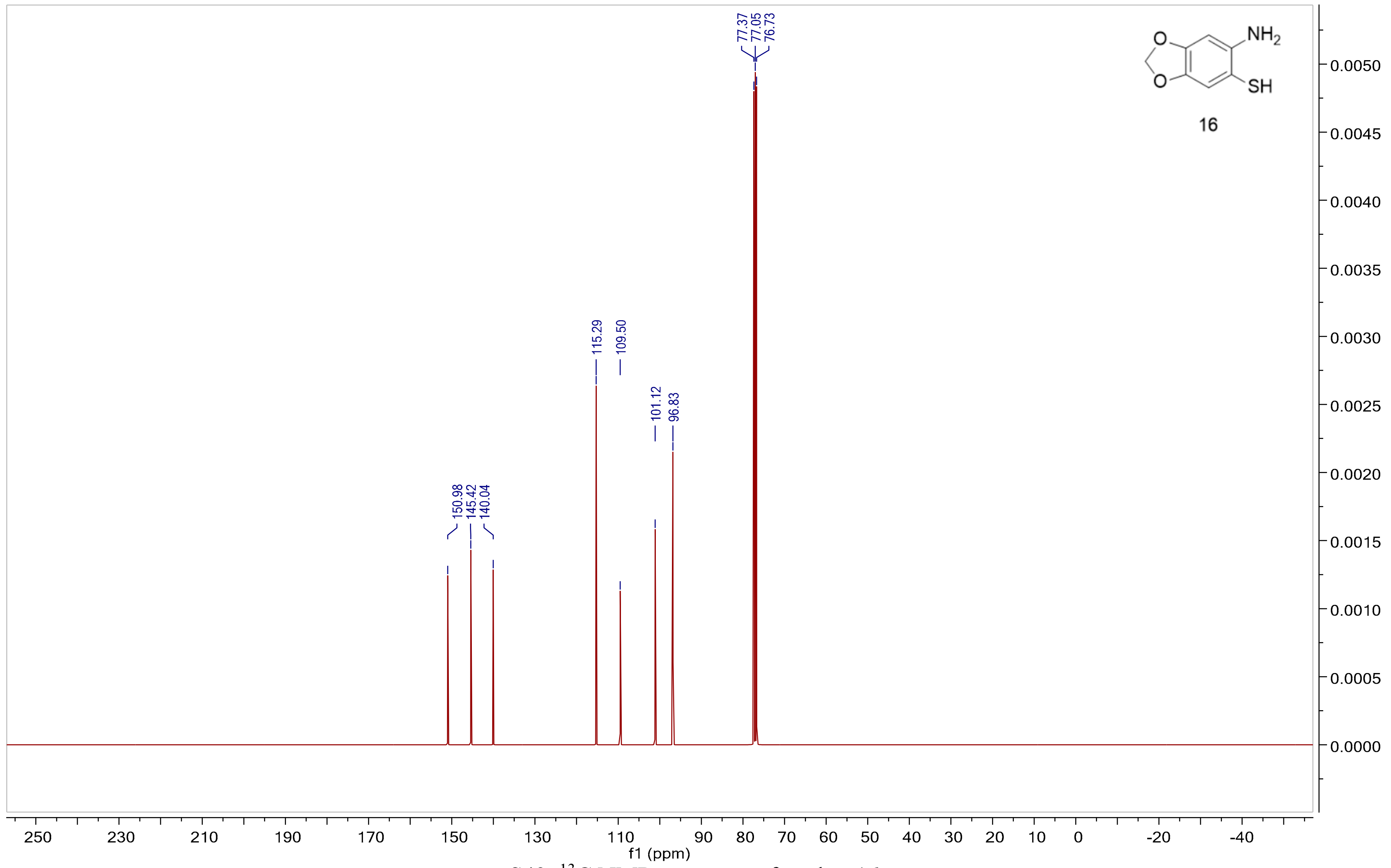


S43. $^{13}$C NMR spectrum of analog **16**

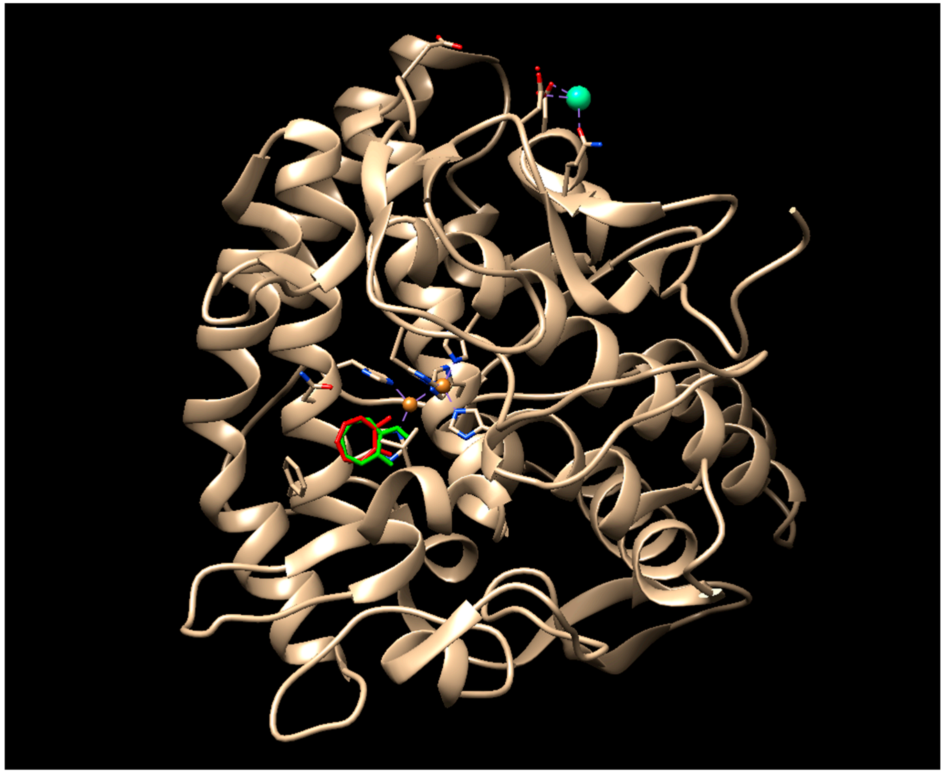

S44. Alignment of the re-docked ligand (green) and co-crystallized ligand (red) with the 2Y9X protein

The validation of the docking procedure was carried out by redocking the co-crystallized tropolone into the tyrosinase active site. In the redocking results, it was found that the re-docked tropolone reproduced the binding pose with a binding affinity of −5.5 kcal/mol. The RMSD of the co-crystallized and experimental poses was analyzed, and the RMSD value was 0.61 Å. These results suggest that the docking simulation could properly accommodate the crystallized ligand.

(A)

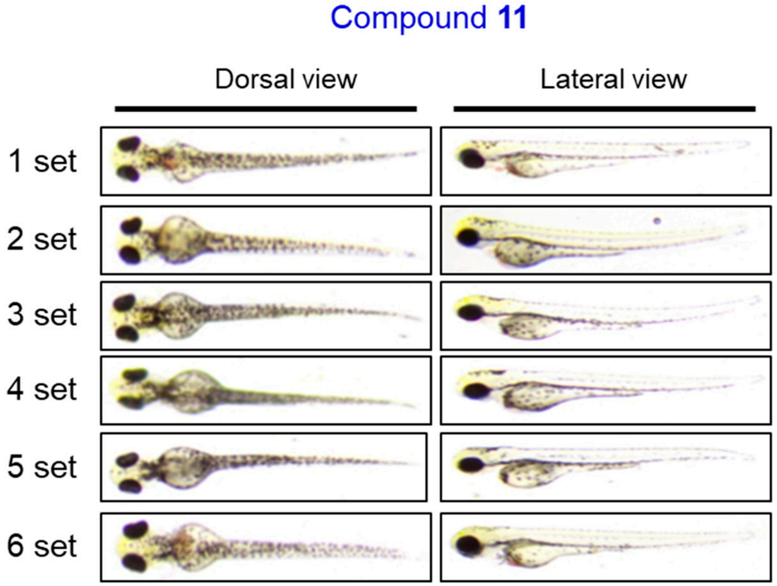


(B)

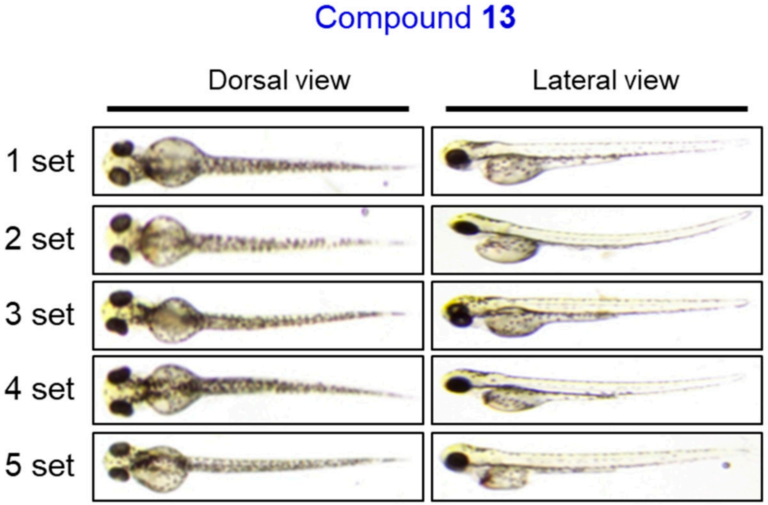


(C)

| Dorsal | Cont | KA | Comp. 2 | Comp. 5 | Comp. 11 | Comp. 12 | Comp. 13 |
|---|---|---|---|---|---|---|---|
| | 3664.16 | 2781.27 | 2809.53 | 2128.66 | 1974.77 | 1914.28 | 2945.89 |
| | 3786.92 | 2538.4 | 3234.32 | 2845.87 | 2371.34 | 2439.89 | 2143.15 |
| | 3636.68 | 2464.96 | 1958 | 2892.05 | 2786.06 | 1969.42 | 2741.06 |
| | 3106.78 | 3105.84 | 2278.02 | 3084.8 | 2264.57 | 2570.49 | 3098.3 |
| | 3502.81 | | 3173.93 | 2037.23 | 2433.84 | 1902.2 | 2332.54 |
| | | | 2588.95 | | 2363.75 | 1573.38 | |
| average | 3539.47 | 2722.618 | 2673.792 | 2597.722 | 2365.722 | 2061.61 | 2652.188 |

(D)

| Lateral | Cont | KA | Comp. 2 | Comp. 5 | Comp. 11 | Comp. 12 | Comp. 13 |
|---|---|---|---|---|---|---|---|
| | 2458.91 | 1805.61 | 2592.57 | 1513.32 | 1154.95 | 1378.32 | 1594.14 |
| | 2538.8 | 1624.35 | 1996.95 | 1818.32 | 2007.84 | 1226.5 | 1350.59 |
| | 2233.1 | 1659.42 | 1607.61 | 2016.95 | 1337.02 | 1310.67 | 2107.6 |
| | 1851.5 | 2284.97 | 1552.74 | 2086.06 | 1425.73 | 1468.51 | 1685.64 |
| | 2382.33 | | 2156.66 | 1492.18 | 1480.53 | 1390.93 | 1674.67 |
| | | | 1728.21 | | 1503.3 | 1209.23 | |
| average | 2292.928 | 1843.588 | 1939.123 | 1785.366 | 1484.895 | 1330.693 | 1682.528 |

S45. Depigmentation results performed using zebrafish embryos. (A) Dorsal and lateral views of zebrafish larvae treated with compound **11** (A) and **13** (B). Results of measured pigmentation area in dorsal (C) and lateral (D) views of zebrafish larvae.

**Substrate: L-Dopa**

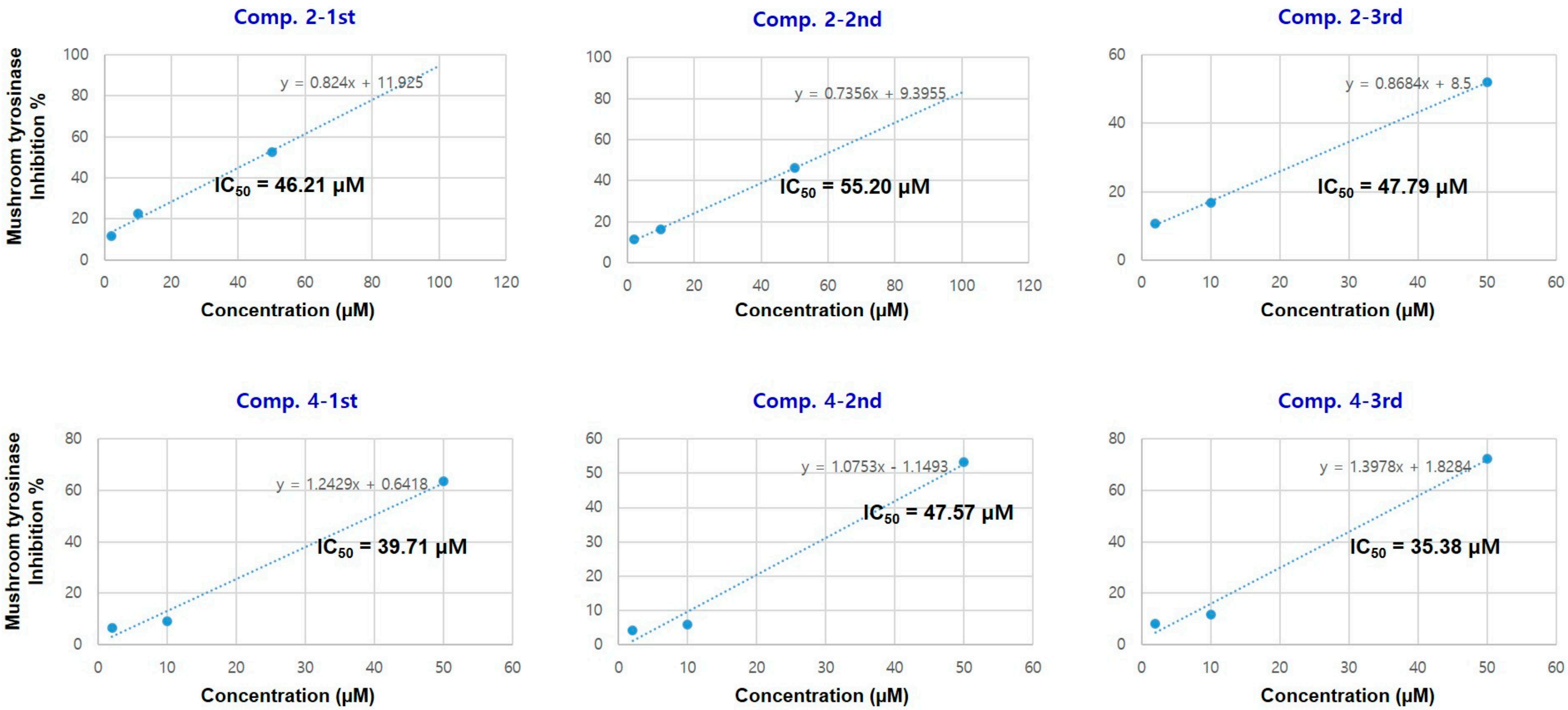


S46. Graphs used to determine $IC_{50}$ values for compounds **2** and **4** in the presence of L-dopa.

**Substrate: L-Dopa**

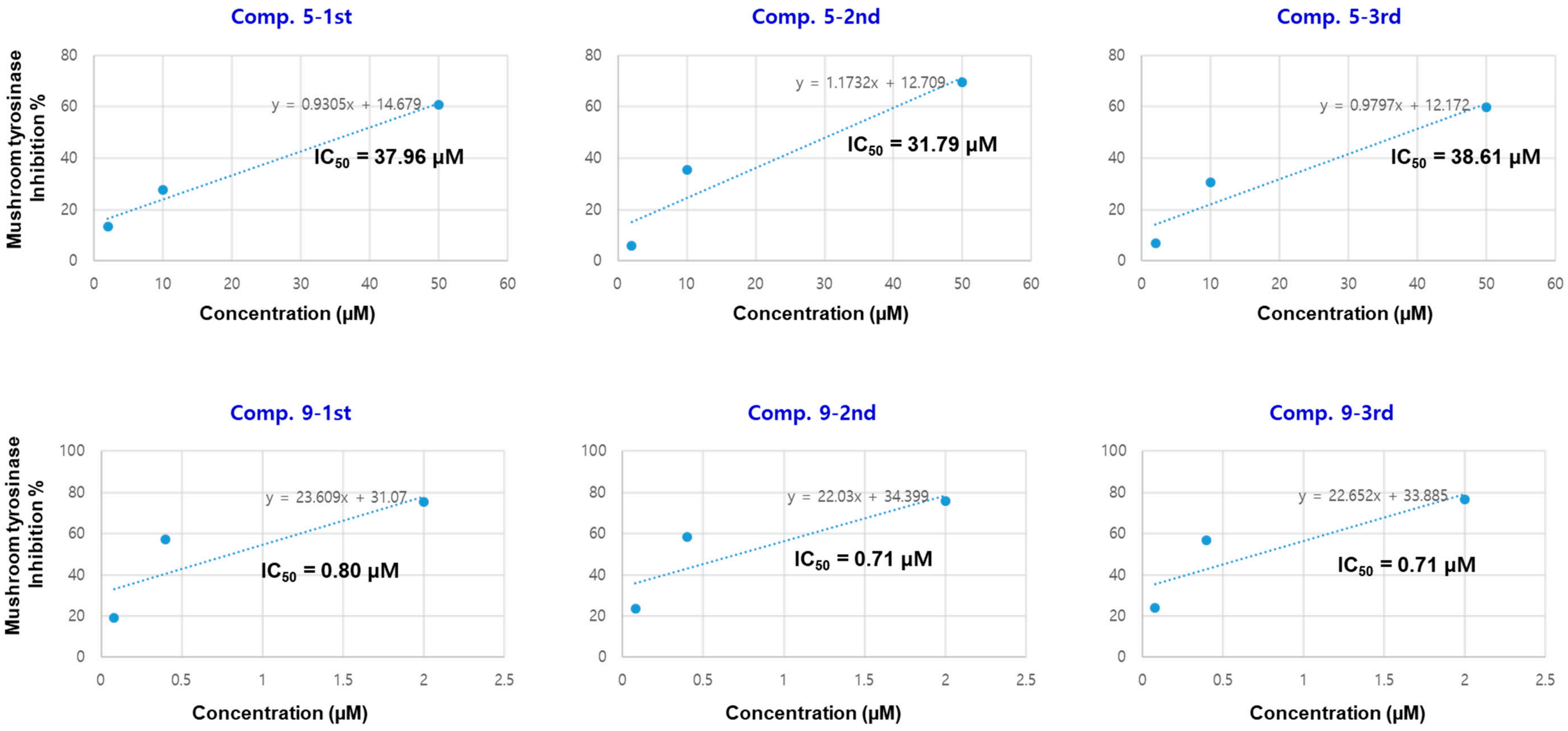


S47. Graphs used to determine $IC_{50}$ values for compounds **5** and **9** in the presence of L-dopa.

**Substrate: L-Dopa**

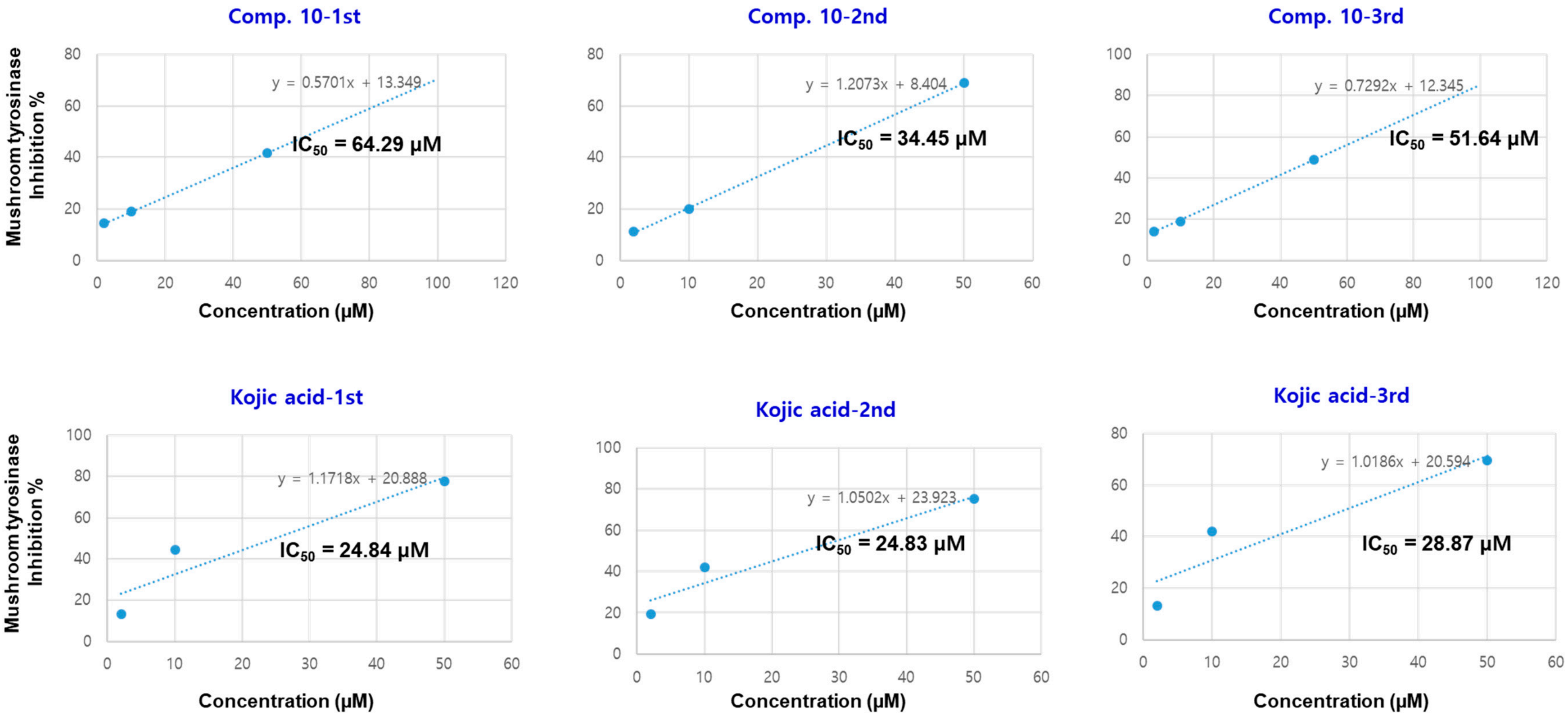


S48. Graphs used to determine $IC_{50}$ values for compound **10** and kojic acid in the presence of L-dopa.

**Substrate: L-Tyrosine**

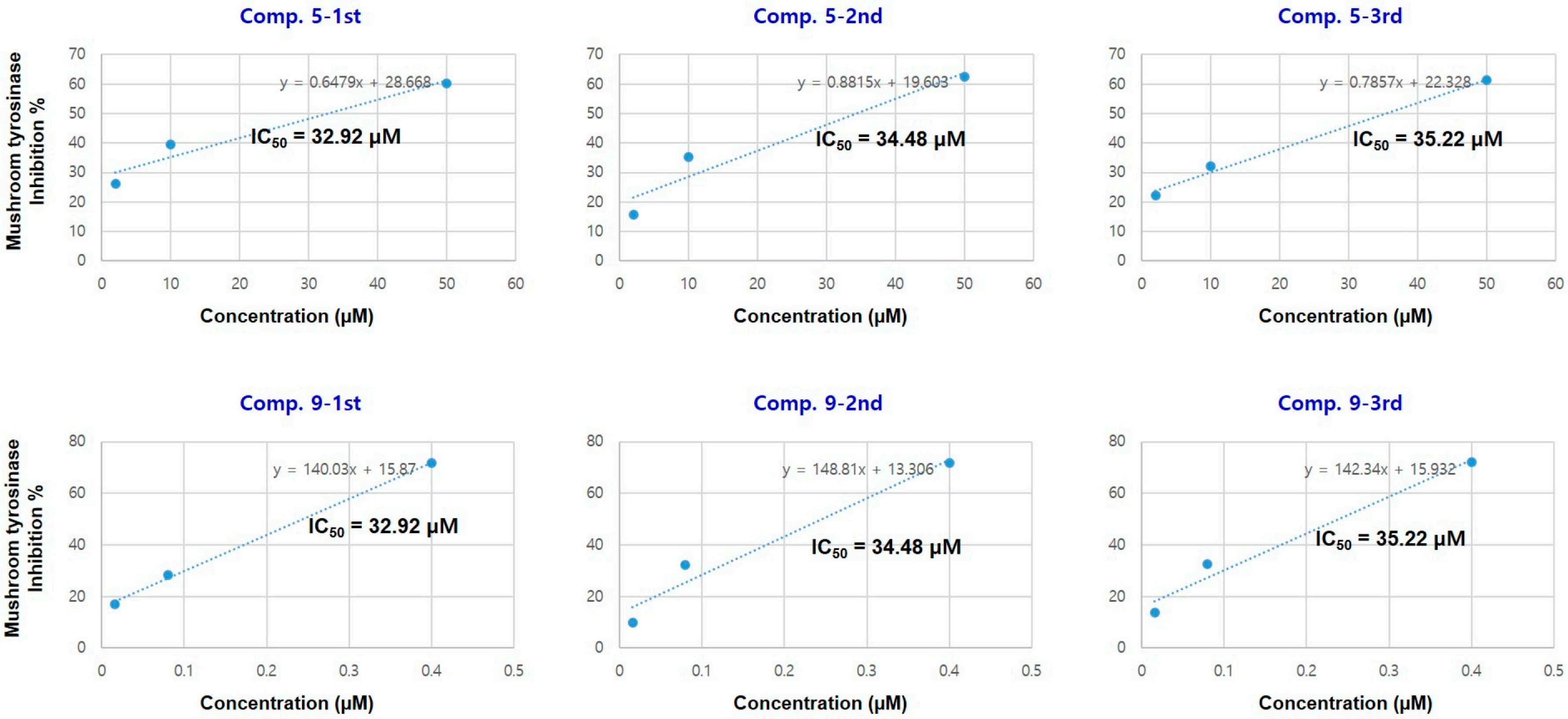


S49. Graphs used to determine $IC_{50}$ values for compounds **5** and **9** in the presence of L-tyrosine.

**Substrate: L-Tyrosine**

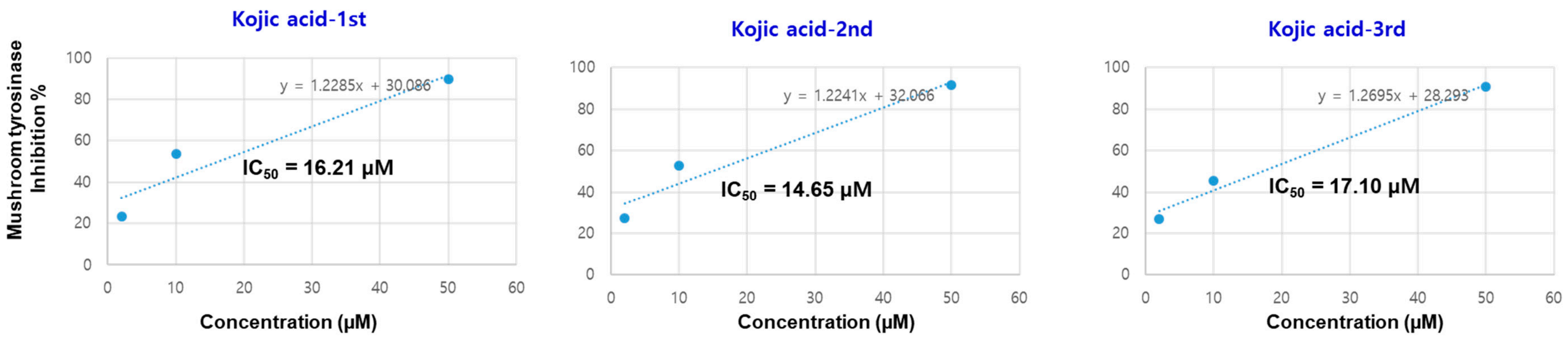


S50. Graphs used to determine an $IC_{50}$ value for kojic acid in the presence of L-tyrosine.

**(A)** **0 min**

Inhibitor concentration (μM)

| | | 1 | 2 | 3 | 4 | 5 | 6 | 7 | 8 | |
|---|---|---|---|---|---|---|---|---|---|---|
| A | 0 | 0.0932 | 0.1042 | 0.0947 | 0.0799 | 0.0773 | 0.0567 | 0.0504 | 0.0442 | Sample group |
| B | 20 | 0.1012 | 0.1039 | 0.0796 | 0.0717 | 0.0605 | 0.0515 | 0.0475 | 0.044 | |
| C | 40 | 0.1087 | 0.1048 | 0.0903 | 0.0739 | 0.0662 | 0.0571 | 0.0524 | 0.0425 | |
| D | 80 | 0.2318 | 0.1987 | 0.1864 | 0.1691 | 0.193 | 0.1181 | 0.1851 | 0.1231 | |
| E | 0 | 0.04 | 0.0398 | 0.0397 | 0.0398 | 0.0392 | 0.0392 | 0.038 | 0.0385 | Color control group |
| F | 20 | 0.04 | 0.0403 | 0.0406 | 0.0405 | 0.043 | 0.0395 | 0.0392 | 0.0394 | |
| G | 40 | 0.0436 | 0.0421 | 0.0423 | 0.041 | 0.0411 | 0.0473 | 0.0466 | 0.0411 | |
| H | 80 | 0.1262 | 0.1405 | 0.115 | 0.1595 | 0.1647 | 0.1051 | 0.1452 | 0.1187 | |

**(B)** **10 min**

Inhibitor concentration (μM)

| | | 1 | 2 | 3 | 4 | 5 | 6 | 7 | 8 | |
|---|---|---|---|---|---|---|---|---|---|---|
| A | 0 | 0.3215 | 0.312 | 0.2875 | 0.2338 | 0.1999 | 0.1301 | 0.0974 | 0.0682 | Sample group |
| B | 20 | 0.3026 | 0.3055 | 0.2638 | 0.2045 | 0.1462 | 0.1081 | 0.0881 | 0.0698 | |
| C | 40 | 0.3335 | 0.2916 | 0.2412 | 0.2023 | 0.1512 | 0.1144 | 0.0889 | 0.0719 | |
| D | 80 | 0.3955 | 0.3504 | 0.3537 | 0.2315 | 0.2056 | 0.1503 | 0.2148 | 0.2837 | |
| E | 0 | 0.0403 | 0.0402 | 0.0402 | 0.0403 | 0.0394 | 0.0393 | 0.0383 | 0.0384 | Color control group |
| F | 20 | 0.0404 | 0.0405 | 0.0407 | 0.0408 | 0.0429 | 0.0398 | 0.0393 | 0.0395 | |
| G | 40 | 0.0522 | 0.0498 | 0.0454 | 0.0431 | 0.0492 | 0.0594 | 0.0531 | 0.0431 | |
| H | 80 | 0.1208 | 0.0973 | 0.1365 | 0.123 | 0.124 | 0.104 | 0.1227 | 0.0995 | |

$(OD_{sample}\text{-}OD_{color\ control})_{10\ min} - (OD_{sample}\text{-}OD_{color\ control})_{0\ min}$

**(C)**

Inhibitor concentration (μM)

| | 1 | 2 | 3 | 4 | 5 | 6 |
|---|---|---|---|---|---|---|
| 0 | 0.0228 | 0.02074 | 0.01923 | 0.01534 | 0.01224 | 0.00733 |
| 20 | 0.0201 | 0.02014 | 0.01841 | 0.01325 | 0.00858 | 0.00563 |
| 40 | 0.02162 | 0.01791 | 0.01478 | 0.01263 | 0.00769 | 0.00452 |
| 80 | 0.01691 | 0.01949 | 0.01458 | 0.00989 | 0.00533 | 0.00333 |

V(Δ 475 nm/min)

**(D)**

Inhibitor concentration (μM)

| Conc. (μM) | 0.0625 | 0.125 | 0.25 | 0.5 | 1 | 2 |
|---|---|---|---|---|---|---|
| 0 | 43.859649 | 48.216008 | 52.00208 | 65.189048 | 81.699346 | 136.42565 |
| 20 | 49.751244 | 49.652433 | 54.318305 | 75.471698 | 116.55012 | 177.61989 |
| 40 | 46.253469 | 55.834729 | 67.658999 | 79.176564 | 130.03901 | 221.23894 |
| 80 | 59.136606 | 51.308363 | 68.587106 | 101.11223 | 187.61726 | 300.3003 |

1/substrate (mM) (header row); 1/V (values)

Plotting 1/V against 1/substrate concentration

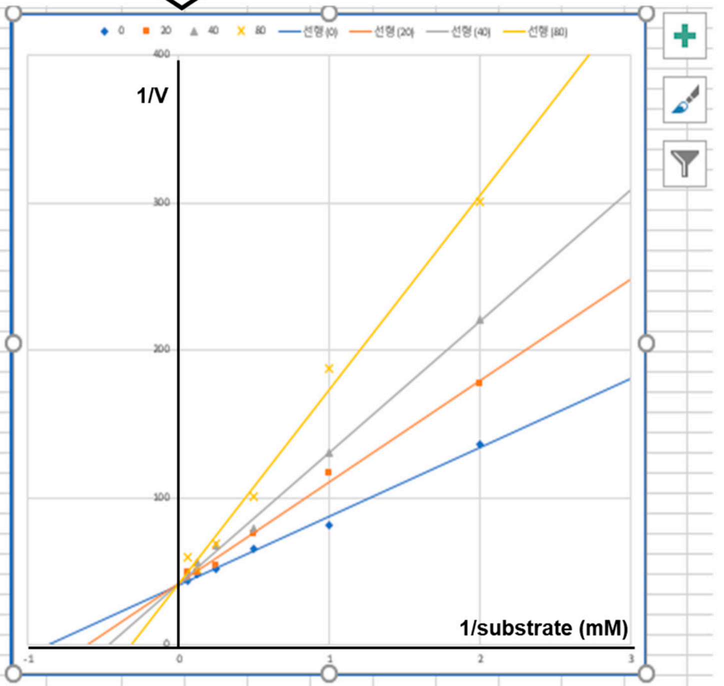


S51. Optical density values of compound **5** for 0 min (A) and 10 min (B) in a 96-well plate and values of velocity (V) per minute (min) (C) and 1/V (D) used for Lineweaver-Burk plot. The numbers 1–8 and 1–6 in the figures (A–C) represent the horizontal numbers of the 96-well plate (1: 16 mM, 2: 8 mM, 3: 4 mM, 4: 2 mM, 5: 1 mM, 6: 0.5 mM, 7: 0.25 mM, and 8: 0.125 mM for L-dopa substrate).

**(A) 0 min**

Inhibitor concentration (μM)

| | | 1 | 2 | 3 | 4 | 5 | 6 | 7 | 8 | |
|---|---|---|---|---|---|---|---|---|---|---|
| A | 0 | 0.0856 | 0.0828 | 0.075 | 0.0651 | 0.0581 | 0.0534 | 0.0458 | 0.0412 | Sample group |
| B | 20 | 0.0976 | 0.1216 | 0.0759 | 0.0683 | 0.0573 | 0.0497 | 0.0451 | 0.0415 | |
| C | 40 | 0.0967 | 0.0837 | 0.0685 | 0.0586 | 0.0546 | 0.0479 | 0.045 | 0.0395 | |
| D | 80 | 0.095 | 0.0887 | 0.0718 | 0.0621 | 0.0544 | 0.0471 | 0.0441 | 0.0416 | |
| E | 0 | 0.0393 | 0.0389 | 0.039 | 0.0392 | 0.0388 | 0.0383 | 0.0373 | 0.0384 | Color control group |
| F | 20 | 0.0391 | 0.0392 | 0.0398 | 0.0396 | 0.0377 | 0.0384 | 0.0381 | 0.0384 | |
| G | 40 | 0.0394 | 0.0397 | 0.0405 | 0.0393 | 0.0402 | 0.0398 | 0.0392 | 0.0374 | |
| H | 80 | 0.0422 | 0.0388 | 0.0397 | 0.0398 | 0.0382 | 0.0392 | 0.0393 | 0.0376 | |

**(B) 10 min**

Inhibitor concentration (μM)

| | | 1 | 2 | 3 | 4 | 5 | 6 | 7 | 8 | |
|---|---|---|---|---|---|---|---|---|---|---|
| A | 0 | 0.3291 | 0.272 | 0.2448 | 0.2088 | 0.1507 | 0.1385 | 0.0932 | 0.0684 | Sample group |
| B | 20 | 0.3021 | 0.2612 | 0.2057 | 0.1497 | 0.1047 | 0.0771 | 0.0612 | 0.0506 | |
| C | 40 | 0.2696 | 0.1997 | 0.1547 | 0.1211 | 0.0862 | 0.0671 | 0.0566 | 0.0465 | |
| D | 80 | 0.2413 | 0.1938 | 0.1376 | 0.1003 | 0.0765 | 0.0659 | 0.0542 | 0.0472 | |
| E | 0 | 0.0396 | 0.0392 | 0.0393 | 0.0394 | 0.039 | 0.0387 | 0.0375 | 0.0384 | Color control group |
| F | 20 | 0.0393 | 0.0395 | 0.0401 | 0.0398 | 0.038 | 0.0386 | 0.0383 | 0.0387 | |
| G | 40 | 0.0396 | 0.0401 | 0.0406 | 0.0396 | 0.0404 | 0.0407 | 0.0395 | 0.0378 | |
| H | 80 | 0.0437 | 0.0391 | 0.04 | 0.0401 | 0.0386 | 0.0391 | 0.0391 | 0.0378 | |

$(OD_{sample}\text{-}OD_{color\ control})_{10\ min} - (OD_{sample}\text{-}OD_{color\ control})_{0\ min}$

**(C)**

Inhibitor concentration (μM)

| | 1 | 2 | 3 | 4 | 5 |
|---|---|---|---|---|---|
| 0 | 0.02432 | 0.01889 | 0.01695 | 0.01435 | 0.00924 |
| 20 | 0.02043 | 0.01393 | 0.01295 | 0.00812 | 0.00471 |
| 40 | 0.01727 | 0.01156 | 0.00861 | 0.00622 | 0.00314 |
| 80 | 0.01448 | 0.01048 | 0.00655 | 0.00379 | 0.00217 |

V(Δ 475 nm/min)

**(D)**

Inhibitor concentration (μM)

| | 0.0625 | 0.125 | 0.25 | 0.5 | 1 |
|---|---|---|---|---|---|
| 0 | 41.1184 | 52.9381 | 58.9971 | 69.6864 | 108.225 |
| 0.5 | 48.9476 | 71.7875 | 77.2201 | 123.153 | 212.314 |
| 1 | 57.9039 | 86.5052 | 116.144 | 160.772 | 318.471 |
| 2 | 69.0608 | 95.4198 | 152.672 | 263.852 | 460.829 |

← 1/substrate (mM)

← 1/V

Plotting 1/V against 1/substrate concentration

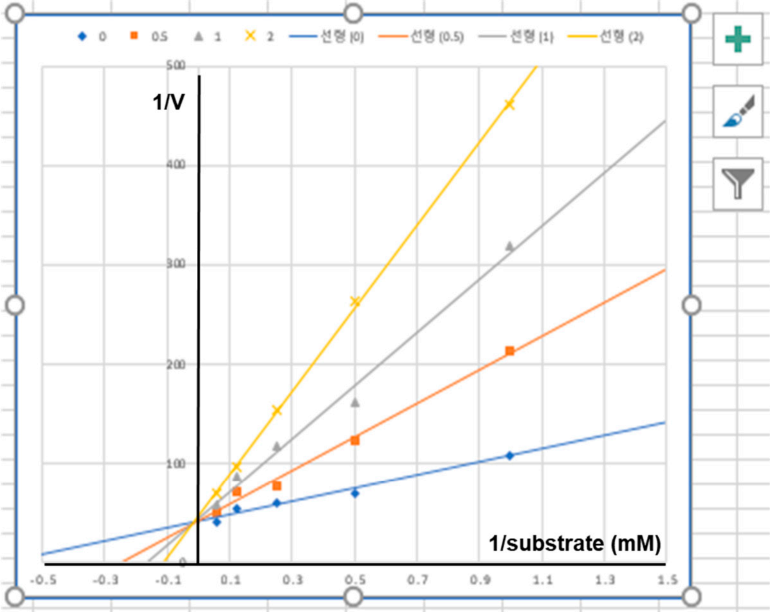


S52. Optical density values of compound **9** for 0 min (A) and 10 min (B) in a 96-well plate and values of velocity (V) per minute (min) (C) and 1/V (D) used for Lineweaver-Burk plot. The numbers 1–8 and 1–5 in the figures (A–C) represent the horizontal numbers of the 96-well plate (1: 16 mM, 2: 8 mM, 3: 4 mM, 4: 2 mM, 5: 1 mM, 6: 0.5 mM, 7: 0.25 mM, and 8: 0.125 mM for L-dopa substrate).

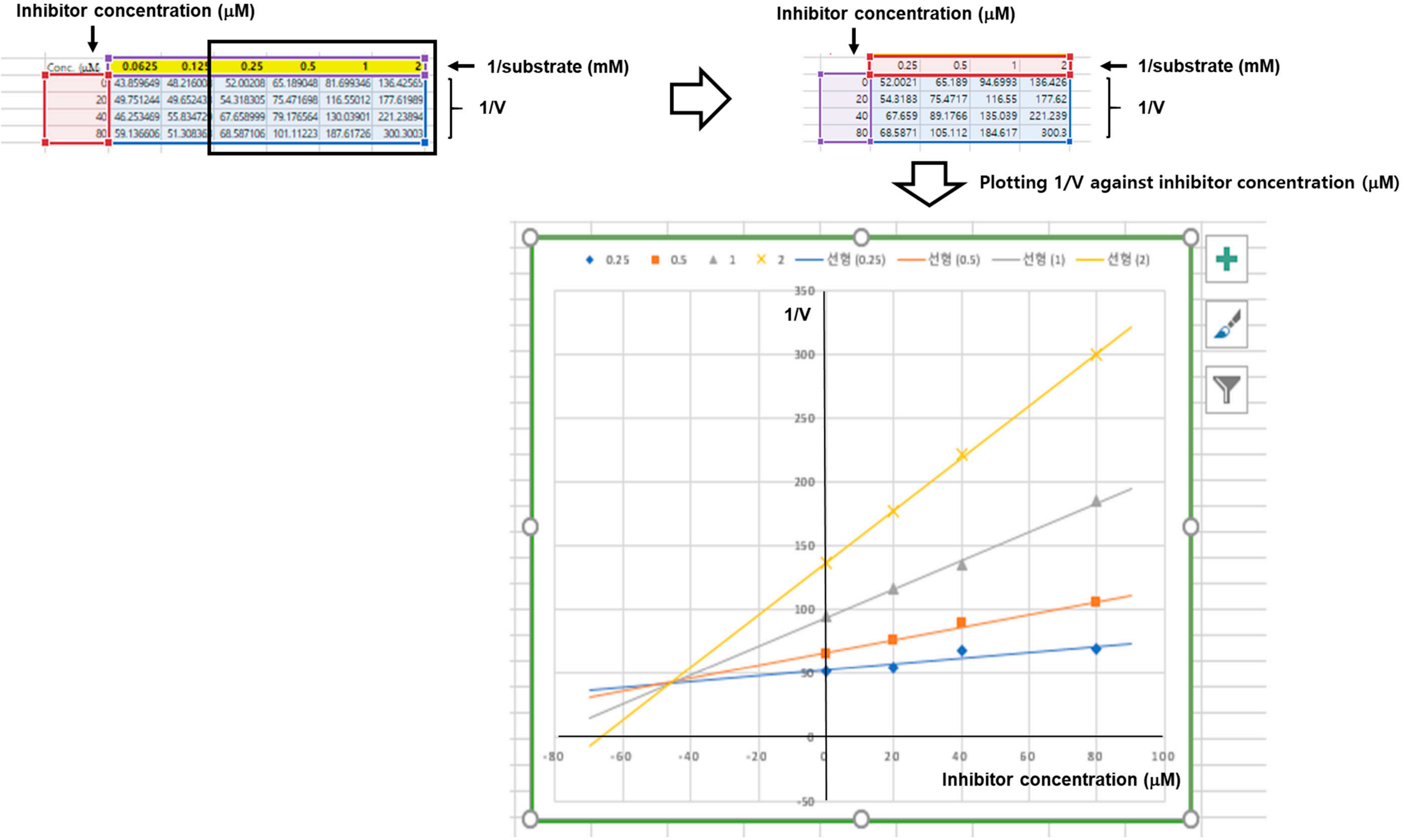


S53. Values used for Dixon plot of compound **5**.

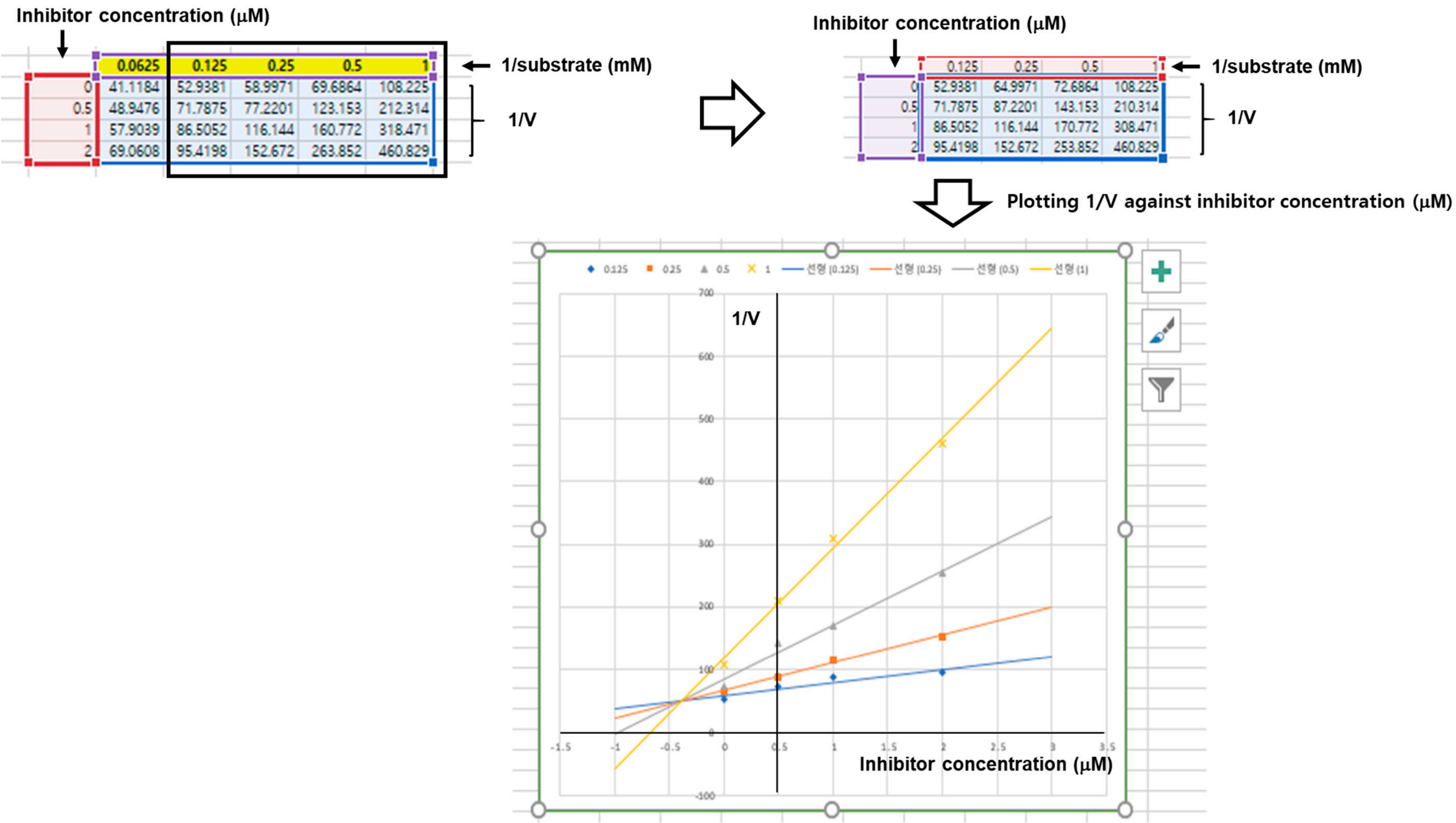

| | 0.0625 | 0.125 | 0.25 | 0.5 | 1 |
|---|---|---|---|---|---|
| 0 | 41.1184 | 52.9381 | 58.9971 | 69.6864 | 108.225 |
| 0.5 | 48.9476 | 71.7875 | 77.2201 | 123.153 | 212.314 |
| 1 | 57.9039 | 86.5052 | 116.144 | 160.772 | 318.471 |
| 2 | 69.0608 | 95.4198 | 152.672 | 263.852 | 460.829 |

| | 0.125 | 0.25 | 0.5 | 1 |
|---|---|---|---|---|
| 0 | 52.9381 | 64.9971 | 72.6864 | 108.225 |
| 0.5 | 71.7875 | 87.2201 | 143.153 | 210.314 |
| 1 | 86.5052 | 116.144 | 170.772 | 308.471 |
| 2 | 95.4198 | 152.672 | 253.852 | 460.829 |



S54. Values used for Dixon plot of compound **9**.
